# Musculoskeletal adverse events induced by immune checkpoint inhibitors: a large-scale pharmacovigilance study

**DOI:** 10.3389/fphar.2023.1199031

**Published:** 2023-10-10

**Authors:** Hao Liu, Yumin Li, Jie Li, Qiongchi Zhang, Jingtao Wu, Xinyu Li, Liesu Meng, Shuai Cao, Haopeng Li

**Affiliations:** ^1^ Department of Orthopedics, Second Affiliated Hospital, Xi’an Jiaotong University, Xi’an, China; ^2^ Department of Orthopedics, Civil Aviation General Hospital, Beijing, China; ^3^ National Joint Engineering Research Center of Biodiagnostics and Biotherapy, Second Affiliated Hospital, Xi’an Jiaotong University, Xi’an, China

**Keywords:** immune checkpoint inhibitors, musculoskeletal adverse events, toxicity profile, clinical characteristics, comorbidity, combination therapy

## Abstract

**Background:** The musculoskeletal toxicity of immune checkpoint inhibitors (ICIs) is receiving increasing attention with clinical experience. Nevertheless, the absence of a systematic investigation into the musculoskeletal toxicity profile of ICIs currently results in the under-recognition of associated adverse events. Further and more comprehensive investigations are warranted to delineate the musculoskeletal toxicity profile of ICIs and characterize these adverse events.

**Material and methods:** The present study employed the FDA Adverse Event Reporting System database to collect adverse events between January 2010 and March 2021. We utilized both the reporting odds ratio and the Bayesian confidence propagation neural network algorithms to identify suspected musculoskeletal adverse events induced by ICIs. Subsequently, the clinical characteristics and comorbidities of the major musculoskeletal adverse events were analyzed. The risk of causing these events with combination therapy *versus* monotherapy was compared using logistic regression model and Ω shrinkage measure model.

**Results:** The musculoskeletal toxicity induced by ICIs primarily involves muscle tissue, including neuromuscular junctions, fascia, tendons, and tendon sheaths, as well as joints, spine, and bones, including cartilage. The toxicity profile of PD-1/PD-L1 and CTLA-4 inhibitors varies, wherein the PD-1 inhibitor pembrolizumab exhibits a heightened overall risk of inducing musculoskeletal adverse events. The major ICIs-induce musculoskeletal adverse events, encompassing conditions such as myositis, neuromyopathy (including myasthenia gravis, Lambert-Eaton myasthenic syndrome, Guillain-Barré syndrome, and Chronic inflammatory demyelinating polyradiculoneuropathy), arthritis, fractures, myelitis, spinal stenosis, Sjogren’s syndrome, fasciitis, tenosynovitis, rhabdomyolysis, rheumatoid myalgia, and chondrocalcinosis. Our study provides clinical characteristics and comorbidities of the major ICIs-induced musculoskeletal adverse events. Furthermore, the combination therapy of nivolumab and ipilimumab does not result in a statistically significant escalation of the risk associated with the major musculoskeletal adverse events.

**Conclusion:** Immune checkpoint inhibitors administration triggers a range of musculoskeletal adverse events, warranting the optimization of their management during clinical practice.

## 1 Introduction

Cancer represents a significant global public health challenge, inflicting a considerable burden on both human health and socioeconomic status (Siegel et al., 2021). Although significant progress has been made in chemoradiation and targeted therapy, along with advancements in surgical techniques and management of cancer patients in recent decades, the survival benefit for cancer patients remains unsatisfactory (Torre et al., 2016). Encouragingly, the implementation of cancer immunotherapy has provided specific cancer patients with prolonged survival benefits, clinical recoveries, and the recognition of this achievement as one of the “Top 10 Scientific Breakthroughs” by Science magazine in 2013 (Couzin-Frankel, 2013). In physiological conditions, T-cell recognize and eliminate tumor cells within the human body through the tumor immune cycle (Waldman et al., 2020). However, tumor cells are capable of escaping immune recognition through immunoediting, troubling tumor therapy (O'Donnell et al., 2019). Immune checkpoint inhibitors (ICIs) have presented a strategy in cancer immunotherapy for reversing the immunosuppressive microenvironment, preventing cancer cells from hijacking the immune system as a “shield” for evading immune attack, and allowing the immune system to reassert its ability to kill cancer cells, resulting in substantial therapeutic gains in cancer management (Waldman et al., 2020; Carlino et al., 2021).

ICIs abrogate the negative regulation of tumor immunity, but also elicit immunotoxicity and inevitably induce immune-related adverse events (irAEs) (Kennedy and salama, 2020). The administration of ICIs can lead to unforeseeable adverse events (AEs) that may necessitate cessation of treatment, extend hospitalization, engender disability, and potentially precipitate mortality. And The resumption of ICIs following treatment interruption due to adverse events raises the likelihood of subsequent toxicity (Bylsma et al., 2022). The occurrence of AEs severely constrains their potential for cancer therapy. Theoretically, AEs resulting from ICIs may span from physical discomfort to fatality, constituting a spectrum of adverse events encompassing all tissues and organs (Ramos-Casals et al., 2020). Due to the heightened toxicity and poorer clinical outcomes in several specific locations, adverse events pertaining to these locations are accorded greater priority for investigation. As clinical experience accumulates, musculoskeletal adverse events have progressively drawn the attention of oncologists (Adda et al., 2021; Melia et al., 2023).

A substantial number of inflammatory arthritis events associated with ICIs have been reported (Xu et al., 2018; Ramos-Casals et al., 2020). Nevertheless, our investigation has also identified many non-inflammatory arthritic conditions that have been observed to manifest in ICI recipients. Previous research may have underestimated these conditions due to their infrequent occurrence. Therefore, further and more systematic investigations are warranted to comprehensively delineate the musculoskeletal toxicity profile of ICIs, thereby enabling the refinement of strategies for managing adverse events. Previous studies on ICIs’ musculoskeletal toxicity have often been conducted on small sample sizes, single-center design, and specific event-driven approaches, potentially limiting the comprehensive characterization of ICIs’ musculoskeletal toxicities. Pundole et al. employed the FAERS database for pharmacovigilance to investigate the toxicity profile of ICIs in the context of rheumatological and musculoskeletal disorders, contributing to an enhanced understanding of the musculoskeletal toxicity spectrum associated with ICIs (Pundole et al., 2019). In comparison to the study conducted by Pundole et al., we have expanded the dataset further and comprehensively explored the musculoskeletal toxicity spectrum utilizing the Standardized MedDRA Query (SMQ) system. Our analysis was constrained to encompass only primary suspected drug-adverse event pairs, and notably, the study cohort exclusively comprised cancer patients. This deliberate restriction served the purpose of pseudo-normalizing the disease biology, thereby facilitating a more profound and precise analytical approach.

The FDA Adverse Event Reporting System (FAERS) database is a global repository for adverse event reports, rendering it a crucial pharmacovigilance resource for post-marketing drug surveillance (Carnovale et al., 2020). Our study comprehensive and systematic disclosure of the characteristics of adverse effects associated with ICIs for musculoskeletal, utilizing large-scale real-world data across pan-cancer.

## 2 Material and methods

### 2.1 Data source

Using the FAERS database, we collected AE reports across pan-cancer from January 2010 to March 2021 (https://www.fda.gov/drugs/drug-approvals-and-databases/fda-adverse-event-reporting-system-faers). The Drugbank database (https://go.drugbank.com) was used to obtain the generic and trade names of seven FDA-approved ICIs, including PD-1/PD-L1 inhibitors Atezolizumab, Avelumab, Cemiplimab, Durvalumab, Nivolumab, Pembrolizumab, and CTL-4 inhibitor Ipilimumab. We applied for access to Medical Dictionary of Medical Regulatory Activities (MedDRA^®^) 25.0 (https://www.meddra.org) to facilitate data analysis procedures, as AEs in the FAERS database were encoded using the MedDRA Preferred Terms (PTs) (Dong et al., 2022).

### 2.2 Data wrangling

The FAERS database provides seven data tables that document demographic, drug, indication, adverse effect, treatment, source of the report, and outcome information for each AE report. Each report is assigned a unique identifier known as “primaryid.”. We imported all tables into R software version 4.1.2 and performed data merging using the “primaryid.” to obtain comprehensive information for each AE report. The FAERS database categorizes each drug-AE pair into one of four categories based on the role of the drug in inducing the adverse event: Primary Suspect (PS), Secondary Suspect (SS), Concomitant (C), and Interacting (I). To focus our analysis, we restricted it to Drug-AE pairs designated as “primary suspect” (Strandell et al., 2011). We also followed FAERS recommendations and removed quarterly inaccurate reports. Given that the same report may be recorded multiple times, we utilized the “primary” to eliminate duplicate reports. We applied filters using the generic and brand names of seven ICIs to screen out target reports, and to mitigate data loss resulting from misspellings or unexpected name variations, we additionally used the ‘active ingredient’ field as an ancillary reference. The screened reports underwent data standardization, which included standardizing patient age units to “years” and substituting “01″for any missing day in the date, following FAERS recommendations (Dong et al., 2022). We procured information concerning the systems and organs implicated in each adverse event report in accordance with the MedDRA 25.0 System and Organ Classification (SOC) standard. Through referring to the preferred terms (PTs) in Standardized MedDRA Queries (SMQs) of MedDRA 25.0, we successfully identified and screened AEs associated with musculoskeletal induced by ICIs from all drug-AE pairs.

### 2.3 Pharmacovigilance analysis

Disproportionality analysis is a widely used method in pharmacovigilance analysis, where a higher rate of exposure of an AE to ICIs than to other drugs may suggest an association (Lehman et al., 2007). This study employed the reporting odds ratio (ROR) for disproportionality analysis. The ROR is not affected by non-selective underreporting of drugs or events since it is a ratio. We considered a lower bound of the two-sided 95% confidence interval of ROR >1 as a significant signal (Almenoff et al., 2007). However, the ROR may be biased due to minor sample size limitations and yield false positives from inflated values. Therefore, we also utilized the Bayesian confidence propagation neural network (BCPNN) algorithm to correct the influence of false high ROR values and enhance the dependability of the detected signals. The BCPNN algorithm produces an information component (IC) indicator, and we attributed significance to the lower bound of the two-sided 95% confidence interval of IC > 0 (Dong et al., 2022). We applied both the ROR and BCPNN algorithm to identify pharmacovigilance signals for drug-event pairs and executed the algorithm on R software version 4.1.2. Furthermore, the study cohort exclusively comprised cancer patients. This deliberate restriction served the purpose of pseudo-normalizing the disease biology, thereby facilitating a more profound and precise analytical approach. Furthermore, we referred to the definition of osteoporosis in the Standardized MedDRA Queries (SMQs) of MedDRA 25.0 and evaluated the risk of ICIs-induced osteoporosis using the following terms: osteoporosis, procedural and treatment terms related to osteoporosis, fractures of the vertebral, hip, and lower radius (fracture types with distinct osteoporotic features), post-traumatic osteoporosis exacerbations.

### 2.4 Time to onset analysis

We retained only the first report of each ICI-AE pair to determine the time frame between the initiation of ICIs treatment and the onset of AEs. Boxplots were utilized to present the median and range of time to onset. We then applied the Weibull distribution model (Leroy et al., 2014; Ando et al., 2019) to time data using Minitab Statistical Software (version 20.0, State College, PA: Minitab Inc.) to estimate the shape parameter (β). A shape parameter (β) less than 1 suggests an “early failure” distribution type, equal to 1 indicates a “random failure” distribution type, and greater than 1 indicates a “late failure” distribution type. In “late failure” cases, the probability of the adverse event increases over time, implying a pattern of toxicity accumulation. When referring to “early failure,” it suggests that there may be a susceptibility of the population to the adverse event.

### 2.5 Analysis of comorbidities

FAERS provides a unique identifier called “caseid” for each case reported to the system. In addition, for each case, FAERS also assigns a “Caseversion” to all reports related to that case, allowing for tracking of changes or updates to the case over time. The initial report of a case will be version 1, and subsequent reports of the case will have successively increasing version numbers. We conducted an association analysis by tracking all adverse events reported in each case’s initial and follow-up reports.

### 2.6 Pharmacovigilance analysis of combination therapy

To compare the risk of AEs induced by combination therapy *versus* monotherapy, we conducted a logistic regression analysis using SPSS (version 22.0, IBM Corp., Armonk, NY) and adjusted for covariates, including age, gender, and tumor type. Ω Shrinkage Measure Model was employed to further investigate the drug-drug interaction signals (DDIs) (Noguchi et al., 2019).

### 2.7 Statistical analysis and visualization of results

The Chi-square test was employed for analyzing categorical data. In contrast, Fisher’s exact test was utilized in cases where the sample size was less than 40 or if any theoretical frequency in the 2 × 2 contingency table was less than 5. For analyzing quantitative data, the independent-sample *t*-test was applied. In cases where the quantitative data did not follow a normal distribution, the Mann-Whitney *U* test was employed. The Welch’s *t*-test was utilized when the quantitative data did not meet the homogeneity of variance assumption. We performed all statistical analyses using R software version 4.1.2. The study results were visualized using the R package “ggplot2” (Gómez-Rubio V, 2017). A bilateral *p*-value was used, with a significance level of *p* < 0.05.

## 3 Result

**FIGURE 1:**
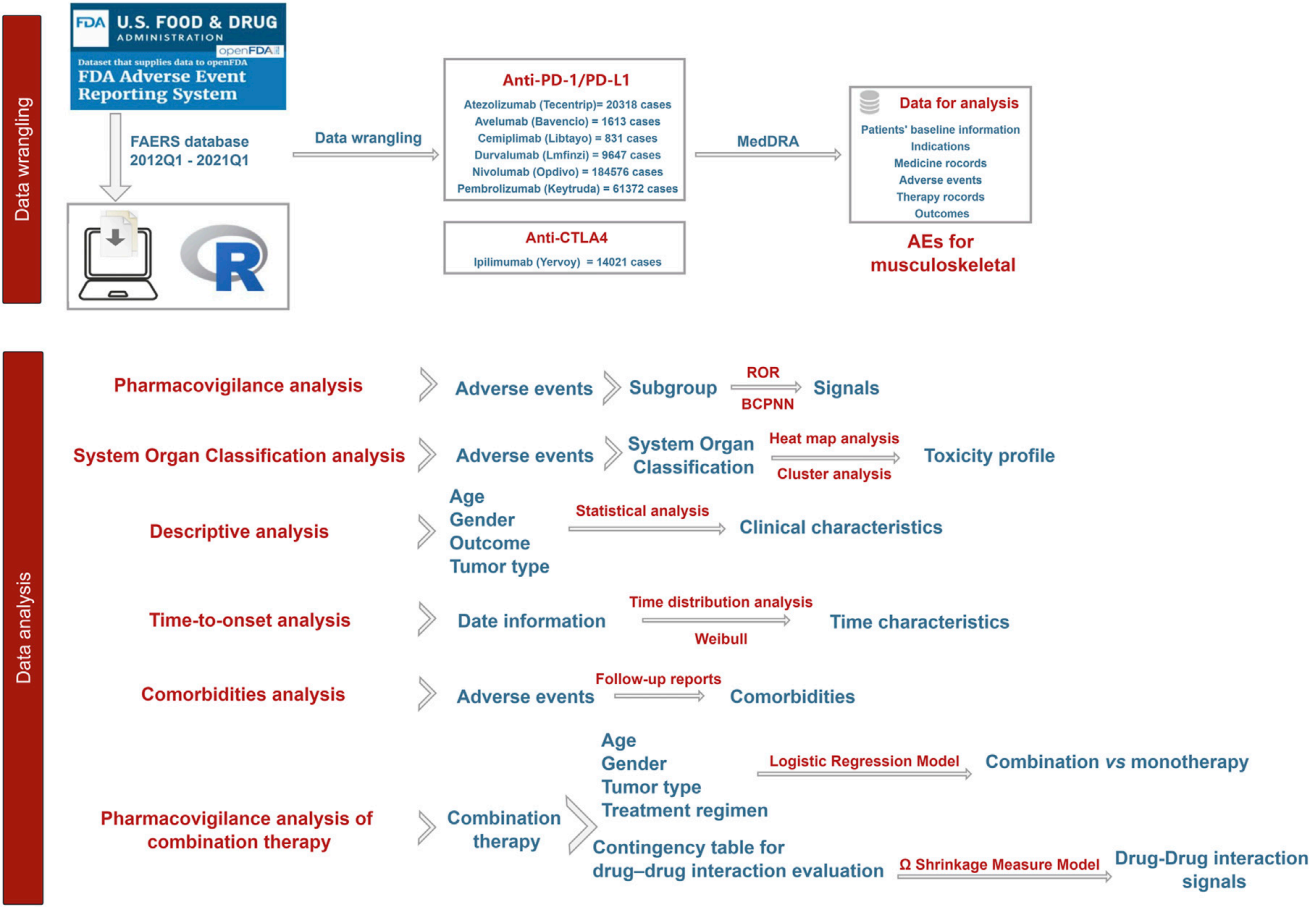
The flowchart of the present study.

Figure 1 depicts the workflow of the present study. Following data cleaning, a total of 292,378 AE reports, classified as “Primary suspect,” were gathered for atezolizumab (20,318 reports), avelumab (1,613 reports), cemiplimab (831 reports), durvalumab (9,647 reports), ipilimumab (14,021 reports), nivolumab (184,576 reports), and pembrolizumab (61,372 reports).

### 3.1 Results of system organ classification analysis

**FIGURE 2:**
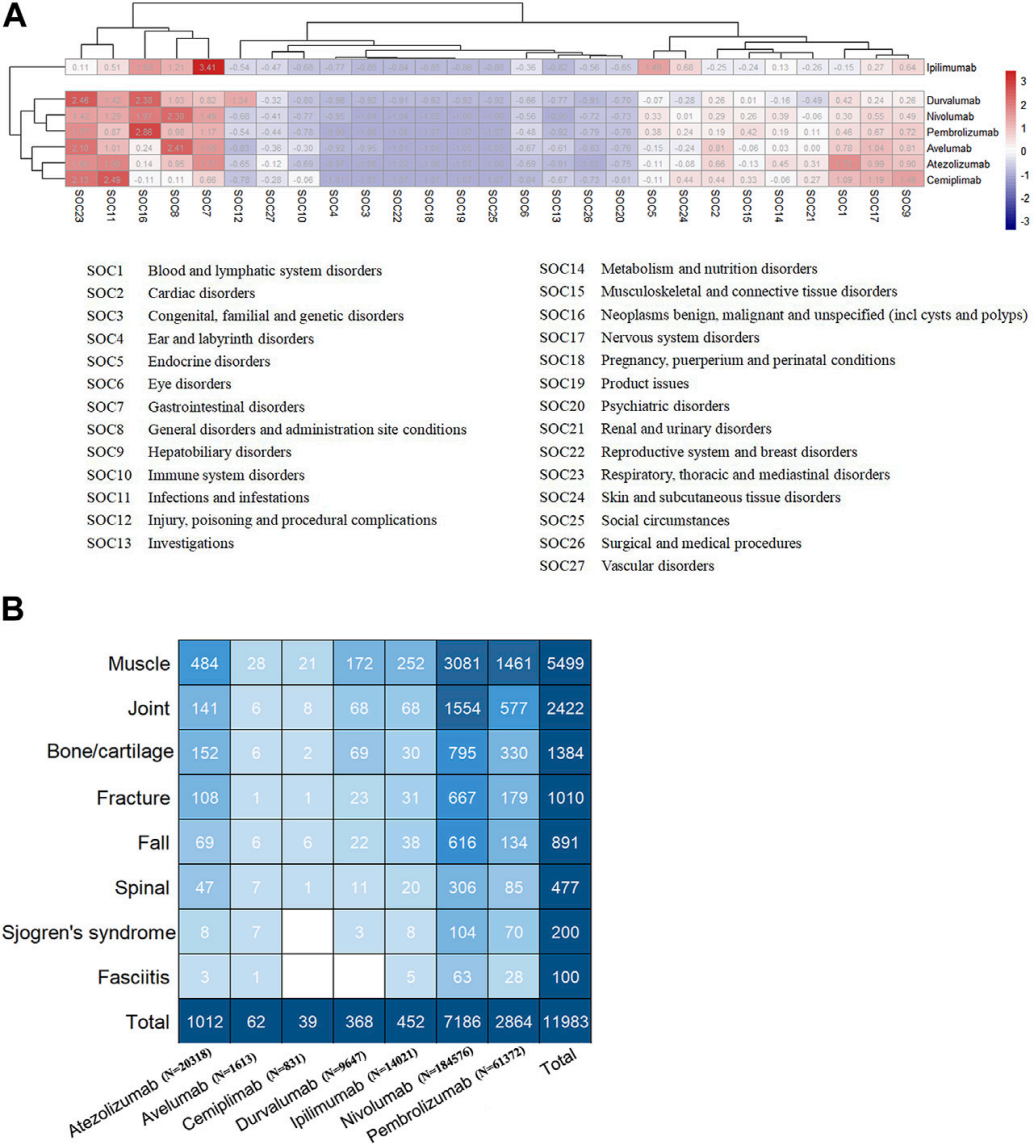
**(A)** The heatmap analysis and cluster analysis (employing K-means method) based on the number of AEs involving each SOC **(B)** The subgroup of ICIs-induced AEs in musculoskeletal, with numbers within the square representing the corresponding AEs count.

An analysis of system and organ involvement patterns in ICIs-induced AEs was conducted. The findings depicted in Figure 2A indicate that ICIs-induced AEs exhibit a higher incidence of involvement in gastrointestinal disorder, respiratory, thoracic and mediastinal disorder, infection and infestation, nervous system disorder, hepatobiliary disorder, blood and lymphatic system disorder, followed by renal and urinary disorder, cardiac disorder, skin and subcutaneous tissue disorders, endocrine disorder, metabolism and nutrition disorder, and musculoskeletal and connective tissue disorder. Patients undergoing ICIs treatment have reported a significant number of AEs in the aforementioned locations. Moreover, it is noteworthy that utilizing clustering analysis to examine the patterns of SOC involvement in ICIs-induced AEs allows for effective differentiation between PD-1/PD-L1 inhibitors (atezolizumab, avelumab, cemiplimab, durvalumab, nivolumab, and pembrolizumab) and CTLA-4 inhibitor (ipilimumab), a distinction that bears particular intrigue (Figure 2A). The divergent patterns of SOC involvement imply disparate mechanisms underlying the toxicity induced by these agents. In contrast to PD-1/PD-L1 inhibitors, the CTLA-4 inhibitor exhibits a higher propensity to elicit endocrine diseases, skin and subcutaneous tissue disorders, and gastrointestinal disorders, while displaying a comparatively lower propensity to induce cardiac and hematologic and lymphatic diseases (Figure 2A). Furthermore, immune checkpoint inhibitors have varying toxicity levels in musculoskeletal and connective tissue disorders. Specifically, pembrolizumab demonstrates the highest toxicity in this regard, while ipilimumab exhibits the lowest toxicity.

### 3.2 The major ICIs-induced musculoskeletal AEs

**FIGURE 3:**
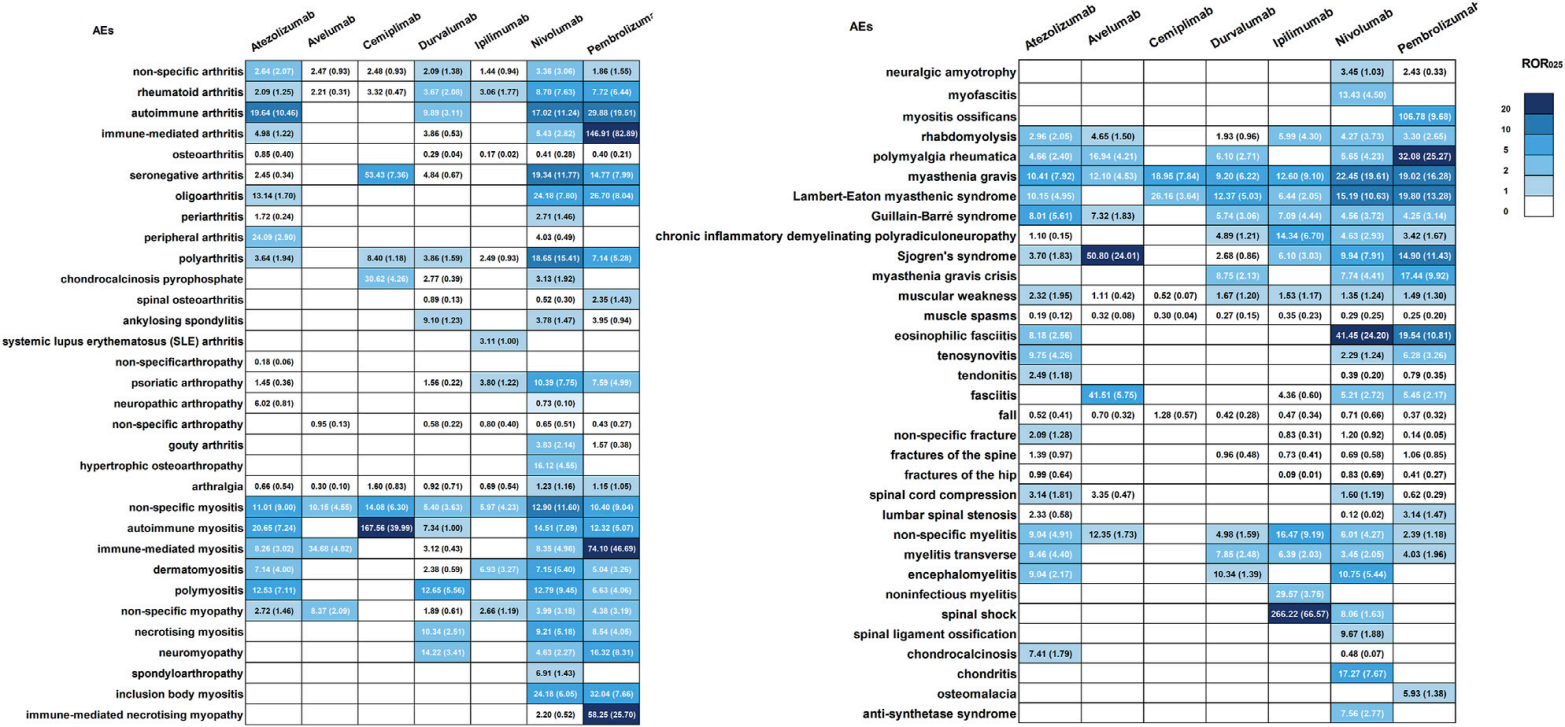
Signals of the major ICIs-induced AEs in musculoskeletal. The values within each cell indicate both the signal value and the corresponding lower limit of its 95%CI.

Figure 2B displays that the musculoskeletal toxicity induced by ICIs primarily involves muscle tissue, including neuromuscular junctions, fascia, tendons, tendon sheaths, joints, spine, and bones, including cartilage. Supplementary Table S1 provides detailed information on ICIs-induced musculoskeletal AEs, whereas Supplementary Table S2 outlines the distribution of these AEs concerning age, gender, and tumor type. Myositis, neuromyopathy (including myasthenia gravis, Lambert-Eaton myasthenic syndrome, Guillain-Barré syndrome, Chronic inflammatory demyelinating polyradiculoneuropathy), rhabdomyolysis, arthritis, fracture, myelitis, spinal stenosis, Sjogren’s syndrome, fasciitis, tenosynovitis, rhabdomyolysis, rheumatoid myalgia, and chondrocalcinosis are the major ICIs-induced musculoskeletal AEs (Supplementary Table S1). Figure 3 exhibits the signals of the major ICIs-induced musculoskeletal AEs.

#### 3.2.1 Myositis

A total of 1,267 cases of ICIs-induced myositis are included in this study. All ICIs demonstrate a substantial risk of inducing myositis (Supplementary Table S1, Figure 3). Cemiplimab carries a high risk of inducing autoimmune myositis, while atezolizumab is most strongly associated with this condition, trailed by polymyositis and immune-mediated myositis. Durvalumab is significantly associated with polymyositis, while ipilimumab is significantly associated with dermatomyositis. Nivolumab is most linked to inclusion body myositis, followed by autoimmune myositis, polymyositis, necrotizing myositis, immune-mediated myositis, and dermatomyositis. Pembrolizumab exhibits the strongest association with immune-mediated myositis, followed by autoimmune myositis, dermatomyositis, polymyositis, necrotizing myositis, and inclusion body myositis. Additionally, numerous reports of symptoms resembling myositis have also been collected and are detailed in Supplementary Table S3. A substantial proportion of ICIs recipients who reported myositis also develop myocarditis throughout follow-up, particularly in cases of autoimmune and immune-mediated myositis (54% and 36%, respectively). Furthermore, myasthenia gravis, abnormal liver function, respiratory disorder, and rhabdomyolysis are highly correlated with any type of myositis as comorbidities. In addition, pneumonia and interstitial lung disease have been reported in 16% of dermatomyositis cases (Table 1).

**TABLE 1:** The results of comorbidity analysis.

Subgroup	AEs	Comorbidities	Ratio^a^ (%)
Myositis	Non-specific myositis	Myocarditis (predominantly immune-mediated myocarditis and autoimmune myocarditis)	32
		Myasthenia gravis and Myasthenia gravis crisis	24
		Abnormal liver function (64 cases hepatitis, 47 cases hepatic function abnormal, 3 cases hepatic failure)	21
		Respiratory disorder (45 cases respiratory failure, 28 cases dyspnea, 5 cases respiratory distress)	14
		Rhabdomyolysis	11
	Polymyositis	Abnormal liver function	30
		Pneumonia and Pneumonitis	21
		Myocarditis	20
		Myasthenia gravis and Myasthenia gravis crisis	20
		Rhabdomyolysis	15
	Dermatomyositis	Interstitial lung disease and Pneumonia	16
		Abnormal liver function	16
		Psoriasis	7
	Immune-mediated myositis	Myocarditis (predominantly immune-mediated myocarditis and autoimmune myocarditis)	36
		Myasthenia gravis and Myasthenia gravis crisis	29
		Abnormal liver function	26
		Rhabdomyolysis	10
		Atrioventricular block complete	7
	Autoimmune myositis	Myocarditis (predominantly autoimmune myocarditis)	54
		Respiratory failure	31
		Abnormal liver function	23
		Myasthenia gravis	23
		Rhabdomyolysis	15
	Necrotizing myositis	Rhabdomyolysis	27
		Myocarditis (predominantly autoimmune myocarditis)	20
		Respiratory failure	13
Myopathy	Non-specific myopathy	Immune-mediated adverse events (including myocarditis, hepatitis, enterocolitis)	20
		Thyroid disorder (predominantly hypothyroidism)	17
		Myositis (predominantly immune-mediated myositis)	17
		Abnormal liver function	17
	Neuromyopathy	Eyelid ptosis	18
		Myositis	18
		Immune-mediated adverse events	18
		Abnormal liver function	18
	Immune-mediated necrotizing myopathy	Myositis (predominantly immune-mediated myositis)	63
		Myasthenia gravis	25
Myasthenia gravis	Myasthenia gravis	Myositis (predominantly immune-mediated myositis)	38
		Myocarditis (predominantly immune-mediated myocarditis)	27
		Respiratory disorder (44 cases respiratory failure, 20 cases dyspnea, 6 cases respiratory distress)	18
		Abnormal liver function (36 hepatic function abnormal, 34cases hepatitis, 1 hepatic failure)	18
		Rhabdomyolysis	7
Lambert-Eaton myasthenic syndrome	Lambert-Eaton myasthenic syndrome	Respiratory failure	23
		Myositis (predominantly immune-mediated myositis)	19
		Myocarditis (predominantly immune-mediated myocarditis)	16
Arthritis	Non-specific arthritis	Thyroid disorder (predominantly hypothyroidism)	15
		Colitis	13
		Rash	10
		Diarrhoea	8
		Pyrexia	8
	Rheumatoid arthritis	Interstitial lung disease and Pneumonia	12
		Colitis	11
		Rash	11
		Diarrhoea	8
		Pyrexia	7
	Autoimmune arthritis	Thyroid disorder (predominantly hypothyroidism)	26
		Headache	13
		Autoimmune pancreatitis	10
	Immune-mediated arthritis	Rash	18
		Pyrexia	10
	Polyarthritis	Immune-mediated adverse reaction	14
		Colitis	9
	Osteoarthritis	Pneumonia and Pneumonitis	19
		Arteriosclerosis (5 cases aortic arteriosclerosis, 3 case arteriosclerosis coronary artery)	17
		Rash	13
Fracture	Non-specific fractures	fall	33
	Osteoporotic type fractures	fall	32
	Other fractures	fall	36
Myelitis	Non-specific Myelitis	Peripheral nerve toxicity (12 cases neuropathy peripheral, 4 cases Guillain-Barré syndrome, 1 cases myasthenia gravis)	30
		Encephalitis and Meningitis (4 cases meningitis aseptic, 3 cases encephalitis, 1case autoimmune encephalitis)	14
Rhabdomyolysis	Rhabdomyolysis	Myositis (predominantly polymyositis and immune-mediated myositis)	33
		Myocarditis (predominantly autoimmune myocarditis)	21
		Renal injury (23 cases acute kidney injury, 2 cases renal failure, 7 cases renal disorder)	21
		Abnormal liver function (24 cases hepatic function abnormal, 18 cases hepatitis, 5 cases hepatic failure)	20
		Myasthenia gravis	12
		Thyroid disorder (predominantly hypothyroidism)	10
Polymyalgia rheumatica	Polymyalgia rheumatica	Immune-mediated adverse reaction	21
		Arthritis (predominantly polyarthritis)	13
Fasciitis	Eosinophilic	Cholangitis	14

^a^ Ratio refers to the proportion of reports indicating corresponding comorbidity out of all reports of adverse events.

#### 3.2.2 Myopathy

We collect 189 cases of ICIs-induced myopathy. Atezolizumab, nivolumab, pembrolizumab, and ipilimumab exhibit a risk of inducing myopathy (Supplementary Table S1, Figure 3). Nivolumab is primarily linked with neuromyopathy and mitochondrial myopathy, whereas pembrolizumab is primarily associated with neuromyopathy, followed by immune-mediated necrotizing myopathy. It should be noted that using the term “neuromyopathy” may not be adequate for assessing the risk of ICIs-induced neuromyopathy, as other related terms that describe neuromyopathy, including myasthenia gravis, Lambert-Eaton myasthenic syndrome, Guillain-Barré syndrome, and chronic inflammatory demyelinating polyradiculoneuropathy, have also been documented. These terms were employed to assess the potential risk of ICIs-induced neuromyopathy further. A total of 782 cases of myasthenia gravis were reported, with all ICIs demonstrating a significant risk of inducing the condition (Supplementary Table S1, Figure 3). Pembrolizumab and nivolumab exhibit the strongest association with myasthenia gravis (ROR_025_ = 19.61, IC_025_ = 3.40; ROR_025_ = 19.02, IC_025_ = 3.57). Moreover, nivolumab, pembrolizumab, and durvalumab are each reported to have 16, 16, and 2 cases of myasthenia gravis crisis, respectively, representing approximately 4.6% of patients with myasthenia gravis experiencing a myasthenic crisis. Pembrolizumab recipients demonstrate the highest risk of experiencing myasthenia gravis crisis (ROR_025_ = 9.92, IC_025_ = 2.51). A total of 99 cases of Lambert-Eaton myasthenic syndrome are identified, with a significant risk of induction by atezolizumab, durvalumab, ipilimumab, nivolumab, and pembrolizumab (Supplementary Table S1, Figure 3). Among them, pembrolizumab exhibits the strongest association with Lambert-Eaton myasthenic syndrome (ROR_025_ = 13.28, IC_025_ = 3.06), followed by nivolumab (ROR_025_ = 10.63, IC_025_ = 2.68). We identify a total of 217 cases of Guillain-Barré syndrome. All ICIs carry a risk of inducing this condition, except for cemiplimab and Avelumab (Supplementary Table S1, Figure 3). Atezolizumab demonstrates the strongest association (ROR_025_ = 5.61, IC_025_ = 2.27), followed by ipilimumab (ROR_025_ = 4.44, IC_025_ = 1.90). We identify 40 cases of chronic inflammatory demyelinating polyradiculoneuropathy. Avelumab and cemiplimab do not have any reported cases of this condition, while durvalumab is not statistically associated with it (Supplementary Table S1, Figure 3). Among the other ICIs, ipilimumab has the highest risk of inducing it (ROR_025_ = 6.70, IC_025_ = 1.82), followed by nivolumab (ROR_025_ = 2.93, IC_025_ = 0.51).

The above findings suggest the toxicity of ICIs in neuromuscular junctions, resulting in a significant risk of inducing neuromyopathy. It is worth noting that individuals receiving atezolizumab, nivolumab, pembrolizumab, durvalumab, avelumab, and ipilimumab may experience muscle weakness and muscle spasms (Supplementary Table S1, Figure 3). Given that both of them can be clinical manifestations of myositis and myopathy, these findings provide further evidence of the considerable risk that ICIs pose in inducing myositis and myopathy. In patients with non-specific myopathy, immune-mediated adverse events (20%) have the strongest correlation as a comorbidity, followed by thyroid disease (17%, predominantly hypothyroidism), myositis (17%), and abnormal liver function (17%). For Neuromyopathy, blepharoptosis is the most prevalent comorbidity (18%), while immune-mediated adverse events, myositis, and abnormal liver function remain significant comorbidities. Immune-mediated necrotizing myopathy recognizes immune-mediated myositis and myasthenia gravis as strongly associated comorbidities. Comorbidities are common in myasthenia gravis cases, with myositis reported in 38% of cases (predominantly immune-mediated myositis), and myocarditis in 27% of cases, while respiratory disorders, abnormal liver function, and rhabdomyolysis are reported in 18%, 18%, and 7% of cases, respectively. Lambert-Eaton myasthenic syndrome commonly presents with myositis (19%) and myocarditis (16%) as comorbidities. However, no significant comorbidities are associated with Guillain-Barré syndrome and chronic inflammatory demyelinating polyradiculoneuropathy (Table 1).

#### 3.2.3 Arthritis

We amass 1782 cases of ICIs-induced arthritis. Pembrolizumab, nivolumab, and atezolizumab exhibit a considerably higher probability of arthritis induction in comparison to ipilimumab and durvalumab. Conversely, cemiplimab and avelumab demonstrate no statistically significant correlation with arthritis. Atezolizumab is most strongly linked with autoimmune arthritis, followed by polyarthritis and rheumatoid arthritis. Nivolumab is significantly associated with autoimmune arthritis, rheumatoid arthritis, immune-mediated arthritis, polyarthritis, oligoarthritis, gouty arthritis, and periarthritis. Furthermore, a correlation has been observed between nivolumab and ankylosing spondylitis, a relationship not detected in other ICIs. Pembrolizumab is strongly associated with immune-mediated arthritis, followed by autoimmune arthritis, oligoarthritis, rheumatoid arthritis, polyarthritis, and spinal osteoarthritis. Durvalumab is primarily associated with autoimmune arthritis, and is also statistically significant in cases of polyarthritis and rheumatoid arthritis. Ipilimumab is primarily associated with rheumatoid arthritis. While there have been numerous reported cases of ICIs-induced osteoarthritis, including spinal osteoarthritis, a significant correlation has only been found between pembrolizumab and the development of these conditions. Notably, nivolumab and pembrolizumab are associated with the term “seronegative arthritis.” However, this association should not be extended to specific subtypes of seronegative arthritis, such as psoriatic arthritis, reactive arthritis, and spondyloarthritis (Supplementary Table S1, Figure 3). The term “seronegative arthritis” appears to be more of an alternative description for inflammatory arthritis. In addition, a considerable number of reports have documented ICIs-induced arthralgia (Supplementary Table S1), while Supplementary Table S3 provides a comprehensive account of other documented symptoms resembling arthritis. A thyroid disorder, mainly hypothyroidism, is the most prevalent comorbidity (15%) associated with non-specific arthritis, with colitis (13%), rash (10%), diarrhea (8%), and fever (8%) following closely. Additionally, autoimmune arthritis is significantly associated with a thyroid disorder (26%), predominantly hypothyroidism. In immune-mediated arthritis cases, the rash is present in 18% of cases and fever in 10% of cases during follow-up. In rheumatoid arthritis, interstitial lung disease and pneumonia are the most prominent comorbidity (12%), followed by colitis (11%). The most prominent comorbidity in polyarthritis is immune-mediated adverse events, accounting for 14%, followed by colitis with a 9% association.

#### 3.2.4 Fracture

**FIGURE 4:**
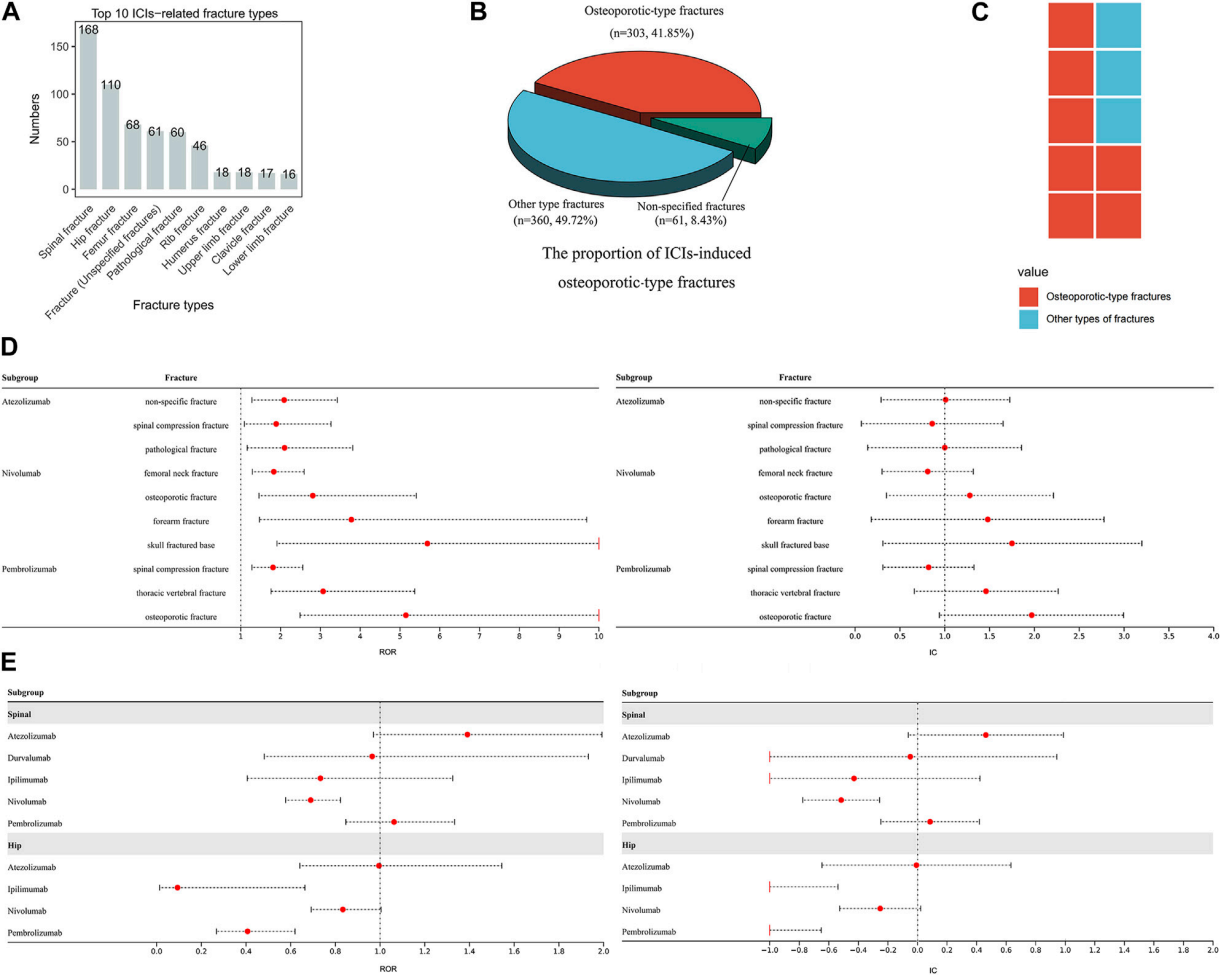
ICIs-induced fracture events **(A)** The top ten ICIs-induced fracture types **(B)** This pie chart depicts the distribution and proportion of ICIs-induced osteoporotic-type fractures, other types of fractures, and non-specific fractures **(C)** The composition of ICIs-induced fracture events with notable signals **(D)** This forest plot displays ICIs-induced fracture events with significant signals **(E)** The forest plots exhibit the risk of ICIs-induced spinal and hip fractures.

We collect 1,010 reports of ICIs-induced fractures. Notably, spinal, hip, and femoral fractures emerge as the most prevalent types (Figure 4A). Fractures demonstrating distinct osteoporotic features comprising 41.85% of all ICIs-induced fractures (Figure 4B). Although none of atezolizumab (ROR_025_ = 0.88, IC_025_ = −0.18), durvalumab (ROR_025_ = 0.62, IC_025_ = −0.70), ipilimumab (ROR_025_ = 0.41, IC_025_ = −1.27), nivolumab (ROR_025_ = 0.71, IC_025_ = −0.49), and pembrolizumab (ROR_025_ = 0.47, IC_025_ = −1.09) exhibits a significant association with overall risk of fracture, 10 ICI-fracture event pairs are detected with significant signals (Figure 4D). Notably, 7 of these pairs correspond to osteoporotic-type fractures (Figure 4C). The primary sites of positive signals for fractures are the spine and hip (Figure 4D). However, to mitigate the potential overestimation of signals caused by variations in terminology used to describe spinal and hip fractures, we consolidated cervical, thoracic, and lumbar vertebral fractures into the category of spinal fractures, and femoral neck, acetabular, and sacral fractures into the category of hip fractures, resulting in a more precise assessment of the risk of ICIs-induced spinal and hip fractures. Further analysis reveals that no significant signals are detected in ICIs-induced spinal and hip fractures, although atezolizumab and pembrolizumab are on the edge of statistical significance (Figure 4E). The comorbidity analysis reveals that fall is the most frequently reported comorbidity among both osteoporotic-type fractures and other types of fractures (accounting for approximately 30% of cases, Table 1). The aforementioned findings contribute to the hypothesis that ICIs may increase the risk of fractures by inducing osteoporosis and falls. However, the potential association between ICIs and the risk of osteoporosis and falls fails to show statistical significance. Compared with other ICIs, the association between atezolizumab and osteoporosis appears to be the most robust, while the association between nivolumab and fall is the strongest (Table 2).

**TABLE 2:** The risk of ICIs-induced osteoporosis and falls.

Risk	Drug	ROR algorithm		BCPNN algorithm	
		ROR	95%CI	IC	95%CI
Osteoporosis	Atezolizumab	0.87	0.66–1.15	−0.20	−0.61-(0.21)
	Durvalumab	0.45	0.24–0.83	−1.12	−2.01-(-0.23)
	Ipilimumab	0.39	0.23–0.66	−1.33	−2.09-(-0.57)
	Nivolumab	0.70	0.62–0.79	−0.50	−0.68-(-0.32)
	Pembrolizumab	0.77	0.64–0.93	−0.36	−0.63-(-0.10)
fall	Atezolizumab	0.52	0.41–0.66	−0.93	−1.27-(-0.58)
	Durvalumab	0.42	0.28–0.64	−1.21	−1.82-(-0.60)
	Ipilimumab	0.47	0.34–0.65	−1.07	−1.54-(-0.61)
	Nivolumab	0.71	0.66–0.77	−0.47	−0.59-(-0.36)
	Pembrolizumab	0.37	0.32–0.44	−1.39	−1.64-(-1.14)

#### 3.2.5 AEs involving the spine

Our study gathers 113 cases of ICIs-induced myelitis. Atezolizumab, nivolumab, pembrolizumab, durvalumab, and ipilimumab exhibit significant associations with non-specific myelitis and transverse myelitis. Ipilimumab exhibits the highest risk of inducing myelitis (Supplementary Table S1, Figure 3). Furthermore, we also collected 28 reports of encephalomyelitis (including 4 cases of acute disseminated encephalomyelitis) and neuromyelitis optica spectrum disorder (Supplementary Table S1). Neuromyopathy, including non-specific peripheral neuropathy, Guillain-Barré syndrome, and myasthenia gravis, represents the comorbidity most strongly associated with non-specific myelitis (30%, Table 1). Furthermore, 14% of cases with non-specific myelitis develop encephalitis or meningitis. In contrast, transverse myelitis has no prominent comorbidities, with its comorbidities more commonly involving motor dysfunction and urinary incontinence descriptions. Similarly, cases of encephalomyelitis do not exhibit any notably prominent comorbidities. Apart from myelitis, the main ICIs-induced AEs related to the spine that we identify in our data collection include spinal stenosis and spinal cord compression. Moreover, several reports document cases of spinal cord injury and spinal shock (Supplementary Table S1). Supplementary Table S3 documents additional symptoms resembling AEs involving the spine.

#### 3.2.6 Rhabdomyolysis

We collect 406 cases of ICIs-induced rhabdomyolysis. Significant associations are identified between rhabdomyolysis and ipilimumab, atezolizumab, nivolumab, pembrolizumab, and avelumab, with ipilimumab carrying the highest risk (ROR_025_ = 4.30, IC_025_ = 1.98). Immune-related myositis is the comorbidity most strongly linked to rhabdomyolysis, accounting for 33% of cases, followed by myocarditis (21%), renal injury (21%), abnormal liver function (20%), myasthenia gravis (12%), and thyroid dysfunction (10%, predominantly hypothyroidism).

#### 3.2.7 Polymyalgia rheumatica

We collect 185 cases of ICIs-induced polymyalgia rheumatica. With the exclusion of ipilimumab and cemiplimab, the remaining ICIs exhibit a notable likelihood of causing polymyalgia rheumatica, with pembrolizumab displaying the most significant risk (ROR_025_ = 25.27, IC_025_ = 3.91). The comorbidity most significantly correlated with polymyalgia rheumatica is immune-mediated adverse events (21%), trailed by arthritis (13%).

#### 3.2.8 Fasciitis

We collect 85 reports of ICIs-induced fasciitis. No significant association is found between ICIs and necrotizing fasciitis. Nevertheless, nivolumab, pembrolizumab, and atezolizumab are statistically associated with the induction of eosinophilic fasciitis, with nivolumab (ROR_025_ = 24.20, IC_025_ = 3.13) and pembrolizumab (ROR_025_ = 10.81, IC_025_ = 2.54) displaying a powerful association. Interestingly, cholangitis is observed in 14% of patients with eosinophilic fasciitis, while there is no prominent comorbidity with necrotizing fasciitis.

#### 3.2.9 Other AEs

Reports of ICIs-induced bone or cartilage diseases, mainly including osteoporosis, osteolysis, increased bone resorption and decreased bone density, osteitis, osteonecrosis, chondritis, and chondrocalcinosis. Notably, some have been found to have a significant association with the administration of ICIs (Supplementary Table S1). Additionally, we have collected reports of ICIs-induced tenosynovitis and tendinitis, whereby atezolizumab, nivolumab, and pembrolizumab have shown significant risks of inducing tenosynovitis. Supplementary Table S2 outlines the distribution of these AEs concerning age, gender, and tumor type.

### 3.3 Clinical characteristics of the major musculoskeletal AEs

**FIGURE 5:**
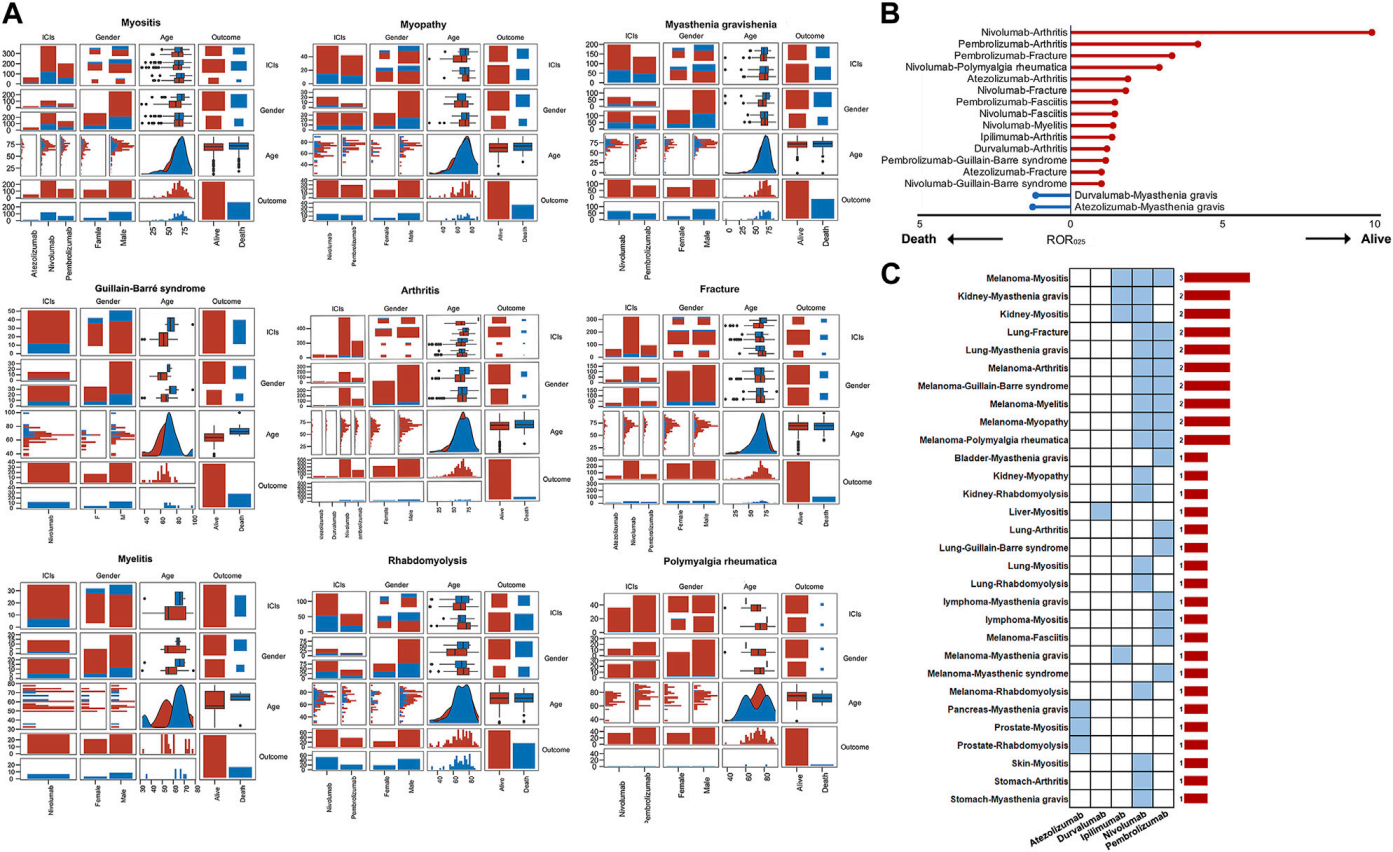
**(A)** The correlation analysis between ICIs, age, gender, and outcomes. It should be noted that some major musculoskeletal adverse events were not included in this analysis due to the smaller sample sizes (<40) with complete records of age, sex, and clinical outcomes **(B)** The results of the disproportionality analysis suggest that gender is a factor in the increased risk of death associated with certain ICIs-induced adverse events **(C)** Tumor type influences the occurrence of certain ICIs-induced adverse events. The blue cells signify statistically significant associations, while the bar chart on the right displays the number of significant associations.

Supplementary Table S4 presents baseline characteristics, including age, gender, and clinical outcomes of patients experiencing the major musculoskeletal AEs. Supplementary Table S4 and Supplementary Figure S1 depict the median onset ages of ICIs-induced myositis, myasthenia gravis, rhabdomyolysis, and fracture at approximately 70 years. The median onset ages for arthritis and chronic inflammatory demyelinating polyradiculoneuropathy are 65–70 years, and for Guillain-Barré syndrome, it is around 66 years, while for myelitis and fasciitis, it is about 60 years. Polymyalgia rheumatica and Lambert-Eaton myasthenic syndrome are more likely to occur in patients older than 70 years. The disproportionality analysis, adjusting for the initial gender imbalance among ICIs recipients, identifies a higher likelihood of myositis and rhabdomyolysis in males and a greater risk of arthritis and fractures in females (Table 3). Figure 5A displays the correlation between ICIs, age, gender, and clinical outcomes. In contrast to other musculoskeletal adverse events, the fatalities associated with fractures and polymyalgia rheumatica are infrequent among users of ICIs. In contrast, individuals with myasthenia gravis exhibit a comparatively higher incidence of mortality (Figure 5A). Our further investigation utilizing the ROR algorithm unveils that myasthenia gravis is a predictor of unfavorable clinical outcomes (fatality) in both atezolizumab (ROR_025_ = 1.25) and durvalumab (ROR_025_ = 1.15) recipients (Figure 5B). In addition, at least two types of ICIs have shown a lower risk of mortality attributed to arthritis, fractures, Guillain-Barré syndrome, and fasciitis, compared to the mortality risk of other ICIs-induced AEs (Figure 5B). Furthermore, our further analysis reveals no significant difference in mortality risk between genders for the major musculoskeletal AEs induced by ICIs (Supplementary Figure S2). However, a significant age difference is observed between patients with nivolumab-induced Guillain-Barré syndrome in the death and alive groups, with the former showing a higher age (*p* < 0.001). In contrast, the age difference between the death and alive groups for the other major musculoskeletal adverse events is not statistically significant (Supplementary Figure S3). We have also investigated whether tumor types constitute a risk factor for the incidence of adverse events in ICIs recipients. Supplementary Figures S4-S7 depict the distribution of tumor types among patients who experienced the major musculoskeletal AEs. As shown in Supplementary Figure S4 and Table 4, the risk of atezolizumab-induced myositis in patients with prostate cancer is 5.33 times greater than in patients with other tumor types (*p* = 0.001). In addition, prostate cancer and pancreatic cancer increase the risk of atezolizumab-induced rhabdomyolysis and Lambert-Eaton myasthenic syndrome, respectively. Moreover, the results indicate that patients with melanoma face an elevated risk of developing nivolumab-induced myositis, myopathy, Guillain-Barré syndrome, arthritis, rhabdomyolysis, and polymyalgia rheumatica (Supplementary Figure S5, Table 4). Notably, this risk extends beyond nivolumab, as patients with melanoma treated with pembrolizumab are also vulnerable to myositis, Lambert-Eaton myasthenic syndrome, Guillain-Barré syndrome, arthritis, myelitis, fasciitis, and polymyalgia rheumatica (Supplementary Figure S6, Table 4), while those treated with ipilimumab are also susceptible to myositis and myasthenia gravis (Supplementary Figure S7, Table 4). Furthermore, patients with kidney neoplasms are at an increased risk of developing nivolumab-induced myositis, myopathy, myasthenia gravis, and rhabdomyolysis, as well as ipilimumab-induced myositis and myasthenia gravis (Supplementary Figure S5, Supplementary Figure S7, Table 4). Interestingly, while the risk of most ICIs-induced AEs is not linked to lung cancer, it heightens the risk of fractures, a relationship consistently observed with both nivolumab and pembrolizumab (Supplementary Figure S5, Supplementary Figure S6, Table 4). The associations between certain tumor types and ICIs-induced musculoskeletal AEs, such as melanoma-myositis, kidney neoplasms-myasthenia gravis, lung cancer-fracture, lung cancer-myasthenia gravis, melanoma-arthritis, melanoma-Guillain-Barré syndrome, melanoma-myelitis, melanoma-myopathy, and melanoma-polymyalgia rheumatica, have been consistently observed across two or more types of ICIs, indicating the robustness of these associations (Figure 5C).

**TABLE 3:** The major musculoskeletal AEs with significant gender differences.

Subgroup	ICI-AE pairs	ROR_025_	
		Male vs*.* female^a^	Female vs*.* male^b^
Myositis	Nivolumab-Myositis	**1.36**	0.49
	Pembrolizumab-Myositis	**1.04**	0.57
Arthritis	Ipilimumab-Arthritis	0.13	**1.26**
Fracture	Nivolumab-Fracture	0.55	**1.24**
	Pembrolizumab-Fracture	0.47	**1.10**
Rhabdomyolysis	Ipilimumab-Rhabdomyolysis	**1.18**	0.05
	Nivolumab-Rhabdomyolysis	**1.12**	0.42
	Pembrolizumab-Rhabdomyolysis	**1.26**	0.25

^a^ When using females as the reference group, an ROR_025_ greater than 1 indicates a significantly higher proportion of males compared to females.

^b^ When using males as the reference group, an ROR_025_ greater than 1 indicates a significantly higher proportion of females compared to males.

The ROR_025_ value highlighted in bold signifies statistical significance.

**TABLE 4:** Tumor type is a factor influencing the occurrence of certain ICIs-induced adverse events.

ICIs	Subgroup	Tumor type	Or^a^	*p*-Value (χ^2^)
Atezolizumab	Myositis	Prostate	5.33	0.001
	Lambert-Eaton myasthenic syndrome	Pancreas	50.38	0.024
	Rhabdomyolysis	Prostate	10.97	0.018
Nivolumab	Myositis	Skin	2.81	0.046
		Melanoma	1.70	0.000
		Kidney	1.51	0.001
		Lung	0.69	0.001
	Myopathy	Melanoma	2.11	0.000
		Kidney	1.69	0.015
	Myasthenia gravis	Kidney	2.22	0.000
		Stomach	1.75	0.041
		Lung	0.70	0.014
	Guillain-Barré syndrome	Melanoma	3.08	0.000
	Arthritis	Melanoma	1.75	0.000
		Stomach	0.59	0.039
	Fracture	Lung	1.31	0.010
	Myelitis	Melanoma	2.17	0.001
	Rhabdomyolysis	Melanoma	1.74	0.002
		Kidney	1.15	0.002
		Lung	0.63	0.018
	Polymyalgia rheumatica	Melanoma	2.50	0.004
Pembrolizumab	Myositis	lymphoma	3.56	0.000
		Melanoma	1.36	0.039
	Myopathy	Melanoma	2.52	0.020
	Myasthenia gravis	lymphoma	3.66	0.001
		Bladder	2.03	0.004
		Lung	0.67	0.020
	Lambert-Eaton myasthenic syndrome	Melanoma	2.65	0.016
	Guillain-Barré syndrome	Melanoma	1.99	0.063
		Lung	0.42	0.029
	Arthritis	Melanoma	1.97	0.000
		Lung	0.71	0.005
	Fracture	Lung	2.31	0.000
	Myelitis	Melanoma	2.17	0.001
	Fasciitis	Melanoma	3.45	0.001
	Polymyalgia rheumatica	Melanoma	2.78	0.000
Durvalumab	Myositis	Liver	4.26	0.041
Ipilimumab	Myositis	Kidney	4.01	0.001
		Melanoma	0.31	0.004
	Myasthenia gravis	Kidney	16.56	0.000
		Melanoma	0.09	0.000

^a^ This analysis was designed as a case-control analysis. Reports were grouped into two categories based on the presence or absence of the target adverse event (such as myositis), and the odds ratio (OR) were calculated for each tumor type.

### 3.4 Results of time-to-onset analysis

**FIGURE 6:**
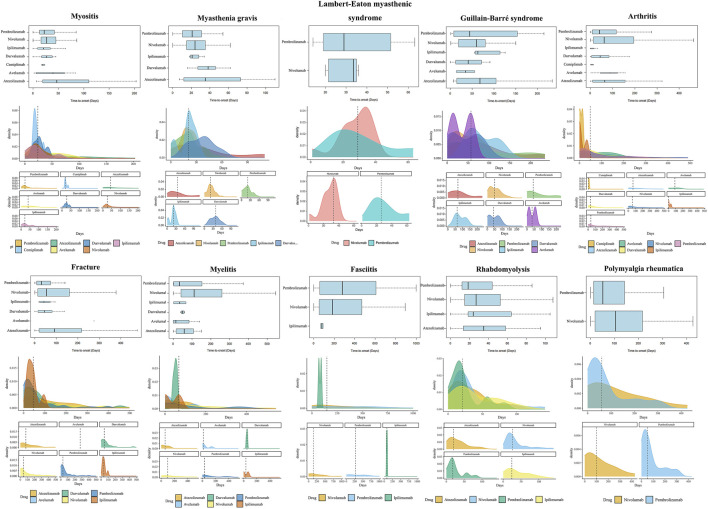
Time-to-onset analysis results. The visualization of several major ICIs-induced musculoskeletal adverse events’ time-to-onset analysis results is not displayed for their smaller sample sizes. Nonetheless, their time-to-onset analysis results are documented in Table 5.

The results of time-to-onset analysis are detailed in Table 5 and Figure 6. The preponderance of instances of ICIs-induced myositis manifests within 50 days. However, a proportion of 6.3% of nivolumab-induced myositis cases and 5.3% of pembrolizumab-induced myositis cases arise beyond 30 days after the cessation of treatment. Most cases of ICIs-induced myasthenia gravis occur within 60 days, and 12.0% of nivolumab-induced myasthenia gravis cases and 5.9% of pembrolizumab-induced myasthenia gravis cases arise beyond 30 days after treatment discontinuation. The median time to onset of ICIs-induced Guillain-Barré syndrome is expected to be in close proximity to 60 days. ICIs may induce Lambert-Eaton myasthenic syndrome with a median onset time of approximately 30 days. ICIs-induced arthritis exhibits a wide time range without a clear central tendency. Compared with atezolizumab and nivolumab-induced arthritis, with a median time of approximately 60 days, durvalumab and pembrolizumab-induced arthritis tend to occur earlier, with a median time of around 40 days. In addition, a proportion of arthritis cases occurred after treatment cessation, with 17.7% of nivolumab cases and 9.6% of pembrolizumab cases occurring 30 days after treatment. Fractures induced by ipilimumab, durvalumab, nivolumab, and pembrolizumab typically manifest within 1–16 weeks. However, a considerable proportion of fractures arise beyond 30 days post-treatment cessation: 11.6% with nivolumab and 8.1% with pembrolizumab. Notably, fractures induced by atezolizumab exhibit a longer median onset time of 92 days, and multiple cases persistently arise beyond the 16-week mark. Nivolumab-induced myelitis appears to have a more extended latency period. Nevertheless, numerous cases of myelitis have been reported after an extended period of 12 weeks following treatment initiation, irrespective of the specific type of ICI. Reports of ICIs-induced fasciitis emerge intermittently within 2 years of treatment initiation. The median time to the rhabdomyolysis onset is less than 30 days for ICIs, except for atezolizumab, which is 35 days 11.3% of nivolumab-induced rhabdomyolysis and 3.7% of pembrolizumab-induced rhabdomyolysis arises more than 30 days after treatment discontinuation. A significant number of polymyalgia rheumatica events occur after 12 weeks of treatment initiation, and the median time to onset of polymyalgia rheumatica appears to be earlier for pembrolizumab (52 days) than nivolumab (105 days). The Weibull relevant results (Table 5) suggest that arthritis and fractures tend to exhibit “early failure,” indicating that patients who develop ICIs-induced fractures or arthritis may possess pre-existing susceptibility traits. Myositis, myasthenia gravis, Lambert-Eaton myasthenic syndrome, Guillain-Barré syndrome, myelitis, and rhabdomyolysis tend to exhibit “late failure,” indicating that the risk of a patient experiencing these AEs may rise as ICIs treatment progresses. Fasciitis and rheumatoid myalgia may tend towards “random failure,” although this is not well established.

**TABLE 5:** The results of time-to-onset analysis.

Subgroup	ICIs	Cases (N)	Time-to-onset (days)		Ratio^a^	Weibull distribution	
			Range	Median		Shape β	95%CI
Myositis	Atezolizumab	55	19–110	47	-	**2.18**	**1.23–3.27**
	Avelumab	6	6–62	40	-	**-**	**-**
	Cemiplimab	5	17–23	19	-	-	-
	Durvalumab	22	18–38	27	-	**3.48**	**1.28–9.44**
	Ipilimumab	17	9–34	21	-	1.11	0.73–1.69
	Nivolumab	274	16–45	27	6.3%	**1.49**	**1.31–1.68**
	Pembrolizumab	109	13–42	22	5.3%	**1.22**	**1.03–1.44**
Myasthenia gravis	Atezolizumab	19	12–73	35	-	1.07	0.71–1.62
	Avelumab	1	-	27	-	-	-
	Cemiplimab	3	19–34	25	-	-	-
	Durvalumab	13	27–46	38	-	1.20	0.60–2.37
	Ipilimumab	15	19–28	21	-	-	-
	Nivolumab	131	15–35	24	12.0%	**1.92**	**1.49–2.46**
	Pembrolizumab	67	11–31	21	5.9%	**1.42**	**1.05–1.92**
Lambert-Eaton myasthenic syndrome	Atezolizumab	2	-	35	-	-	-
	Cemiplimab	1	-	20	-	-	-
	Durvalumab	2	48–67	58	-	-	-
	Ipilimumab	1	-	20	-	-	-
	Nivolumab	12	22–35	34	-	1.69	0.95–3.00
	Pembrolizumab	7	19–52	29	-	-	-
Guillain-Barré syndrome	Atezolizumab	16	15–104	68	-	1.12	0.75–1.66
	Avelumab	2	14–56	35	-	-	-
	Durvalumab	8	11–70	41	-	-	-
	Ipilimumab	5	56–113	63	-	-	-
	Nivolumab	30	18–79	60	-	1.33	0.97–1.82
	Pembrolizumab	16	7–152	45	-	0.81	0.54–1.21
Chronic inflammatory demyelinating polyradiculoneuropathy	Nivolumab	5	42–61	44	-	-	-
Arthritis	Atezolizumab	38	11–154	65	-	0.88	0.67–1.15
	Avelumab	2	44–123	88	-	-	-
	Cemiplimab	4	1–14	8	-	-	-
	Durvalumab	30	5–82	45	-	0.76	0.56–1.04
	Ipilimumab	3	1–16	1	-	-	-
	Nivolumab	384	14–194	62	17.7%	**0.73**	**0.67–0.80**
	Pembrolizumab	135	9–117	41	9.6%	**0.69**	**0.60–0.81**
Fracture	Atezolizumab	68	21–214	92	-	0.87	0.71–1.06
	Durvalumab	20	16–80	46	-	1.16	0.80–1.68
	Ipilimumab	8	23–71	43	-	-	-
	Nivolumab	306	14–160	55	11.6%	**0.76**	**0.69–0.83**
	Pembrolizumab	69	9–74	31	8.1%	0.95	0.77–1.17
Myelitis	Atezolizumab	12	17–104	59	-	**1.61**	**1.02–2.56**
	Avelumab	4	1–83	15	-	-	-
	Durvalumab	4	43–57	51	-	-	-
	Ipilimumab	2	2–68	35	-	-	-
	Nivolumab	30	44–252	112	-	1.06	0.79–1.41
	Pembrolizumab	10	2–153	33	-	0.83	0.49–1.42
Fasciitis	Ipilimumab	2	69–92	81		-	-
	Nivolumab	18	56–468	183	-	1.00	0.68–1.45
	Pembrolizumab	14	76–603	279	-	1.30	0.85–1.99
Rhabdomyolysis	Atezolizumab	15	14–58	35	-	**1.56**	**1.03–2.36**
	Durvalumab	5	19–42	29		-	-
	Ipilimumab	15	19–64	24	-	1.18	0.73–1.91
	Nivolumab	95	15–53	27	11.3%	**1.39**	**1.19–1.63**
	Pembrolizumab	40	12–44	19	3.7%	1.10	0.83–1.46
Polymyalgia rheumatica	Nivolumab	23	20–217	105	-	0.94	0.67–1.31
	Pembrolizumab	29	14–142	52	-	0.94	0.70–1.25

^a^ Ratio refers to the proportion of adverse events reported after a 30-day cessation of ICIs, treatment to the total number of adverse events.

The values highlighted in bold in the column “Weibull distribution” indicate statistical significance.

### 3.5 Pharmacovigilance analysis results of combination therapy

**FIGURE 7:**
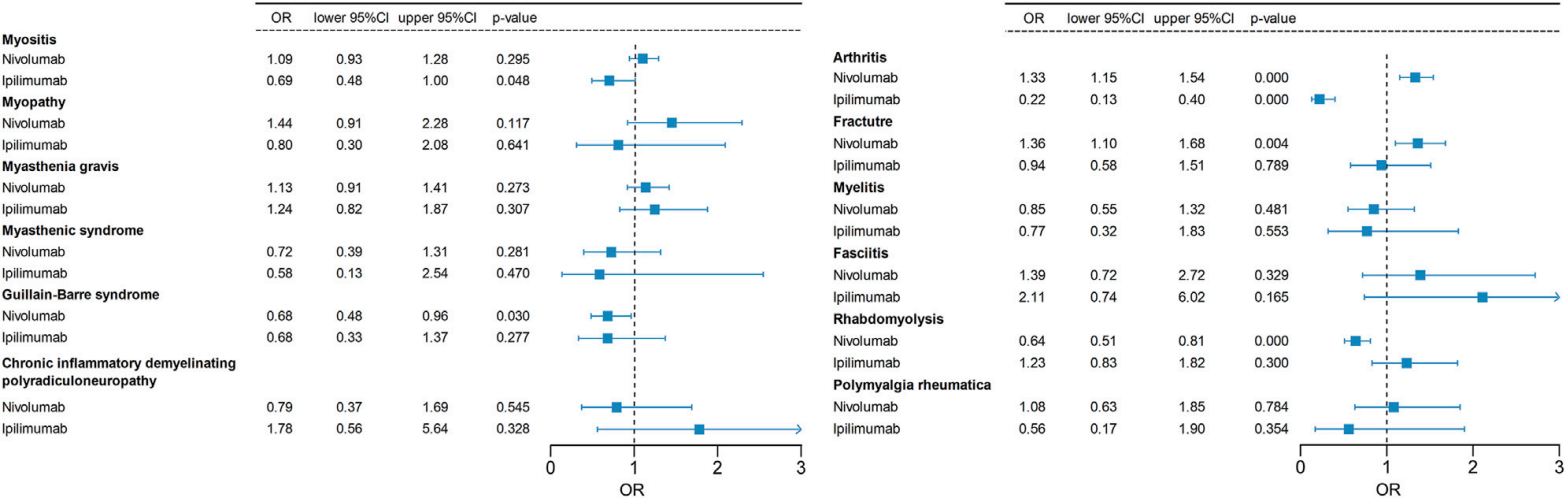
A logistic regression model incorporating age, gender, tumor type, and treatment regimen (combination therapy vs. monotherapy) is employed to examine whether the combination of nivolumab and ipilimumab increases the risk of specific adverse events. The odds ratio (OR) of nivolumab and ipilimumab monotherapy in relation to their combination therapy as the reference group is computed in this analysis.

Our study investigates the musculoskeletal adverse events reported in various combination regimens of ICIs or combined with other chemotherapeutic or targeted agents. Compared to other treatments, nivolumab plus ipilimumab is linked to a significantly greater number of musculoskeletal AEs. Hence, we conduct further analyses to evaluate the risk of musculoskeletal AEs induced by nivolumab and ipilimumab combination therapy. Logistic regression model indicates that combination therapy is an independent risk factor for Guillain-Barré syndrome and rhabdomyolysis compared to monotherapy with nivolumab. Moreover, combination therapy is an independent risk factor for myositis compared to monotherapy with ipilimumab (Figure 7; Table 6). In situations involving polypharmacy, each drug not only triggers adverse events (AEs) but also amplifies the risk of AEs due to drug-drug interactions (DDIs). We further analyzed whether there is a significantly elevated risk of the aforementioned adverse events due to interactions between nivolumab and ipilimumab. However, the Ω shrinkage measure model did not detect any significant interaction between nivolumab and ipilimumab concerning the aforementioned adverse events, suggesting that the increased risk of combination therapy observed in logistic regression model, compared to individual use of nivolumab or ipilimumab, might be attributed to the additive effects of the drugs rather than an interaction between the two. The fact that the increased risk associated with drug combinations originates from drug co-administration rather than DDIs also suggests a certain level of safety and manageability in drug co-administration. It is important to note, however, that weak drug-drug interaction signals were detected for Guillain-Barré syndrome, myasthenic syndrome, arthritis, and polymyalgia rheumatica, although they did not reach statistical significance.

**TABLE 6:** The analysis results of logistic regression and DDIs for the combination therapy of nivolumab and ipilimumab.

Subgroup	ICI regimens	Logistic regression model			Obs (N)^a^	Ω shrinkage measure model			
		OR	95%CI	*p*-Value		Exp^b^	Ω	*Ω*_025_ ^c^	*Ω*_095_
Myositis	Nivolumab	1.09	0.93–1.28	0.295					
	Ipilimumab	0.69	0.48–1.00	0.048					
	Combine therapy	ref			276	286.05	−0.05	−0.53	0.43
Guillain-Barre syndrome	Nivolumab	0.68	0.48–0.96	0.030					
	Ipilimumab	0.68	0.33–1.37	0.277					
	Combine therapy	ref			73	72.96	0.00	−0.63	0.63
Myasthenia gravis	Nivolumab	1.13	0.91–1.41	0.273					
	Ipilimumab	1.24	0.82–1.87	0.307					
	Combine therapy	ref			155	233.27	−0.59	−1.13	−0.05
Rhabdomyolysis	Nivolumab	0.64	0.51–0.81	<0.001					
	Ipilimumab	1.23	0.83–1.82	0.300					
	Combine therapy	ref			139	154.52	−0.15	−0.70	0.40
Chronic inflammatory demyelinating polyradiculoneuropathy	Nivolumab	0.79	0.37–1.69	0.545					
	Ipilimumab	1.78	0.56–5.64	0.328					
	Combine therapy	ref			13	28.17	−1.09	−2.15	−0.03
Arthritis	Nivolumab	1.33	1.15–1.54	<0.001					
	Ipilimumab	0.22	0.13–0.40	<0.001					
	Combine therapy	ref			360	326.38	0.14	−0.32	0.60
Fracture	Nivolumab	1.36	1.10–1.68	0.004					
	Ipilimumab	0.94	0.58–1.51	0.789					
	Combine therapy				136	334.48	−1.30	−1.85	−0.74
Myelitis	Nivolumab	0.85	0.55–1.32	0.481					
	Ipilimumab	0.77	0.32–1.83	0.553					
	Combine therapy	ref			47	68.25	−0.53	−1.24	0.17
Polymyalgia rheumatica	Nivolumab	1.08	0.63–1.85	0.784					
	Ipilimumab	0.56	0.17–1.90	0.354					
	Combine therapy	ref			27	18.34	0.55	−0.28	1.37
Fasciitis	Nivolumab	1.39	0.72–2.72	0.329					
	Ipilimumab	2.11	0.74–6.02	0.165					
	Combine therapy	ref			17	26.94	−0.65	−1.61	0.31
Myopathy	Nivolumab	1.44	0.91–2.28	0.117					
	Ipilimumab	0.80	0.30–2.08	0.641					
	Combine therapy	ref			37	37.44	−0.02	−0.77	0.74
Myasthenic syndrome	Nivolumab	0.72	0.39–1.31	0.281					
	Ipilimumab	0.58	0.13–2.54	0.470					
	Combine therapy	ref			22	19.86	0.14	−0.74	1.02

^a^ Obs refers to the actual observed count of adverse events induced by the combined use of nivolumab and ipilimumab.

^b^ Exp denotes the expected count of adverse events induced by the combined use of nivolumab and ipilimumab, calculated using the Ω Shrinkage Measure Model.

^c^ *Ω*_025_ > 0 is used as a threshold for detecting the DDIs.

## 4 Discussion

Immune checkpoint inhibitor is a highly promising cancer immunotherapy strategy, but managing associated adverse effects is equally challenging (Bylsma et al., 2022). A meta-analysis of 36 phases II/III trials estimated the safety of ICIs, with all adverse event incidences ranging from 54% to 76% (Xu et al., 2018). Our study demonstrates a more pronounced musculoskeletal toxicity observed for PD-1 inhibitor pembrolizumab, and the relatively weaker musculoskeletal toxicity observed for CTLA-4 inhibitor Ipilimumab (Figure 2). The toxicity profile of CTLA-4 inhibitors and PD-1/PD-L1 inhibitors differ (Xu et al., 2018; Ramos-Casals et al., 2020). According to a prior study, the most commonly observed irAEs associated with CTLA-4 inhibitors are dermatological, gastrointestinal, and renal toxicities compared to PD-1/PD-L1 inhibitors (Xu et al., 2018). Our study further substantiates that CTLA-4 inhibitor (ipilimumab) poses a higher risk for inducing endocrine diseases, skin and subcutaneous tissue disorders, and gastrointestinal disorders, and a relatively lower risk for inducing cardiac, hematologic, and lymphatic diseases compared to PD-1/PD-L1 inhibitors (Figure 2). Actually, the precise biological explanations for the localization and severity of AEs induced by different ICIs remain unclear. However, current evidence suggests that PD-1/PD-L1 inhibitors have a more tolerable toxicity profile than CTLA-4 inhibitors (Khoja et al., 2017).

According to previous studies, ICI-induced arthritis has been observed in around 5% of patients treated with ICIs, exhibiting varying degrees of severity (Weinmann and pisetsky, 2019; Gatto et al., 2022). Some studies have suggested a higher incidence of arthritis with pembrolizumab (Xu et al., 2018; Ramos-Casals et al., 2020). Our study further highlights the substantial risk of pembrolizumab-induced arthritis, whereas the risks associated with cemiplimab and avelumab have not been detected and require further consideration. ICI-induced arthritis is commonly known as inflammatory arthritis (IA) (Reid and cappelli, 2022). However, apart from inflammatory arthritis, ICIs-induced non-inflammatory arthritis (such as osteoarthritis and ankylosis), as well as crystalline arthritis (gouty arthritis), has also been collected in our study (Supplementary Table S1, Supplementary Table S2). Therefore, it is imperative to consider them as potential differential diagnoses to distinguish from ICIs-induced inflammatory arthritis (Cappelli and bingham, 2021). A significant number of ICIs-induced rheumatoid arthritis reports are collected in this study. In actuality, up to 11% of patients with established ICI-induced arthritis may have detectable RF and/or CCP autoantibodies (Ghosh et al., 2022). There is some evidence that treating CCP seropositive asymptomatic patients with ICIs may accelerate the onset of ICI-induced arthritis (Belkhir et al., 2017; Hommes et al., 2020). We are considering is that the administration of ICIs can exacerbate pre-existing immune-related diseases, leading to flare-ups, and patients with conditions such as osteoarthritis, psoriatic arthritis, and ankylosing spondylitis may experience a recurrence or exacerbation of symptoms following ICI treatment (Richter et al., 2018; Halle et al., 2021; Reid et al., 2021; Schütz and baraliakos, 2023). Our large-scale data survey collects 76 reports of osteoarthritis (including 31 cases of spondyloarthritis), 95 reports of psoriatic arthritis, and 7 reports of ankylosing spondylitis. Notably, our analysis reveales that the risk of ICIs-induced psoriatic arthritis is relatively higher than that of induced osteoarthritis and ankylosing spondylitis (Supplementary Table S1). Therefore, in clinical practice, the clinical benefits of ICI treatment in patients with those pre-existing conditions must be balanced against the risk of disease reactivation, especially in patients with a history of psoriasis, as ICIs seem to have a high propensity to reactivate the disease. Osteoarthritis and spinal fracture are common causes of spinal stenosis (Moller et al., 2007; Byvaltsev et al., 2022). ICIs may heighten the risk of spinal stenosis in patients, and the occurrence of fracture events and inflammation induction around the vertebrae due to ICIs may explain it, as our study finds that some patients with spinal stenosis also experience the presence of concurrent spinal fractures and osteoarthritis, albeit in a smaller proportion. Additionally, it is worth noting that there is a significant correlation between the administration of nivolumab and pembrolizumab and the development of hypertrophic osteoarthropathy and gouty arthritis (Supplementary Table S1). Nevertheless, the relationship has not been adequately characterized in previous studies. Some evidence indicates an overlap in susceptibility between ICIs-induced irAEs and conventional autoimmune diseases (Wu et al., 2021). Our study indicates that the female gender is a notable risk factor for developing ICI-induced arthritis (Table 3). In a prior multicenter clinical study, melanoma and genitourinary tumors were found to be risk factors for rheumatic irAEs induced by ICIs, as compared to lung cancer (Cunningham-Bussel et al., 2022). Our study further confirms that melanoma increases the risk of ICIs-induced arthritis. However, genitourinary tumors fail to show statistically significant differences in the incidence of ICIs-induced arthritis compared to the pan-cancer background (Table 4). Moreover, thyroid dysfunction, predominantly hypothyroidism, as well as colitis and diarrhea (terms that appear to describe inflammatory bowel disease), are prominent comorbidities of ICIs-induced arthritis (Table 1). Patients with thyroid dysfunction and inflammatory bowel disease can develop joint symptoms, and there is a biological link between the pathogenesis of these conditions and that of arthritis (Shukla et al., 2021; Wu et al., 2021; Ohta et al., 2023). Furthermore, a study indicates that the association between thyroid dysfunction and arthritis may be bidirectional in nature, and effective treatment and management may confer mutual benefits (Wu et al., 2021). Given the significant risk of ICIs-induced hypothyroidism and inflammatory bowel disease (Xu et al., 2018; Ramos-Casals et al., 2020), the thyroid and gut toxicities of ICIs may contribute to the pathogenesis of ICI-induced arthritis. This underscores the importance of special attention to diagnosing and treating thyroid dysfunction and inflammatory bowel disease in ICIs recipients with arthritis in clinical practice.

Our study confirms that all ICIs carry the risk of inducing myositis, with atezolizumab and pembrolizumab being particularly noteworthy (Supplementary Table S1, Figure 3). Previous research has suggested that myositis induced by ICIs can be complicated by myocarditis (Nakagomi et al., 2022; Pinal-Fernandez et al., 2023). Our study provides further insight into the relationship between myositis and myocarditis. Specifically, our findings reveal that myocarditis occurs concomitantly in 54%, 36%, and 20% of cases with ICIs-induced autoimmune myositis, immune-mediated myositis, and polymyositis, respectively. It is important to note, however, that our study does not support the notion that myocarditis is a common comorbidity of ICIs-induced dermatomyositis (Table 1). Indeed, myositis induced by ICIs with concomitant myocarditis may represent a specific subtype characterized by markedly inflammatory muscle biopsies and activation of the type 2 interferon pathway, while the features of ICIs-induced dermatomyositis are the presence of anti-TIF1γ autoantibodies and overexpression of type 1 interferon-inducible genes (Pinal-Fernandez et al., 2023). Immune-mediated necrotizing myositis is characterized by predominant necrotizing pathology and low levels of muscle inflammation in patients (Pinal-Fernandez et al., 2023), and our study indicates that the most significant comorbidity of ICIs-induced immune-mediated necrotizing myositis is rhabdomyolysis (Table 1). ICI-induced dermatomyositis often co-occurs with pneumonia or interstitial lung disease (Table 1), and the underlying link for this association may be due to the ICIs-induced anti-synthetase syndrome (Opinc and makowska, 2021). Our study reveals an association between nivolumab and the onset of anti-synthetase syndrome (ROR_025_ = 2.77, IC_025_ = 0.44, Supplementary Table S1). However, the link between ICIs and anti-synthetase syndrome may be underestimated since we find patients with concurrent dermatomyositis and interstitial lung disease reported as separate cases of dermatomyositis or interstitial lung disease, rather than being described as anti-synthetase syndrome. Pulmonary disease is a major cause of death among patients with inflammatory myopathies. The manifestation of concomitant pulmonary disease in these patients can vary from asymptomatic to rapidly progressive respiratory failure, which can mimic acute respiratory distress syndrome (ARDS). Unfortunately, the latter is often misdiagnosed during the initial stages (Hallowell and danoff, 2023). Our study indicates that approximately 14% of patients with ICIs-induced myositis may develop respiratory dysfunction (Table 1), providing insight for managing potential pulmonary complications in patients with ICIs-induced myositis.

Previous literature has reported fracture as a potential risk associated with ICIs treatment. It has been found that the incidence of fractures increases after the initiation of ICIs treatment (Filippini et al., 2021; Ye et al., 2023). However, the characteristics of ICIs-induced fractures remain unclear. Our study indicates that the overall risk of fracture induced by ICIs is insignificant. Nevertheless, several notable pharmacovigilance signals exist for ICIs-induced osteoporotic-type fractures, and the risk of ICIs-induced spinal fracture should be given attention (Figure 4). Our study uncovers the critical role of osteoporosis in ICIs-induced fractures (Figure 4). However, it remains unclear whether the osteoporosis observed in these fracture patients is attributed to using ICIs. Based on the available evidence, we suggest that ICIs use partially contributes to osteoporosis. Previous studies suggest that ICIs may accelerate bone resorption (Moseley et al., 2018). Several studies suggest that ICIs trigger the production of pro-inflammatory cytokines by T-cell and upregulate the nuclear factor-κB ligand, promoting the differentiation and maturation of osteoclasts over osteoblastogenesis, which may contribute to the development of bone loss associated with ICIs (Tang, 2023). Our study also collects reports of ICIs-induced bone resorption and osteolysis (Supplementary Table S1). Furthermore, atezolizumab poses the highest risk of inducing osteoporosis rather than fall and is also associated with the most significant incidence of fracture events (Figure 4). This correlation supports the possibility that ICIs-induced fractures may result from ICIs-induced osteoporosis. Nevertheless, it is essential to consider that osteoporosis in recipients of ICIs may also be attributable to the effects of other drugs used in the comprehensive treatment of cancer, as the pharmacovigilance analysis indicates the risk of ICIs-induced bone resorption or osteoporosis is relatively low compared to other medications (Supplementary Table S1). It is conceivable that females could be more prone to ICIs-related fractures, given that certain indications point to a potential overlap in susceptibility between pre-existing conditions and adverse events (Wu et al., 2021). Our study has confirmed this notion, showing that females exhibit a higher propensity for ICIs-induced fractures. Notably, nearly one-third of patients with ICIs-induced fractures had a history of falls (Table 1). Although the risks of ICI-induced fall require further evaluation, we can hypothesize that fall is a significant contributing factor to ICIs-induced fractures. In addition, atezolizumab carries the highest risk of fracture-related mortality among all ICIs, and the median time to fracture occurrence is the longest, with events still occurring up to 16 weeks. We speculate that atezolizumab therapy may potentially elicit long-term toxic effects on bone density, leading to successive fracture events with poorer clinical outcomes. However, an alternative explanation for the higher mortality risk of fractures associated with atezolizumab may be due to its relatively milder toxicity (Ramos-Casals et al., 2020), which could lead to a lower baseline risk of death and consequently amplify the mortality risk of fractures. The closer association between atezolizumab and fractures compared to other ICIs warrants further investigation. Additionally, there appears to be a higher incidence of fracture events in lung cancer patients, although the exact underlying mechanisms remain unclear.

ICIs exhibit significant neuro-muscular junction toxicity (Hyun et al., 2022; Zhang et al., 2022). Our study further elucidates that this toxicity is primarily manifested in the induction of myasthenia gravis, Lambert-Eaton myasthenic syndrome, Guillain-Barré syndrome (Supplementary Table S1). In addition, chronic inflammatory demyelinating polyneuropathy is another adverse event of neuromyopathy induced by ICIs. Furthermore, prior research has identified pembrolizumab as having the highest risk of inducing neuromuscular junction disease (Zhang et al., 2022). Nonetheless, our study uncovers the strongest association between pembrolizumab and myasthenia gravis and Lambert-Eaton myasthenic syndrome. However, Guillain-Barré syndrome and chronic inflammatory demyelinating polyneuropathy are more closely linked to ipilimumab. Guillain-Barré syndrome and chronic inflammatory demyelinating polyneuropathy are conditions associated with nerve demyelination changes (Pollard, 2002; Rimkus et al., 2021). This suggests a potentially high level of neurotoxicity of ipilimumab in terms of inducing nerve demyelination. Among patients with myasthenia gravis, approximately 38% of them have concurrent myositis, which is lower than the two-thirds estimated in a previous study (Huang et al., 2020). Moreover, 27% of patients have concurrent myocarditis, which is higher than the 13% previously reported (Johansen et al., 2019) and is consistent with the 31% estimated in another study (Huang et al., 2020). Patients with Lambert-Eaton myasthenic syndrome have a lower occurrence of concurrent myositis and myocarditis than those with myasthenia gravis, with 19% and 16% rates, respectively (Table 1).

Our study demonstrates a strong correlation between ICIs and Sjogren’s syndrome, particularly with avelumab (Supplementary Table S1). This intriguing observation is noteworthy since avelumab is not commonly associated with pronounced musculoskeletal toxicity (Supplementary Table S1, Figure 3), yet it exhibits a strong association with Sjogren’s syndrome. Moreover, some pathological evidence suggests that the adverse event known as “Sjogren’s syndrome” induced by ICIs may be distinct from the typical Sjogren’s syndrome. It typically occurs abruptly within the first 3 months of treatment and is associated with glandular inflammation and damage (Warner et al., 2019).

The results of Weibull distribution fitting for fractures and arthritis show an “early failure” type (Table 5), and the occurrence of these adverse events persists over a long period after the initiation of ICIs (Figure 6). This intriguing phenomenon may reflect the stratification of patients with ICIs-induced arthritis and fractures into two distinct groups. The first group is more susceptible to these conditions, exhibiting toxicities soon after receiving ICIs treatment. The second group, however, may not exhibit susceptibility initially but may develop arthritis or fractures after prolonged toxic accumulation. As a result, ICIs-induced arthritis and fractures exhibit a rapid onset with a subsequent decrease in the number of cases (characteristic of early failure), followed by a continuous occurrence of cases over an extended period (characteristic of long-term toxicity accumulation). In addition, our study reveals susceptibility characteristics of arthritis and fractures in terms of gender and tumor types (Table 3, Table 4). These findings suggest that females with melanoma receiving ICIs should remain vigilant for early signs of arthritis, while males may need to monitor joint symptoms for a prolonged period. Females with lung cancer receiving ICIs should also be aware of the risk of early-stage fractures, while other recipients should consider long-term monitoring of their bone density.

Our study has some limitations to be considered when interpreting our findings. Firstly, the accuracy of our SOC classification is reduced due to vague descriptions of some adverse events, which are symptoms rather than specific diseases. Additionally, the risk of some adverse events induced by ICIs may be underestimated due to interference from powerful background signals that are difficult to evaluate. Third, some data, such as age and gender, are missing from the adverse event reports collected in the FAERS database, potentially leading to bias in our analysis results. Finally, in line with FAERS recommendation, we substitute “01″for any missing day in the date, which means that some medical details are approximated, which could also introduce bias into our analysis.

## 5 Conclusion

The administration of immune checkpoint inhibitors (ICIs) can result in various musculoskeletal adverse events. The major musculoskeletal adverse events linked to ICIs include myositis, neuromyopathy (including myasthenia gravis, Lambert-Eaton myasthenic syndrome, Guillain-Barré syndrome, and Chronic inflammatory demyelinating polyradiculoneuropathy), arthritis, fractures, myelitis, spinal stenosis, Sjogren’s syndrome, fasciitis, tenosynovitis, rhabdomyolysis, rheumatoid myalgia, and chondrocalcinosis. The overall risk of musculoskeletal adverse events is most prominent with pembrolizumab. However, certain musculoskeletal adverse events, such as neurological demyelination and rhabdomyolysis, are most strongly associated with ipilimumab. In contrast, myositis and fractures are most strongly associated with atezolizumab, and Sjogren’s syndrome with avelumab. Musculoskeletal adverse events frequently have complications, and their monitoring for complications should be a priority in clinical practice. The risk of ICIs-induced musculoskeletal adverse events is affected by gender and tumor type, and monitoring of adverse events should be focused differently based on these factors. ICIs may also contribute to osteoporosis and falls, playing a crucial role in ICIs-induced fractures. The analysis based on the Ω shrinkage measure model indicate that the combination therapy of nivolumab and ipilimumab does not result in a statistically significant escalation of the risk associated with the major musculoskeletal adverse events.

## Data Availability

The original contributions presented in the study are included in the article/Supplementary Material, further inquiries can be directed to the corresponding authors.
