# Supplementary material for: Musculoskeletal adverse events induced by immune checkpoint inhibitors: a large-scale pharmacovigilance study
Source: Front Pharmacol. 2023 Oct 10;14:1199031. doi: 10.3389/fphar.2023.1199031 (PMC10595016; doi:10.3389/fphar.2023.1199031)
Supplement: Supplementary file 1 [file Table1.DOCX]

Supplementary Table S1. Singals of ICIs-induced musculoskeletal AEs.

| **Cemiplimab** | **Subgroups** | **AEs** | **Cases (N)** | **Signals ^a^** | |
| --- | --- | --- | --- | --- | --- |
|  |  |  |  | **ROR (ROR_025_)** | **IC (IC_025_)** |
| Atezolizumab | Joint | **non-specific arthritis** | 66 | **2.64 (2.07)** | **1.36 (1.01)** |
|  |  | **rheumatoid arthritis** | 15 | **2.09 (1.25)** | **1.00 (0.26)** |
|  |  | **autoimmune arthritis** | 11 | **19.64 (10.46)** | **3.34 (2.46)** |
|  |  | immune-mediated arthritis | 2 | **4.98 (1.22)** | 1.45 (-0.39) |
|  |  | osteoarthritis | 7 | 0.85 (0.40) | -0.22 (-1.28) |
|  |  | seronegative arthritis | 1 | 2.45 (0.34) | 0.72 (-1.65) |
|  |  | oligoarthritis | 1 | **13.14 (1.70)** | 1.36 (-1.01) |
|  |  | periarthritis | 1 | 1.72 (0.24) | 0.47 (-1.90) |
|  |  | peripheral arthritis | 1 | **24.09 (2.90)** | 1.45 (-0.93) |
|  |  | **polyarthritis** | 10 | **3.64 (1.94)** | **1.67 (0.77)** |
|  |  | non-specific arthropathy | 3 | 0.18 (0.06) | -2.28 (-3.82) |
|  |  | psoriatic arthropathy | 2 | 1.45 (0.36) | 0.41 (-1.42) |
|  |  | neuropathic arthropathy | 1 | 6.02 (0.81) | 1.16 (-1.21) |
|  |  | arthralgia | 103 | 0.66 (0.54) | -0.59 (-0.88) |
|  | Muscle | **non-specific myositis** | 102 | **11.01 (9.00)** | **3.30 (3.00)** |
|  |  | **autoimmune myositis** | 4 | **20.65 (7.24)** | **2.64 (1.26)** |
|  |  | **immune-mediated myositis** | 4 | **8.26 (3.02)** | **2.16 (0.78)** |
|  |  | **dermatomyositis** | 12 | **7.14 (4.00)** | **2.47 (1.64)** |
|  |  | **polymyositis** | 13 | **12.53 (7.11)** | **3.06 (2.25)** |
|  |  | **rhabdomyolysis** | 29 | **2.96 (2.05)** | **1.50 (0.96)** |
|  |  | **non-specific myopathy** | 10 | **2.72 (1.46)** | **1.32 (0.42)** |
|  |  | muscle necrosis | 1 | 2.33 (0.32) | 0.69 (-1.68) |
|  |  | **polymyalgia rheumatica** | 9 | **4.66 (2.40)** | **1.94 (0.99)** |
|  |  | **myasthenia gravis** | 55 | **10.41 (7.92)** | **3.18 (2.78)** |
|  |  | **Lambert-Eaton myasthenic syndrome** | 8 | **10.15 (4.95)** | **2.67 (1.66)** |
|  |  | **muscular weakness** | 126 | **2.32 (1.95)** | **1.19 (0.93)** |
|  |  | muscle spasms | 19 | 0.19 (0.12) | -2.34 (-2.99) |
|  |  | muscle atrophy | 3 | 0.66 (0.21) | -0.53 (-2.07) |
|  |  | **Guillain-Barré syndrome** | 32 | **8.01 (5.61)** | **2.79 (2.27)** |
|  |  | chronic inflammatory demyelinating polyradiculoneuropathy | 1 | 1.10 (0.15) | 0.09 (-2.28) |
|  | Sjogren's syndrome | **Sjogren's syndrome** | 8 | **3.70 (1.83)** | **1.65 (0.65)** |
|  | Fasciitis | **eosinophilic fasciitis** | 3 | **8.18 (2.56)** | **1.98 (0.42)** |
|  | Tenosynovitis | **tenosynovitis** | 6 | **9.75 (4.26)** | **2.50 (1.34)** |
|  | Tendonitis | **tendonitis** | 7 | **2.49 (1.18)** | **1.17 (0.11)** |
|  | Fall | fall | 69 | 0.52 (0.41) | -0.93 (-1.27) |
|  | Fracture | **non-specific fracture** | 16 | **2.09 (1.28)** | **1.01 (0.29)** |
|  |  | hip fracture | 14 | 0.82 (0.49) | -0.27 (-1.03) |
|  |  | **spinal compression fracture** | 13 | **1.89 (1.09)** | **0.86 (0.07)** |
|  |  | spinal fracture | 12 | 1.14 (0.64) | 0.18 (-0.64) |
|  |  | lumbar vertebral fracture | 4 | 1.59 (0.59) | 0.57 (-0.79) |
|  |  | thoracic vertebral fracture | 1 | 0.61 (0.09) | -0.51 (-2.88) |
|  |  | **pathological fracture** | 11 | **2.10 (1.16)** | **1.00 (0.14)** |
|  |  | femur fracture | 9 | 0.93 (0.48) | -0.09 (-1.03) |
|  |  | ankle fracture | 5 | 1.43 (0.59) | 0.46 (-0.78) |
|  |  | femoral neck fracture | 5 | 1.73 (0.71) | 0.69 (-0.55) |
|  |  | foot fracture | 3 | 0.70 (0.22) | -0.46 (-2.00) |
|  |  | rib fracture | 3 | 0.28 (0.09) | -1.67 (-3.21) |
|  |  | hand fracture | 2 | 1.30 (0.32) | 0.29 (-1.54) |
|  |  | pelvic fracture | 2 | 0.63 (0.16) | -0.56 (-2.38) |
|  |  | clavicle fracture | 1 | 0.58 (0.08) | -0.57 (-2.93) |
|  |  | facial bones fracture | 1 | 0.60 (0.08) | -0.52 (-2.88) |
|  |  | fracture displacement | 1 | **10.33 (1.36)** | 1.31 (-1.06) |
|  |  | humerus fracture | 1 | 0.37 (0.05) | -1.09 (-3.45) |
|  |  | ilium fracture | 1 | 5.16 (0.70) | 1.10 (-1.27) |
|  |  | lower limb fracture | 1 | 0.16 (0.02) | -2.19 (-4.55) |
|  |  | scapula fracture | 1 | 4.38 (0.60) | 1.03 (-1.34) |
|  |  | tibia fracture | 1 | 1.14 (0.16) | 0.12 (-2.24) |
|  | Spine | **spinal cord compression** | 13 | **3.14 (1.81)** | **1.52 (0.73)** |
|  |  | lumbar spinal stenosis | 2 | 2.33 (0.58) | 0.87 (-0.96) |
|  |  | **non-specific myelitis** | 11 | **9.04 (4.91)** | **2.69 (1.82)** |
|  |  | **myelitis transverse** | 7 | **9.46 (4.40)** | **2.55 (1.47)** |
|  |  | encephalomyelitis | 2 | **9.04 (2.17)** | 1.77 (-0.08) |
|  | Bone/cartilage | bone lesion | 2 | 0.36 (0.09) | -1.26 (-3.08) |
|  |  | osteonecrosis | 8 | 0.98 (0.49) | -0.02 (-1.02) |
|  |  | osteonecrosis of jaw | 3 | 0.08 (0.03) | -3.37 (-4.91) |
|  |  | osteitis deformans | 1 | 5.35 (0.73) | 1.12 (-1.26) |
|  |  | osteosclerosis | 1 | 1.12 (0.16) | 0.11 (-2.26) |
|  |  | chondrocalcinosis | 2 | **7.41 (1.79)** | 1.68 (-0.17) |
| Avelumab | Joint | non-specific arthritis | 4 | 2.47 (0.93) | 1.09 (-0.28) |
|  |  | rheumatoid arthritis | 1 | 2.21 (0.31) | 0.65 (-1.70) |
|  |  | non-specific arthropathy | 1 | 0.95 (0.13) | -0.05 (-2.41) |
|  |  | arthralgia | 3 | 0.30 (0.10) | -1.57 (-3.11) |
|  | Muscle | **non-specific myositis** | 6 | **10.15 (4.55)** | **2.57 (1.44)** |
|  |  | **rhabdomyolysis** | 3 | **4.65 (1.50)** | **1.61 (0.06)** |
|  |  | immune-mediated myositis | 1 | **34.68 (4.82)** | 1.50 (-0.85) |
|  |  | non-specific myopathy | 2 | **8.37 (2.09)** | 1.76 (-0.07) |
|  |  | **myasthenia gravis** | 4 | **12.10 (4.53)** | **2.43 (1.07)** |
|  |  | muscular weakness | 4 | 1.11 (0.42) | 0.14 (-1.22) |
|  |  | muscle spasms | 2 | 0.32 (0.08) | -1.44 (-3.26) |
|  |  | **polymyalgia rheumatica** | 2 | **16.94 (4.21)** | **2.01 (0.19)** |
|  |  | Guillain-Barré syndrome | 2 | **7.32 (1.83)** | 1.69 (-0.14) |
|  | Sjogren's syndrome | **Sjogren's syndrome** | 7 | **50.80 (24.01)** | **3.55 (2.49)** |
|  | Fasciitis | fasciitis | 1 | **41.51 (5.75)** | 1.52 (-0.84) |
|  | Fall | fall | 6 | 0.70 (0.32) | -0.47 (-1.60) |
|  | Fracture | femur fracture | 1 | 1.53 (0.22) | 0.38 (-1.98) |
|  | Spine | non-specific myelitis | 1 | **12.35 (1.73)** | 1.37 (-0.99) |
|  |  | spinal cord compression | 1 | 3.35 (0.47) | 0.91 (-1.45) |
|  | Bone/cartilage | osteoporosis | 1 | 1.58 (0.22) | 0.41 (-1.95) |
| Cemiplimab | Joint | non-specific arthritis | 3 | 2.47 (0.93) | 1.21 (-0.33) |
|  |  | rheumatoid arthritis | 1 | 3.32 (0.47) | 0.90 (-1.45) |
|  |  | seronegative arthritis | 1 | **53.43 (7.36)** | 1.53 (-0.83) |
|  |  | polyarthritis | 1 | **8.40 (1.18)** | 1.28 (-1.08) |
|  |  | arthralgia | 9 | 1.60 (0.83) | 0.63 (-0.31) |
|  | Muscle | **non-specific myositis** | 6 | **14.08 (6.30)** | **2.80 (1.67)** |
|  |  | **autoimmune myositis** | 2 | **167.56 (39.99)** | **2.29 (0.46)** |
|  |  | **myasthenia gravis** | 5 | **18.95 (7.84)** | **2.84 (1.61)** |
|  |  | Lambert-Eaton myasthenic syndrome | 1 | **26.16 (3.64)** | 1.48 (-0.88) |
|  |  | muscular weakness | 1 | 0.52 (0.07) | -0.69 (-3.05) |
|  |  | muscle spasms | 1 | 0.30 (0.04) | -1.36 (-3.72) |
|  |  | musculoskeletal pain | 2 | 1.86 (0.47) | 0.67 (-1.16) |
|  |  | musculoskeletal stiffness | 2 | 2.69 (0.67) | 1.01 (-0.82) |
|  | Fall | fall | 6 | 1.28 (0.57) | 0.32 (-0.81) |
|  | Fracture | rib fracture | 1 | 2.64 (0.37) | 0.77 (-1.59) |
|  | Bone/cartilage | chondrocalcinosis pyrophosphate | 1 | **30.62 (4.26)** | 1.49 (-0.86) |
| Durvalumab | Joint | **non-specific arthritis** | 23 | **2.09 (1.38)** | **1.02 (0.42)** |
|  |  | **rheumatoid arthritis** | 12 | **3.67 (2.08)** | **1.71 (0.89)** |
|  |  | **autoimmune arthritis** | 3 | **9.89 (3.11)** | **2.10 (0.55)** |
|  |  | immune-mediated arthritis | 1 | 3.86 (0.53) | 0.98 (-1.39) |
|  |  | seronegative arthritis | 1 | 4.84 (0.67) | 1.08 (-1.29) |
|  |  | **polyarthritis** | 5 | **3.86 (1.59)** | **1.60 (0.36)** |
|  |  | osteoarthritis | 1 | 0.29 (0.04) | -1.40 (-3.76) |
|  |  | spinal osteoarthritis | 1 | 0.89 (0.13) | -0.11 (-2.47) |
|  |  | arthritis infective | 2 | 2.46 (0.61) | 0.92 (-0.91) |
|  |  | arthritis bacterial | 1 | 1.52 (0.21) | 0.37 (-1.99) |
|  |  | ankylosing spondylitis | 1 | **9.10 (1.23)** | 1.29 (-1.08) |
|  |  | non-specific arthropathy | 4 | 0.58 (0.22) | -0.72 (-2.08) |
|  |  | psoriatic arthropathy | 1 | 1.56 (0.22) | 0.39 (-1.97) |
|  |  | arthralgia | 57 | 0.92 (0.71) | -0.12 (-0.50) |
|  | Muscle | **non-specific myositis** | 25 | **5.40 (3.63)** | **2.29 (1.71)** |
|  |  | autoimmune myositis | 1 | **7.34 (1.00)** | 1.23 (-1.14) |
|  |  | immune-mediated myositis | 1 | 3.12 (0.43) | 0.86 (-1.50) |
|  |  | **polymyositis** | 6 | **12.65 (5.56)** | **2.70 (1.55)** |
|  |  | dermatomyositis | 2 | 2.38 (0.59) | 0.89 (-0.94) |
|  |  | necrotising myositis | 2 | **10.34 (2.51)** | 1.83 (-0.01) |
|  |  | non-specific myopathy | 3 | 1.89 (0.61) | 0.74 (-0.81) |
|  |  | **neuromyopathy** | 2 | **14.22 (3.41)** | **1.95 (0.11)** |
|  |  | **myasthenia gravis** | 26 | **9.20 (6.22)** | **2.95 (2.38)** |
|  |  | myasthenia gravis crisis | 2 | **8.75 (2.13)** | 1.76 (-0.07) |
|  |  | **Lambert-Eaton myasthenic syndrome** | 5 | **12.37 (5.03)** | **2.57 (1.33)** |
|  |  | **muscular weakness** | 35 | **1.67 (1.20)** | **0.72 (0.23)** |
|  |  | muscle spasms | 10 | 0.27 (0.15) | -1.83 (-2.72) |
|  |  | **polymyalgia rheumatica** | 6 | **6.10 (2.71)** | **2.11 (0.97)** |
|  |  | rhabdomyolysis | 8 | 1.93 (0.96) | 0.87 (-0.13) |
|  |  | **Guillain-Barré syndrome** | 10 | **5.74 (3.06)** | **2.20 (1.30)** |
|  |  | chronic inflammatory demyelinating polyradiculoneuropathy | 2 | **4.89 (1.21)** | 1.45 (-0.39) |
|  | Sjogren's syndrome | Sjogren's syndrome | 3 | 2.68 (0.86) | 1.10 (-0.45) |
|  | Fall | fall | 22 | 0.42 (0.28) | -1.21 (-1.82) |
|  | Fracture | compression fracture | 4 | **3.07 (1.14)** | 1.31 (-0.06) |
|  |  | spinal compression fracture | 2 | 0.71 (0.18) | -0.41 (-2.24) |
|  |  | spinal fracture | 2 | 0.48 (0.12) | -0.90 (-2.72) |
|  |  | fibula fracture | 1 | 3.45 (0.48) | 0.92 (-1.45) |
|  |  | upper limb fracture | 3 | 0.90 (0.29) | -0.13 (-1.68) |
|  |  | rib fracture | 2 | 0.48 (0.12) | -0.90 (-2.73) |
|  |  | pathological fracture | 2 | 1.02 (0.26) | 0.03 (-1.80) |
|  |  | radius fracture | 2 | **6.15 (1.51)** | 1.59 (-0.25) |
|  |  | femur fracture | 2 | 0.53 (0.13) | -0.77 (-2.59) |
|  |  | wrist fracture | 1 | 0.92 (0.13) | -0.08 (-2.44) |
|  |  | pelvic fracture | 1 | 0.77 (0.11) | -0.26 (-2.62) |
|  |  | sternal fracture | 1 | 3.92 (0.54) | 0.98 (-1.38) |
|  | Spine | **non-specific myelitis** | 3 | **4.98 (1.59)** | **1.65 (0.10)** |
|  |  | **myelitis transverse** | 3 | **7.85 (2.48)** | **1.97 (0.41)** |
|  |  | encephalomyelitis | 1 | **10.34 (1.39)** | 1.32 (-1.05) |
|  |  | Spinal stenosis | 1 | 1.64 (0.23) | 0.43 (-1.93) |
|  | Bone/cartilage | bone lesion | 1 | 0.43 (0.06) | -0.91 (-3.27) |
|  |  | osteoporosis | 2 | 0.56 (0.14) | -0.71 (-2.54) |
|  |  | osteolysis | 1 | 0.96 (0.14) | -0.04 (-2.40) |
|  |  | osteonecrosis | 1 | 0.30 (0.04) | -1.35 (-3.71) |
|  |  | chondrocalcinosis pyrophosphate | 1 | 2.77 (0.39) | 0.80 (-1.57) |
| Ipilimumab | Joint | non-specific arthritis | 22 | 1.44 (0.94) | 0.51 (-0.10) |
|  |  | **rheumatoid arthritis** | 13 | **3.06 (1.77)** | **1.49 (0.71)** |
|  |  | polyarthritis | 4 | 2.49 (0.93) | 1.09 (-0.28) |
|  |  | systemic lupus erythematosus (SLE) arthritis | 2 | **3.11 (1.00)** | 1.25 (-0.30) |
|  |  | osteoarthritis | 1 | 0.17 (0.02) | -2.11 (-4.47) |
|  |  | non-specific arthropathy | 8 | 0.80 (0.40) | -0.31 (-1.30) |
|  |  | psoriatic arthropathy | 3 | **3.80 (1.22)** | 1.43 (-0.12) |
|  |  | arthralgia | 65 | 0.69 (0.54) | -0.52 (-0.88) |
|  | Muscle | **non-specific myositis** | 33 | **5.97 (4.23)** | **2.45 (1.94)** |
|  |  | **dermatomyositis** | 7 | **6.93 (3.27)** | **2.29 (1.23)** |
|  |  | **non-specific myopathy** | 6 | **2.66 (1.19)** | **1.23 (0.09)** |
|  |  | **muscular weakness** | 52 | **1.53 (1.17)** | **0.60 (0.20)** |
|  |  | **myasthenia Gravis** | 38 | **12.60 (9.10)** | **3.40 (2.92)** |
|  |  | **Lambert-Eaton myasthenic syndrome** | 3 | **6.44 (2.05)** | **1.84 (0.29)** |
|  |  | **rhabdomyolysis** | 36 | **5.99 (4.30)** | **2.46 (1.98)** |
|  |  | **Guillain-Barré syndrome** | 18 | **7.09 (4.44)** | **2.58 (1.90)** |
|  |  | **chronic inflammatory demyelinating polyradiculoneuropathy** | 7 | **14.34 (6.70)** | **2.89 (1.82)** |
|  |  | muscle spasms | 21 | 0.35 (0.23) | -1.48 (-2.10) |
|  | Sjogren's syndrome | **Sjogren's syndrome** | 8 | **6.10 (3.03)** | **2.21 (1.21)** |
|  | Fasciitis | necrotising fasciitis | 3 | 2.03 (0.65) | 0.82 (-0.73) |
|  |  | fasciitis | 1 | 4.36 (0.60) | 1.03 (-1.33) |
|  |  | plantar fasciitis | 1 | 1.74 (0.24) | 0.48 (-1.88) |
|  | Fall | fall | 38 | 0.47 (0.34) | -1.07 (-1.54) |
|  | Fracture | spinal compression fracture | 5 | 1.13 (0.47) | 0.15 (-1.08) |
|  |  | compression fracture | 3 | 1.20 (0.39) | 0.22 (-1.32) |
|  |  | lumbar vertebral fracture | 1 | 0.62 (0.09) | -0.49 (-2.85) |
|  |  | pathological fracture | 5 | 1.32 (0.55) | 0.36 (-0.88) |
|  |  | non-specific fracture | 4 | 0.83 (0.31) | -0.25 (-1.61) |
|  |  | upper limb fracture | 4 | 0.79 (0.29) | -0.31 (-1.68) |
|  |  | clavicle fracture | 2 | 1.79 (0.44) | 0.62 (-1.21) |
|  |  | femur fracture | 2 | 0.32 (0.08) | -1.42 (-3.24) |
|  |  | spinal fracture | 2 | 0.31 (0.08) | -1.47 (-3.30) |
|  |  | ankle fracture | 1 | 0.46 (0.06) | -0.83 (-3.19) |
|  |  | hip fracture | 1 | 0.09 (0.01) | -2.89 (-5.25) |
|  |  | lower limb fracture | 1 | 0.26 (0.04) | -1.54 (-3.90) |
|  | Spine | **non-specific myelitis** | 12 | **16.47 (9.19)** | **3.30 (2.47)** |
|  |  | **myelitis transverse** | 3 | **6.39 (2.03)** | **1.84 (0.29)** |
|  |  | noninfectious myelitis | 1 | **29.57 (3.75)** | 1.48 (-0.89) |
|  |  | **spinal shock** | 4 | **266.22 (66.57)** | **3.09 (1.70)** |
|  | Bone/cartilage | bone density decreased | 2 | 1.78 (0.44) | 0.62 (-1.21) |
|  |  | osteonecrosis | 2 | 0.36 (0.09) | -1.29 (-3.11) |
|  |  | osteoporosis | 2 | 0.33 (0.08) | -1.37 (-3.20) |
| Nivolumab | Joint | **non-specific arthritis** | 500 | **3.36 (3.06)** | **1.61 (1.48)** |
|  |  | **rheumatoid arthritis** | 303 | **8.70 (7.63)** | **2.72 (2.54)** |
|  |  | **autoimmune arthritis** | 38 | **17.02 (11.24)** | **3.21 (2.67)** |
|  |  | **immune-mediated arthritis** | 11 | **5.43 (2.82)** | **2.00 (1.08)** |
|  |  | **seronegative arthritis** | 28 | **19.34 (11.77)** | **3.25 (2.61)** |
|  |  | osteoarthritis | 27 | 0.41 (0.28) | -1.22 (-1.78) |
|  |  | spinal osteoarthritis | 14 | 0.52 (0.30) | -0.90 (-1.67) |
|  |  | **gouty arthritis** | 13 | **3.83 (2.14)** | **1.66 (0.83)** |
|  |  | **periarthritis** | 11 | **2.71 (1.46)** | **1.25 (0.36)** |
|  |  | peripheral arthritis | 1 | 4.03 (0.49) | 0.95 (-1.50) |
|  |  | **oligoarthritis** | 6 | **24.18 (7.80)** | **2.73 (1.46)** |
|  |  | arthritis bacterial | 8 | 0.71 (0.35) | -0.46 (-1.46) |
|  |  | arthritis infective | 7 | 0.55 (0.26) | -0.78 (-1.85) |
|  |  | arthritis reactive | 4 | 2.10 (0.76) | 0.86 (-0.55) |
|  |  | **polyarthritis** | 188 | **18.65 (15.41)** | **3.42 (3.17)** |
|  |  | monarthritis | 1 | 1.73 (0.23) | 0.45 (-1.97) |
|  |  | **ankylosing spondylitis** | 5 | **3.78 (1.47)** | **1.48 (0.18)** |
|  |  | non-specific arthropathy | 70 | 0.65 (0.51) | -0.60 (-0.95) |
|  |  | **psoriatic arthropathy** | 64 | **10.39 (7.75)** | **2.85 (2.44)** |
|  |  | **hypertrophic osteoarthropathy** | 4 | **16.12 (4.55)** | **2.33 (0.84)** |
|  |  | spondyloarthropathy | 2 | **6.91 (1.43)** | 1.54 (-0.38) |
|  |  | neuropathic arthropathy | 1 | 0.73 (0.10) | -0.30 (-2.70) |
|  |  | temporomandibular joint syndrome | 3 | 0.46 (0.15) | -0.98 (-2.54) |
|  |  | **arthralgia** | 1208 | **1.23 (1.16)** | **0.28 (0.20)** |
|  | Muscle | **non-specific myositis** | 523 | **12.90 (11.60)** | **3.12 (2.97)** |
|  |  | **immune-mediated myositis** | 19 | **8.35 (4.96)** | **2.50 (1.78)** |
|  |  | **autoimmune myositis** | 12 | **14.51 (7.09)** | **2.82 (1.90)** |
|  |  | **polymyositis** | 64 | **12.79 (9.45)** | **3.04 (2.63)** |
|  |  | **dermatomyositis** | 63 | **7.15 (5.40)** | **2.47 (2.07)** |
|  |  | **necrotising myositis** | 16 | **9.21 (5.18)** | **2.56 (1.77)** |
|  |  | **orbital myositis** | 7 | **10.58 (4.35)** | **2.41 (1.25)** |
|  |  | **inclusion body myositis** | 4 | **24.18 (6.05)** | **2.46 (0.96)** |
|  |  | infective myositis | 1 | 1.15 (0.15) | 0.13 (-2.28) |
|  |  | **non-specific myopathy** | 86 | **3.99 (3.18)** | **1.81 (1.48)** |
|  |  | immune-mediated necrotising myopathy | 2 | 2.20 (0.52) | 0.78 (-1.10) |
|  |  | **neuromyopathy** | 9 | **4.63 (2.27)** | **1.80 (0.80)** |
|  |  | myopathy toxic | 2 | 1.51 (0.36) | 0.43 (-1.44) |
|  |  | **myasthenia gravis** | 405 | **22.45 (19.61)** | **3.58 (3.40)** |
|  |  | **myasthenia gravis crisis** | 16 | **7.74 (4.41)** | **2.40 (1.62)** |
|  |  | **Lambert-Eaton myasthenic syndrome** | 49 | **15.19 (10.63)** | **3.16 (2.68)** |
|  |  | **muscular weakness** | 482 | **1.35 (1.24)** | **0.42 (0.28)** |
|  |  | neuralgic amyotrophy | 3 | **3.45 (1.03)** | 1.27 (-0.35) |
|  |  | muscle spasms | 191 | 0.29 (0.25) | -1.72 (-1.93) |
|  |  | **polymyalgia rheumatica** | 57 | **5.65 (4.23)** | **2.21 (1.79)** |
|  |  | **rhabdomyolysis** | 246 | **4.27 (3.73)** | **1.91 (1.71)** |
|  |  | **Guillain-Barré syndrome** | 110 | **4.56 (3.72)** | **1.98 (1.68)** |
|  |  | **chronic inflammatory demyelinating polyradiculoneuropathy** | 22 | **4.63 (2.93)** | **1.92 (1.27)** |
|  | Sjogren's syndrome | **Sjogren's syndrome** | 104 | **9.94 (7.91)** | **2.83 (2.51)** |
|  | Anti-synthetase syndrome | **anti-synthetase syndrome** | 5 | **7.56 (2.77)** | **2.04 (0.72)** |
|  | Fasciitis | **eosinophilic fasciitis** | 36 | **41.45 (24.20)** | **3.72 (3.13)** |
|  |  | **fasciitis** | 11 | **5.21 (2.72)** | **1.96 (1.04)** |
|  |  | necrotising fasciitis | 11 | 0.69 (0.38) | -0.50 (-1.36) |
|  |  | **myofascitis** | 5 | **13.43 (4.50)** | **2.38 (1.03)** |
|  | Tenosynovitis | **tenosynovitis** | 11 | **2.29 (1.24)** | **1.05 (0.17)** |
|  | Tendonitis | tendonitis | 8 | 0.39 (0.20) | -1.26 (-2.26) |
|  | Fall | fall | 616 | 0.71 (0.66) | -0.47 (-0.59) |
|  | Fracture | non-specific fracture | 61 | 1.20 (0.92) | 0.24 (-0.13) |
|  |  | femur fracture | 71 | 1.09 (0.86) | 0.11 (-0.23) |
|  |  | hip fracture | 71 | 0.62 (0.49) | -0.67 (-1.01) |
|  |  | rib fracture | 50 | 0.68 (0.51) | -0.54 (-0.95) |
|  |  | pathological fracture | 49 | 1.23 (0.92) | 0.28 (-0.14) |
|  |  | **femoral neck fracture** | 34 | **1.83 (1.29)** | **0.81 (0.30)** |
|  |  | spinal fracture | 31 | 0.44 (0.31) | -1.13 (-1.65) |
|  |  | spinal compression fracture | 30 | 0.63 (0.44) | -0.64 (-1.17) |
|  |  | thoracic vertebral fracture | 7 | 0.62 (0.29) | -0.62 (-1.69) |
|  |  | cervical vertebral fracture | 8 | 0.76 (0.38) | -0.35 (-1.36) |
|  |  | humerus fracture | 27 | **1.48 (1.00)** | 0.53 (-0.04) |
|  |  | compression fracture | 26 | 0.98 (0.66) | -0.03 (-0.60) |
|  |  | lumbar vertebral fracture | 24 | 1.43 (0.95) | 0.48 (-0.12) |
|  |  | ankle fracture | 20 | 0.86 (0.55) | -0.20 (-0.85) |
|  |  | lower limb fracture | 17 | 0.41 (0.25) | -1.24 (-1.94) |
|  |  | clavicle fracture | 15 | 1.27 (0.76) | 0.32 (-0.43) |
|  |  | upper limb fracture | 12 | 0.22 (0.12) | -2.12 (-2.94) |
|  |  | **osteoporotic fracture** | 10 | **2.81 (1.46)** | **1.28 (0.35)** |
|  |  | foot fracture | 9 | 0.30 (0.15) | -1.66 (-2.60) |
|  |  | wrist fracture | 9 | 0.49 (0.25) | -0.96 (-1.91) |
|  |  | pubis fracture | 7 | 2.01 (0.93) | 0.87 (-0.22) |
|  |  | facial bones fracture | 6 | 0.52 (0.23) | -0.87 (-2.02) |
|  |  | multiple fracture | 6 | 0.56 (0.25) | -0.76 (-1.91) |
|  |  | tibia fracture | 6 | 0.96 (0.42) | -0.05 (-1.20) |
|  |  | acetabulum fracture | 5 | **2.75 (1.09)** | 1.17 (-0.12) |
|  |  | **forearm fracture** | 5 | **3.78 (1.47)** | **1.48 (0.18)** |
|  |  | fractured sacrum | 5 | 1.26 (0.51) | 0.29 (-0.97) |
|  |  | hand fracture | 5 | 0.47 (0.20) | -0.98 (-2.22) |
|  |  | patella fracture | 5 | 1.12 (0.46) | 0.14 (-1.12) |
|  |  | scapula fracture | 4 | 2.69 (0.96) | 1.11 (-0.31) |
|  |  | **skull fractured base** | 4 | **5.69 (1.91)** | **1.75 (0.31)** |
|  |  | traumatic fracture | 4 | 1.05 (0.39) | 0.06 (-1.33) |
|  |  | skull fracture | 1 | 0.29 (0.04) | -1.34 (-3.71) |
|  |  | jaw fracture | 3 | 0.37 (0.12) | -1.27 (-2.83) |
|  |  | radius fracture | 3 | 0.50 (0.16) | -0.87 (-2.43) |
|  |  | sternal fracture | 3 | 0.59 (0.19) | -0.65 (-2.22) |
|  |  | fibula fracture | 2 | 0.45 (0.11) | -0.95 (-2.79) |
|  |  | fractured coccyx | 2 | 1.18 (0.29) | 0.18 (-1.69) |
|  |  | fractured ischium | 2 | **6.04 (1.28)** | 1.48 (-0.44) |
|  |  | ilium fracture | 2 | 1.61 (0.39) | 0.50 (-1.38) |
|  |  | pelvic fracture | 2 | 0.09 (0.02) | -3.09 (-4.92) |
|  |  | stress fracture | 2 | 0.21 (0.05) | -1.95 (-3.78) |
|  |  | fracture treatment | 1 | **24.18 (1.51)** | 1.37 (-1.10) |
|  |  | ulna fracture | 1 | 0.81 (0.11) | -0.21 (-2.60) |
|  | Spine | **non-specific myelitis** | 41 | **6.01 (4.27)** | **2.26 (1.77)** |
|  |  | **myelitis transverse** | 16 | **3.45 (2.05)** | **1.56 (0.81)** |
|  |  | **encephalomyelitis** | 12 | **10.75 (5.44)** | **2.61 (1.70)** |
|  |  | **neuromyelitis optica spectrum disorder** | 7 | **15.39 (5.96)** | **2.63 (1.45)** |
|  |  | acute disseminated encephalomyelitis | 2 | 4.40 (0.97) | 1.30 (-0.61) |
|  |  | **spinal cord compression** | 47 | **1.60 (1.19)** | **0.64 (0.20)** |
|  |  | **spinal stenosis** | 15 | **2.90 (1.70)** | **1.35 (0.59)** |
|  |  | spinal column stenosis | 7 | 0.46 (0.22) | -1.02 (-2.09) |
|  |  | cervical spinal stenosis | 3 | 0.70 (0.22) | -0.43 (-1.99) |
|  |  | lumbar spinal stenosis | 1 | 0.12 (0.02) | -2.48 (-4.84) |
|  |  | spinal cord injury | 2 | 0.50 (0.12) | -0.81 (-2.66) |
|  |  | spinal ligament ossification | 2 | **9.67 (1.88)** | 1.68 (-0.25) |
|  |  | spinal shock | 2 | **8.06 (1.63)** | 1.61 (-0.32) |
|  | Bone/cartilage | bone lesion | 21 | 0.49 (0.32) | -0.98 (-1.61) |
|  |  | osteitis | 7 | 0.52 (0.25) | -0.86 (-1.92) |
|  |  | osteochondrosis | 2 | 0.78 (0.19) | -0.28 (-2.14) |
|  |  | **chondritis** | 10 | **17.27 (7.67)** | **2.85 (1.84)** |
|  |  | chondropathy | 1 | 0.27 (0.04) | -1.46 (-3.83) |
|  |  | osteonecrosis | 36 | 0.60 (0.43) | -0.72 (-1.20) |
|  |  | osteoradionecrosis | 4 | 1.34 (0.49) | 0.35 (-1.04) |
|  |  | osteonecrosis of jaw | 44 | 0.16 (0.12) | -2.60 (-3.04) |
|  |  | osteoporosis | 24 | 0.37 (0.25) | -1.38 (-1.97) |
|  |  | osteolysis | 10 | 0.43 (0.23) | -1.15 (-2.05) |
|  |  | resorption bone increased | 2 | 2.69 (0.62) | 0.95 (-0.94) |
|  |  | bone density decreased | 2 | 0.16 (0.04) | -2.32 (-4.15) |
|  |  | osteitis deformans | 1 | 0.56 (0.08) | -0.58 (-2.97) |
|  |  | **Chondrocalcinosis pyrophosphate** | 18 | **3.13 (1.92)** | **1.46 (0.75)** |
|  |  | chondrocalcinosis | 1 | 0.48 (0.07) | -0.75 (-3.14) |
| Pembrolizumab | Joint | **non-specific arthritis** | 124 | **1.86 (1.55)** | **0.87 (0.60)** |
|  |  | **immune-mediated arthritis** | 44 | **146.91 (82.89)** | **4.79 (4.26)** |
|  |  | **autoimmune arthritis** | 33 | **29.88 (19.51)** | **3.93 (3.37)** |
|  |  | **seronegative arthritis** | 13 | **14.77 (7.99)** | **3.07 (2.23)** |
|  |  | **polyarthritis** | 48 | **7.14 (5.28)** | **2.60 (2.17)** |
|  |  | **oligoarthritis** | 4 | **26.70 (8.04)** | **2.64 (1.21)** |
|  |  | **rheumatoid arthritis** | 133 | **7.72 (6.44)** | **2.75 (2.48)** |
|  |  | gouty arthritis | 2 | 1.57 (0.38) | 0.48 (-1.36) |
|  |  | **spinal osteoarthritis** | 16 | **2.35 (1.43)** | **1.14 (0.42)** |
|  |  | osteoarthritis | 9 | 0.40 (0.21) | -1.25 (-2.19) |
|  |  | arthritis bacterial | 3 | 0.66 (0.21) | -0.51 (-2.06) |
|  |  | arthritis infective | 3 | 0.59 (0.19) | -0.67 (-2.22) |
|  |  | arthritis reactive | 1 | 1.67 (0.23) | 0.44 (-1.95) |
|  |  | ankylosing spondylitis | 2 | 3.95 (0.94) | 1.27 (-0.59) |
|  |  | non-specific arthropathy | 19 | 0.43 (0.27) | -1.19 (-1.85) |
|  |  | **psoriatic arthropathy** | 25 | **7.59 (4.99)** | **2.60 (2.00)** |
|  |  | temporomandibular joint syndrome | 3 | 1.46 (0.46) | 0.44 (-1.12) |
|  |  | **arthralgia** | 476 | **1.15 (1.05)** | **0.20 (0.06)** |
|  | Muscle | **non-specific myositis** | 235 | **10.40 (9.04)** | **3.12 (2.92)** |
|  |  | **immune-mediated myositis** | 43 | **74.10 (46.69)** | **4.55 (4.02)** |
|  |  | **autoimmune myositis** | 6 | **12.32 (5.07)** | **2.58 (1.39)** |
|  |  | **dermatomyositis** | 22 | **5.04 (3.26)** | **2.12 (1.48)** |
|  |  | **polymyositis** | 18 | **6.63 (4.06)** | **2.40 (1.70)** |
|  |  | **necrotising myositis** | 8 | **8.54 (4.05)** | **2.44 (1.40)** |
|  |  | **inclusion body myositis** | 3 | **32.04 (7.66)** | **2.44 (0.82)** |
|  |  | **myositis ossificans** | 2 | **106.78 (9.68)** | **2.17 (0.27)** |
|  |  | orbital myositis | 2 | **5.08 (1.19)** | 1.44 (-0.43) |
|  |  | infective myositis | 1 | 3.81 (0.50) | 0.95 (-1.45) |
|  |  | **non-specific myopathy** | 41 | **4.38 (3.19)** | **1.99 (1.53)** |
|  |  | **immune-mediated necrotising myopathy** | 12 | **58.25 (25.70)** | **3.76 (2.85)** |
|  |  | **neuromyopathy** | 11 | **16.32 (8.31)** | **3.08 (2.16)** |
|  |  | **muscular weakness** | 217 | **1.49 (1.30)** | **0.56 (0.36)** |
|  |  | neuralgic amyotrophy | 3 | 2.43 (0.33) | 0.70 (-1.69) |
|  |  | muscle spasms | 67 | 0.25 (0.20) | -1.96 (-2.32) |
|  |  | **myasthenia gravis** | 215 | **19.02 (16.28)** | **3.79 (3.57)** |
|  |  | **myasthenia gravis crisis** | 16 | **17.44 (9.92)** | **3.28 (2.51)** |
|  |  | **Lambert-Eaton myasthenic syndrome** | 33 | **19.80 (13.28)** | **3.61 (3.06)** |
|  |  | **polymyalgia rheumatica** | 108 | **32.08 (25.27)** | **4.23 (3.91)** |
|  |  | **rhabdomyolysis** | 84 | **3.30 (2.65)** | **1.64 (1.32)** |
|  |  | **Guillain-Barré syndrome** | 45 | **4.25 (3.14)** | **1.96 (1.51)** |
|  |  | **chronic inflammatory demyelinating polyradiculoneuropathy** | 8 | **3.42 (1.67)** | **1.53 (0.51)** |
|  | Sjogren's syndrome | **Sjogren's syndrome** | 70 | **14.90 (11.43)** | **3.46 (3.08)** |
|  | Fasciitis | **eosinophilic fasciitis** | 15 | **19.54 (10.81)** | **3.34 (2.54)** |
|  |  | **fasciitis** | 5 | **5.45 (2.17)** | **1.88 (0.61)** |
|  |  | necrotising fasciitis | 8 | 1.42 (0.71) | 0.46 (-0.54) |
|  | Fall | fall | 134 | 0.37 (0.32) | -1.39 (-1.64) |
|  | Tenosynovitis | **tenosynovitis** | 10 | **6.28 (3.26)** | **2.22 (1.30)** |
|  | Tendonitis | tendonitis | 6 | 0.79 (0.35) | -0.32 (-1.46) |
|  | Fracture | non-specific fracture | 3 | 0.14 (0.05) | -2.59 (-4.14) |
|  |  | spinal fracture | 9 | 0.31 (0.16) | -1.60 (-2.54) |
|  |  | **spinal compression fracture** | 33 | **1.81 (1.28)** | **0.82 (0.31)** |
|  |  | cervical vertebral fracture | 4 | 0.98 (0.37) | -0.02 (-1.39) |
|  |  | **thoracic vertebral fracture** | 13 | **3.07 (1.76)** | **1.46 (0.66)** |
|  |  | lumbar vertebral fracture | 11 | 1.65 (0.91) | 0.67 (-0.20) |
|  |  | femur fracture | 13 | 0.50 (0.29) | -0.96 (-1.75) |
|  |  | hip fracture | 12 | 0.26 (0.15) | -1.88 (-2.70) |
|  |  | pelvic fracture | 11 | 1.30 (0.72) | 0.35 (-0.51) |
|  |  | rib fracture | 10 | 0.35 (0.19) | -1.46 (-2.35) |
|  |  | femoral neck fracture | 8 | 1.03 (0.51) | 0.04 (-0.96) |
|  |  | **osteoporotic fracture** | 8 | **5.15 (2.49)** | **1.97 (0.94)** |
|  |  | facial bones fracture | 7 | 1.60 (0.75) | 0.61 (-0.46) |
|  |  | compression fracture | 6 | 0.62 (0.28) | -0.64 (-1.78) |
|  |  | pathological fracture | 6 | 0.42 (0.19) | -1.17 (-2.30) |
|  |  | clavicle fracture | 4 | 0.86 (0.32) | -0.19 (-1.56) |
|  |  | stress fracture | 3 | 0.87 (0.28) | -0.17 (-1.73) |
|  |  | fractured ischium | 2 | **13.35 (2.83)** | 1.87 (-0.02) |
|  |  | lower limb fracture | 2 | 0.12 (0.03) | -2.80 (-4.63) |
|  |  | multiple fracture | 2 | 0.49 (0.12) | -0.87 (-2.70) |
|  |  | pubis fracture | 2 | 1.44 (0.35) | 0.40 (-1.45) |
|  |  | ankle fracture | 1 | 0.10 (0.01) | -2.72 (-5.08) |
|  |  | foot fracture | 1 | 0.09 (0.01) | -3.00 (-5.36) |
|  |  | fracture displacement | 1 | 3.81 (0.50) | 0.95 (-1.45) |
|  |  | fractured sacrum | 1 | 0.63 (0.09) | -0.47 (-2.84) |
|  |  | humerus fracture | 1 | 0.14 (0.02) | -2.36 (-4.72) |
|  |  | limb fracture | 1 | **13.35 (1.49)** | 1.34 (-1.07) |
|  |  | open fracture | 1 | 2.67 (0.36) | 0.76 (-1.63) |
|  |  | tooth fracture | 1 | 0.09 (0.01) | -2.86 (-5.22) |
|  |  | upper limb fracture | 1 | 0.04 (0.01) | -3.92 (-6.27) |
|  |  | wrist fracture | 1 | 0.14 (0.02) | -2.36 (-4.72) |
|  | Spine | **non-specific myelitis** | 8 | **2.39 (1.18)** | **1.11 (0.10)** |
|  |  | **myelitis transverse** | 8 | **4.03 (1.96)** | **1.71 (0.69)** |
|  |  | acute disseminated encephalomyelitis | 2 | **13.35 (2.83)** | 1.87 (-0.02) |
|  |  | neuromyelitis optica spectrum disorder | 2 | **6.67 (1.53)** | 1.59 (-0.29) |
|  |  | spinal cord compression | 7 | 0.62 (0.29) | -0.65 (-1.71) |
|  |  | cervical spinal stenosis | 1 | 0.71 (0.10) | -0.34 (-2.71) |
|  |  | **lumbar spinal stenosis** | 7 | **3.14 (1.47)** | **1.41 (0.33)** |
|  |  | spinal column stenosis | 3 | 0.65 (0.21) | -0.53 (-2.08) |
|  |  | spinal cord injury | 1 | 0.66 (0.09) | -0.42 (-2.79) |
|  | Bone/cartilage | bone lesion | 9 | 0.61 (0.32) | -0.67 (-1.62) |
|  |  | bone loss | 2 | 0.62 (0.15) | -0.57 (-2.41) |
|  |  | bone density abnormal | 1 | 0.92 (0.13) | -0.08 (-2.46) |
|  |  | bone density decreased | 1 | 0.20 (0.03) | -1.85 (-4.21) |
|  |  | osteoporosis | 13 | 0.54 (0.32) | -0.84 (-1.63) |
|  |  | osteolysis | 5 | 0.71 (0.29) | -0.45 (-1.69) |
|  |  | osteopenia | 3 | 0.39 (0.13) | -1.20 (-2.74) |
|  |  | osteomalacia | 2 | **5.93 (1.38)** | 1.53 (-0.35) |
|  |  | osteochondrosis | 2 | 2.18 (0.53) | 0.80 (-1.06) |
|  |  | chondropathy | 1 | 0.75 (0.10) | -0.28 (-2.66) |
|  |  | osteonecrosis | 9 | 0.41 (0.21) | -1.23 (-2.17) |

^a^Signals indicated in bold are statistically significant.
