# Supplementary material for: Musculoskeletal adverse events induced by immune checkpoint inhibitors: a large-scale pharmacovigilance study
Source: Front Pharmacol. 2023 Oct 10;14:1199031. doi: 10.3389/fphar.2023.1199031 (PMC10595016; doi:10.3389/fphar.2023.1199031)
Supplement: Supplementary file 2 [file DataSheet1.zip › Supplementary Figures/Supplementary Figures.pdf]

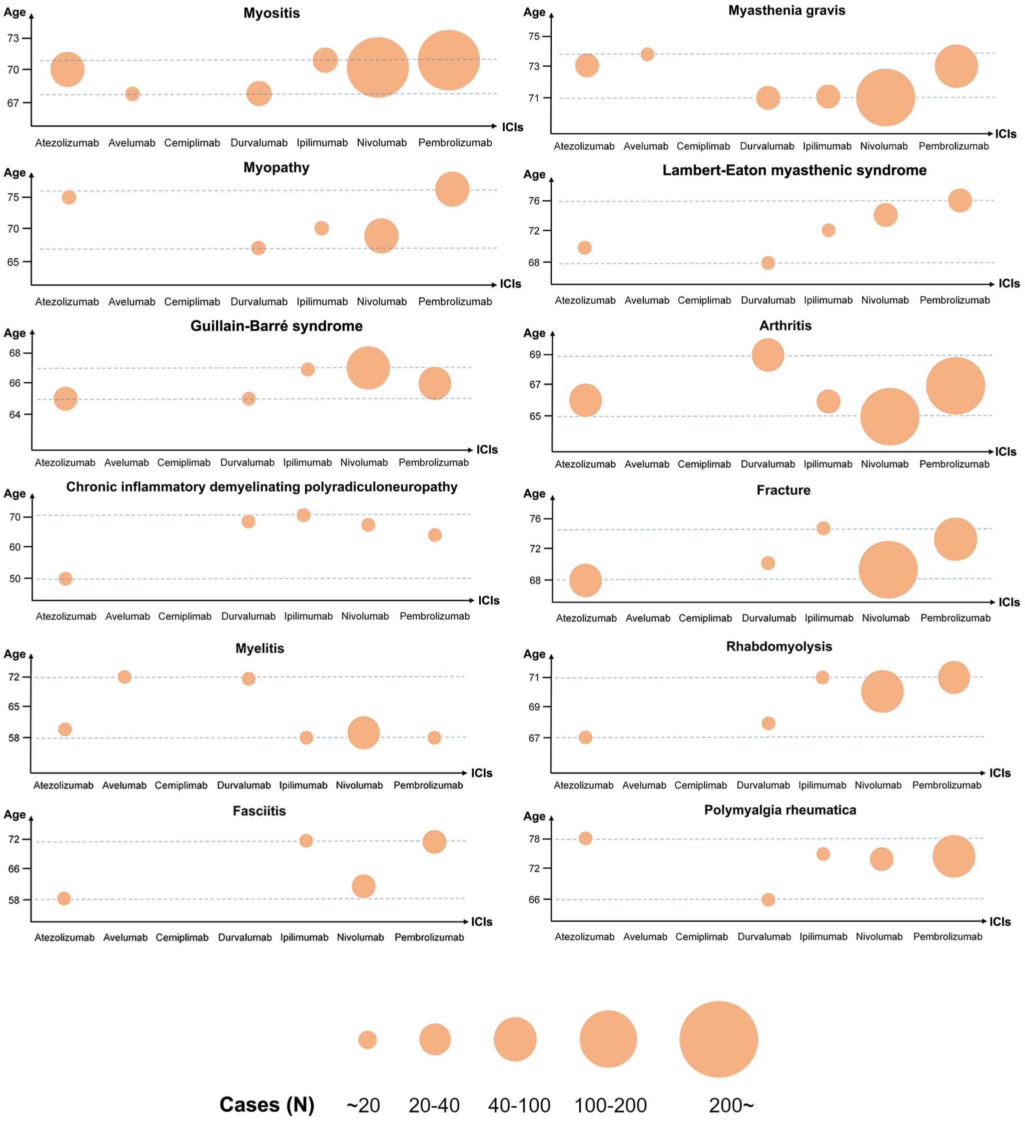

Supplementary Figure S1. Bubble chart of age distribution of the major musculoskeletal adverse events. Bubble size represents sample size.

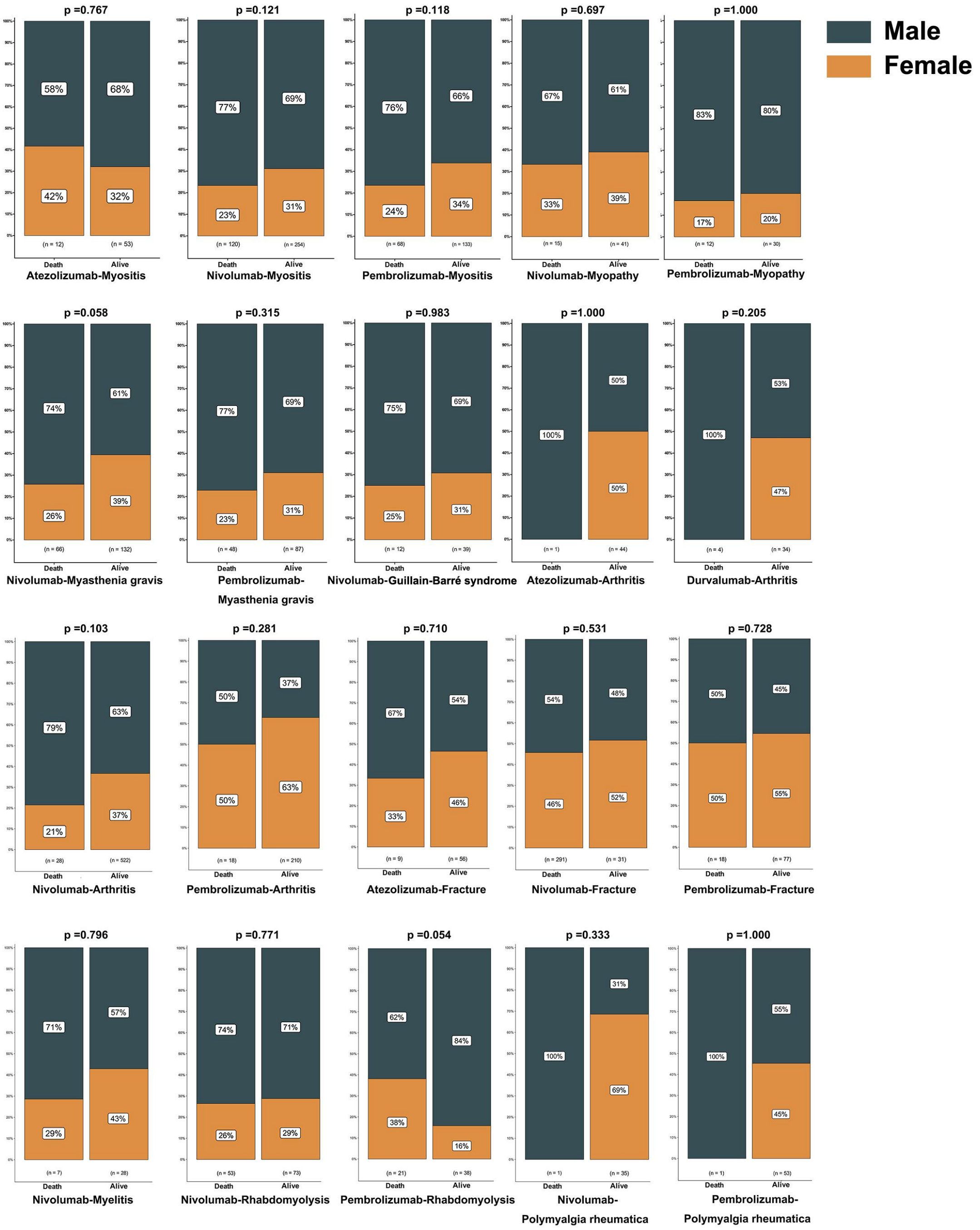

Supplementary Figure S2. Sex differences between the mortality and survival groups in the major musculoskeletal adverse events. The p-value was obtained from a chi-square test.

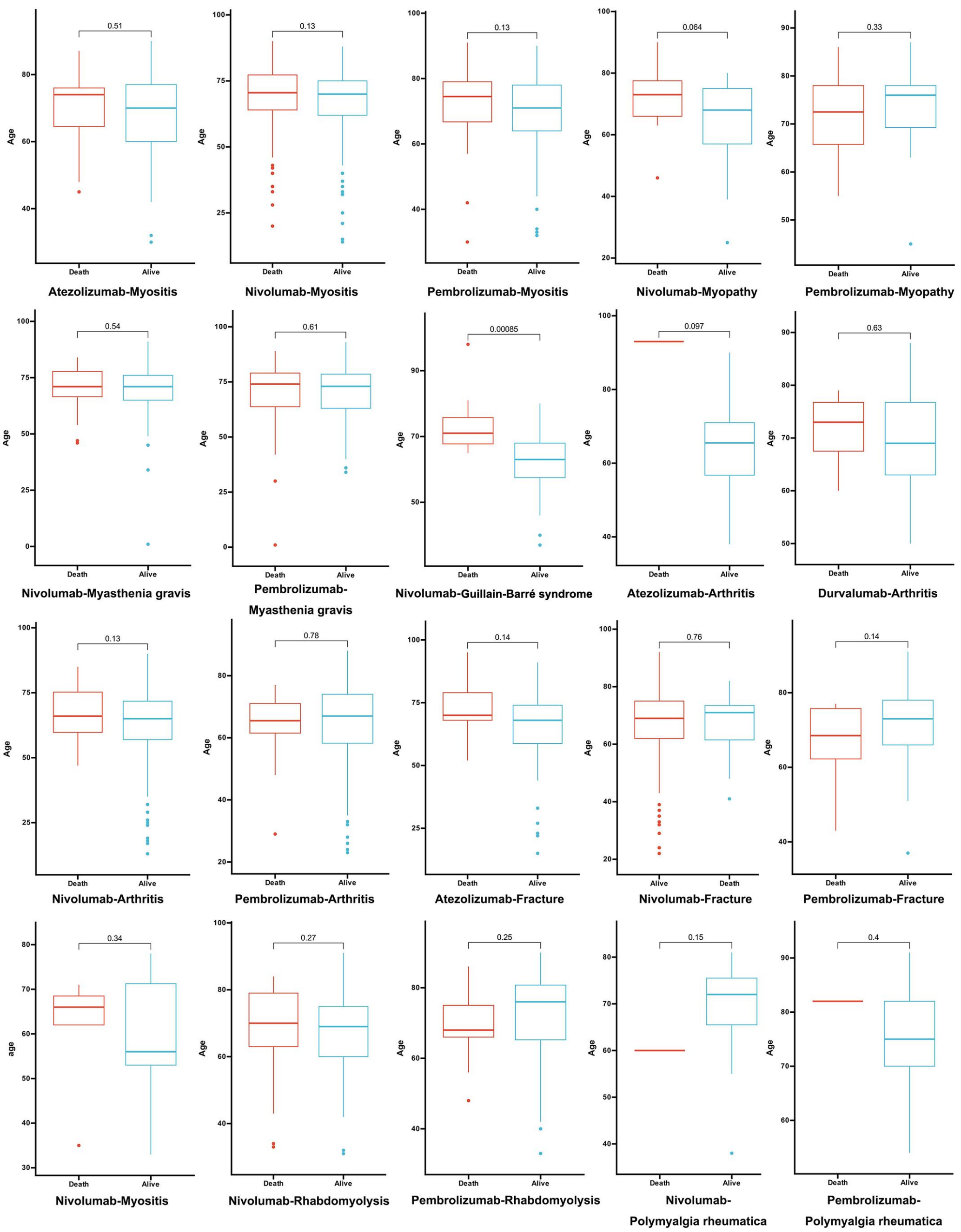

Supplementary Figure S3. Age differences between the mortality and survival groups in the major musculoskeletal adverse events. The p-value was obtained from a independent sample t-test. In cases where the data did not follow a normal distribution, the Mann-Whitney U test was employed. The Welch's t-test was utilized when the data did not meet the homogeneity of variance assumption.

Myositis

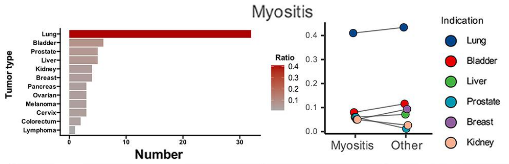

Tumor type	χ^2	p value	OR
Prostate	11.978	0.001	5.33
Kidney	0.748	0.387	1.85
Liver	0.208	0.648	0.81
Lung	1.147	0.284	0.79
Bladder	1.703	0.192	0.58
Breast	2.180	0.140	0.48

Myasthenia gravis

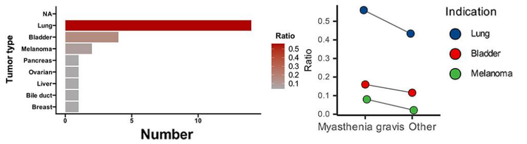

Tumor type	χ^2	p value	OR
Melanoma	-	0.100	3.99
Lung	1.628	0.202	1.66
Bladder	0.146	0.702	1.46

Lambert-Eaton myasthenic syndrome

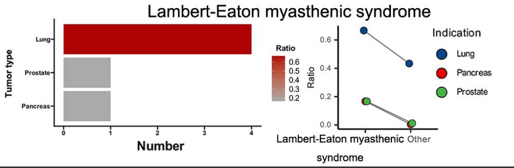

Tumor type	χ^2	p value	OR
Pancreas	-	0.024	50.38
Prostate	-	0.071	16.48
Lung	0.547	0.459	2.61

Guillain-Barré syndrome

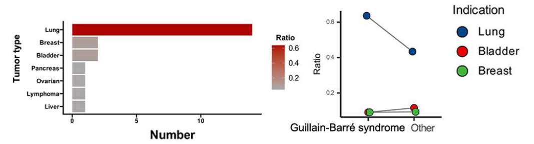

Tumor type	χ^2	p value	OR
Lung	2.194	0.139	1.83
Breast	0	1.000	0.88
Bladder	0.031	0.861	0.69

Arthritis

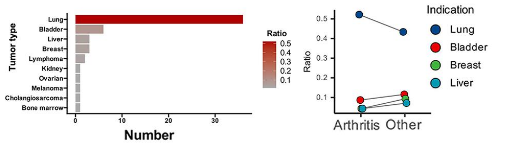

Tumor type	χ^2	p value	OR
Lung	2.196	0.138	1.43
Bladder	0.559	0.455	0.73
Breast	2.05	0.152	0.44
Liver	0.453	0.501	0.59

Fracture

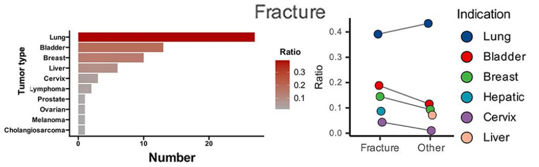

Tumor type	χ^2	p value	OR
Lung	0.510	0.475	0.84
Bladder	3.609	0.057	1.79
Liver	0.071	0.790	1.24
Cervix	-	0.033	4.55

Myelitis

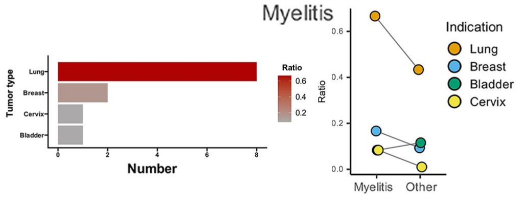

Tumor type	χ^2	p value	OR
Lung	2.655	0.103	2.62
Breast	0.141	0.707	1.94
Bladder	0.000	1.000	0.70
Cervix	-	0.116	8.92

Rhabdomyolysis

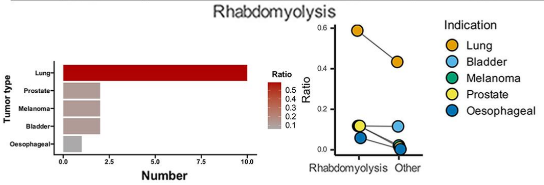

Tumor type	χ^2	p value	OR
Prostate	-	0.018	10.97
Melanoma	-	0.051	6.12
Lung	1.656	0.198	1.87
Bladder	0.000	1.000	1.02

Supplementary Figure S4. Distribution of tumor types in the major musculoskeletal adverse events induced by atezolizumab. This analysis was designed as a case-control analysis. Reports were grouped into two categories based on the presence or absence of the target adverse event (such as myositis), and the odds ratio (OR) were calculated for tumor types.

Myositis

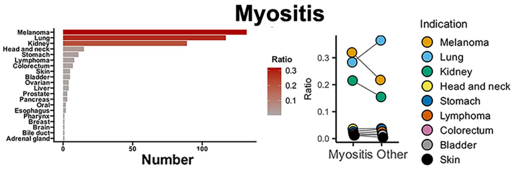

Tumor type	χ2	p value	OR
Skin	3.981	0.046	2.81
Melanoma	25.366	0.000	1.70
Kidney	11.852	0.001	1.51
Head and neck	0.186	0.666	1.12
Colorectum	0.004	0.953	1.02
Head and neck	0.024	0.877	0.96
Bladder	0.223	0.637	0.81
Stomach	0.950	0.330	0.74
Lymphoma	0.820	0.365	0.72
Lung	11.916	0.001	0.69

Myopathy

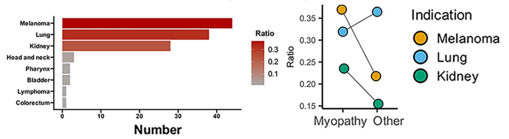

Tumor type	χ2	p value	OR
Melanoma	16.160	0.000	2.11
Kidney	5.950	0.015	1.69
Lung	1.056	0.304	0.82

Myasthenia gravis

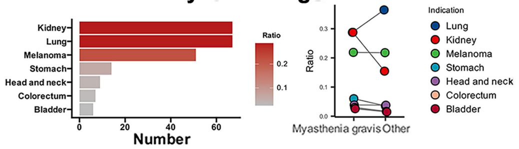

Tumor type	χ2	p value	OR
Kidney	31.715	0.000	2.22
Colorectum	1.842	0.175	1.85
Stomach	4.158	0.041	1.75
Bladder	1.209	0.272	1.75
Head and neck	0.005	0.944	1.02
Melanoma	0.001	0.970	1.01
Lung	6.008	0.014	0.70

Lambert-Eaton myasthenic syndrome

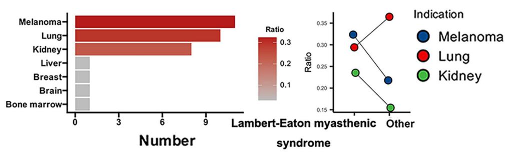

Tumor type	χ2	p value	OR
Melanoma	2.230	0.135	1.72
Kidney	1.696	0.193	1.68
Lung	0.730	0.393	0.73

Guillain-Barré syndrome

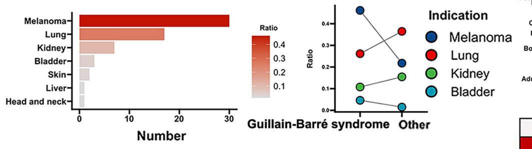

Tumor type	χ2	p value	OR 5
Bladder	-	0.073	3.21
Melanoma	22.688	0.000	3.08
Kidney	1.096	0.295	0.66
Lung	2.986	0.084	0.62

Arthritis

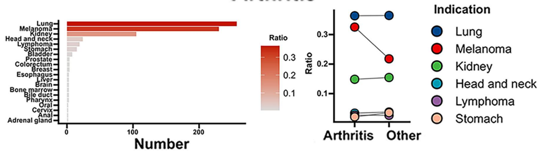

Tumor type	χ2	p value	OR
Melanoma	48.809	0.000	1.75
Lymphoma	0.004	0.949	1.02
Lung	0.004	0.951	1.00
Kidney	0.203	0.652	0.95
Head and neck	0.288	0.592	0.90
Stomach	4.271	0.039	0.59

Fracture

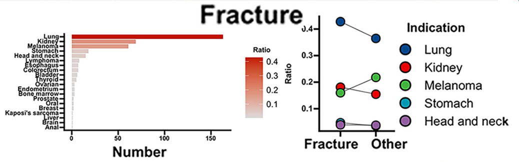

Tumor type	χ2	p value	OR
Stomach	1.564	0.211	1.35
Lung	6.638	0.010	1.31
Kidney	2.070	0.150	1.21
Head and neck	0.028	0.868	1.04
Melanoma	7.534	0.006	0.68

Myelitis

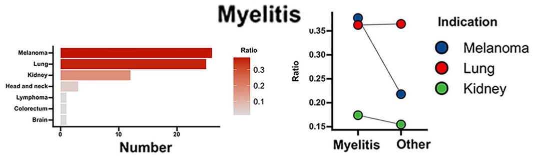

Tumor type	χ2	p value	OR
Melanoma	10.249	0.001	2.17
Head and neck	0.000	1.000	1.16
Kidney	0.198	0.567	1.15
Lung	0.002	0.968	0.99

Rhabdomyolysis

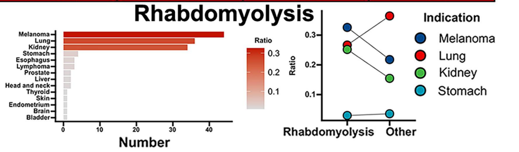

Tumor type	χ2	p value	OR
Melanoma	9.284	0.002	1.74
Kidney	9.807	0.002	1.15
Stomach	0.018	0.894	0.83
Lung	5.609	0.018	0.63

Polymyalgia rheumatica

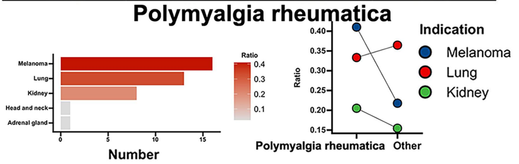

Tumor type	χ2	p value	OR
Melanoma	8.480	0.004	2.5
Kidney	0.763	0.382	1.41
Lung	0.165	0.685	0.87

Supplementary Figure S5. Distribution of tumor types in the major musculoskeletal adverse events induced by nivolumab. This analysis was designed as a case-control analysis. Reports were grouped into two categories based on the presence or absence of the target adverse event (such as myositis), and the odds ratio (OR) were calculated for tumor types.

Myositis

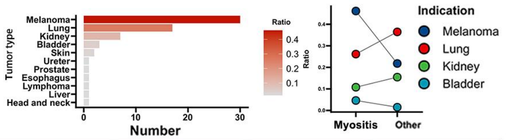

Tumor type	χ2	p value	OR
Lymphoma	17.742	0.000	3.56
Melanoma	4.242	0.039	1.36
Bladder	0.371	0.543	1.16
Lung	0.575	0.448	0.90

Myasthenia gravis

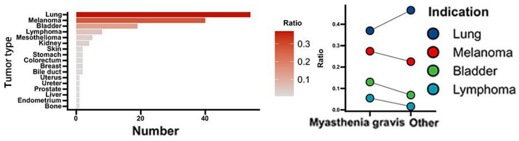

Tumor type	χ2	p value	OR
Lymphoma	11.825	0.001	3.66
Bladder	8.522	0.004	2.03
Melanoma	2.006	0.157	1.30
Lung	5.409	0.020	0.67

Guillain-Barré syndrome

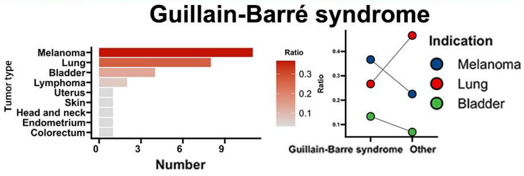

Tumor type	χ2	p value	OR
Melanoma	3.446	0.063	1.99
Lung	4.774	0.029	0.42
Bladder	1.056	0.304	2.008

Fracture

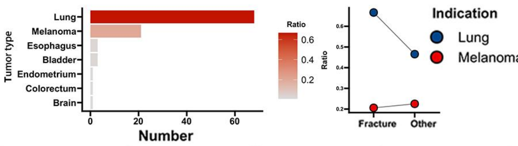

Tumor type	χ2	p value	OR
Lung	16.693	0.000	2.31
Melanoma	0.220	0.639	0.89

Fasciitis

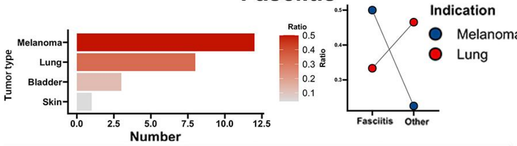

Tumor type	χ2	p value	OR
Melanoma	10.400	0.001	3.45
Lung	1.687	0.194	0.57

Polymyalgia rheumatica

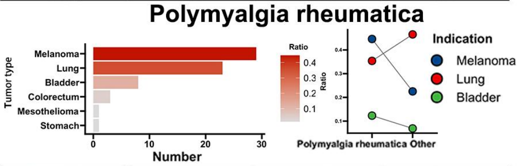

Tumor type	χ2	p value	OR
Colorectum	2.119	0.145	3.01
Melanoma	18.252	0.000	2.78
Bladder	2.171	0.141	1.90
Lung	3.267	0.710	0.63

Myopath

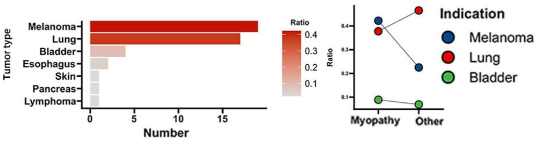

Tumor type	χ2	p value	OR
Melanoma	10.035	0.020	2.52
Bladder	0.053	0.819	1.32
Lung	1.394	0.238	0.70

Lambert-Eaton myasthenic syndrome

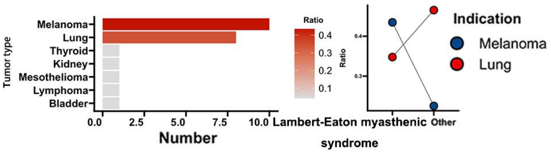

Tumor type	χ2	p value	OR
Melanoma	5.797	0.016	2.65
Lung	1.281	0.258	0.61

Arthritis

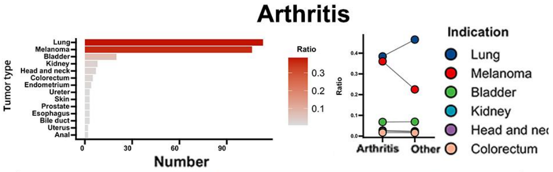

Tumor type	χ2	p value	OR
Melanoma	31.390	0.000	1.97
Head and neck	0.302	0.583	1.24
Kidney	0.196	0.658	1.17
Colorectum	0.000	1.000	1.07
Bladder	0.005	0.941	0.98
Lung	7.909	0.005	0.71

Myelitis

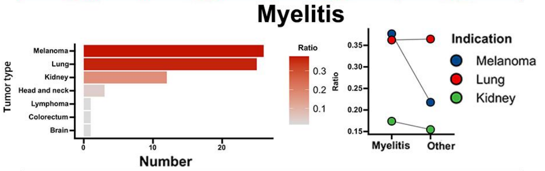

Tumor type	χ2	p value	OR
Melanoma	10.249	0.001	2.17
Head and neck	0.000	1.000	1.16
Kidney	0.198	0.567	1.15
Lung	0.002	0.968	0.99

Rhabdomyolysis

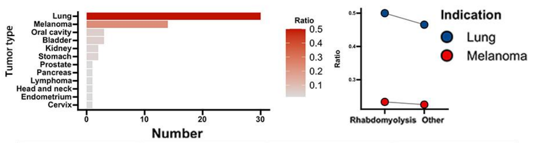

Tumor type	χ2	p value	OR
Melanoma	0.023	0.880	1.05
Lung	0.289	0.591	1.15

Supplementary Figure S6. Distribution of tumor types in the major musculoskeletal adverse events induced by pembrolizumab. This analysis was designed as a case-control analysis. Reports were grouped into two categories based on the presence or absence of the target adverse event (such as myositis), and the odds ratio (OR) were calculated for tumor types.

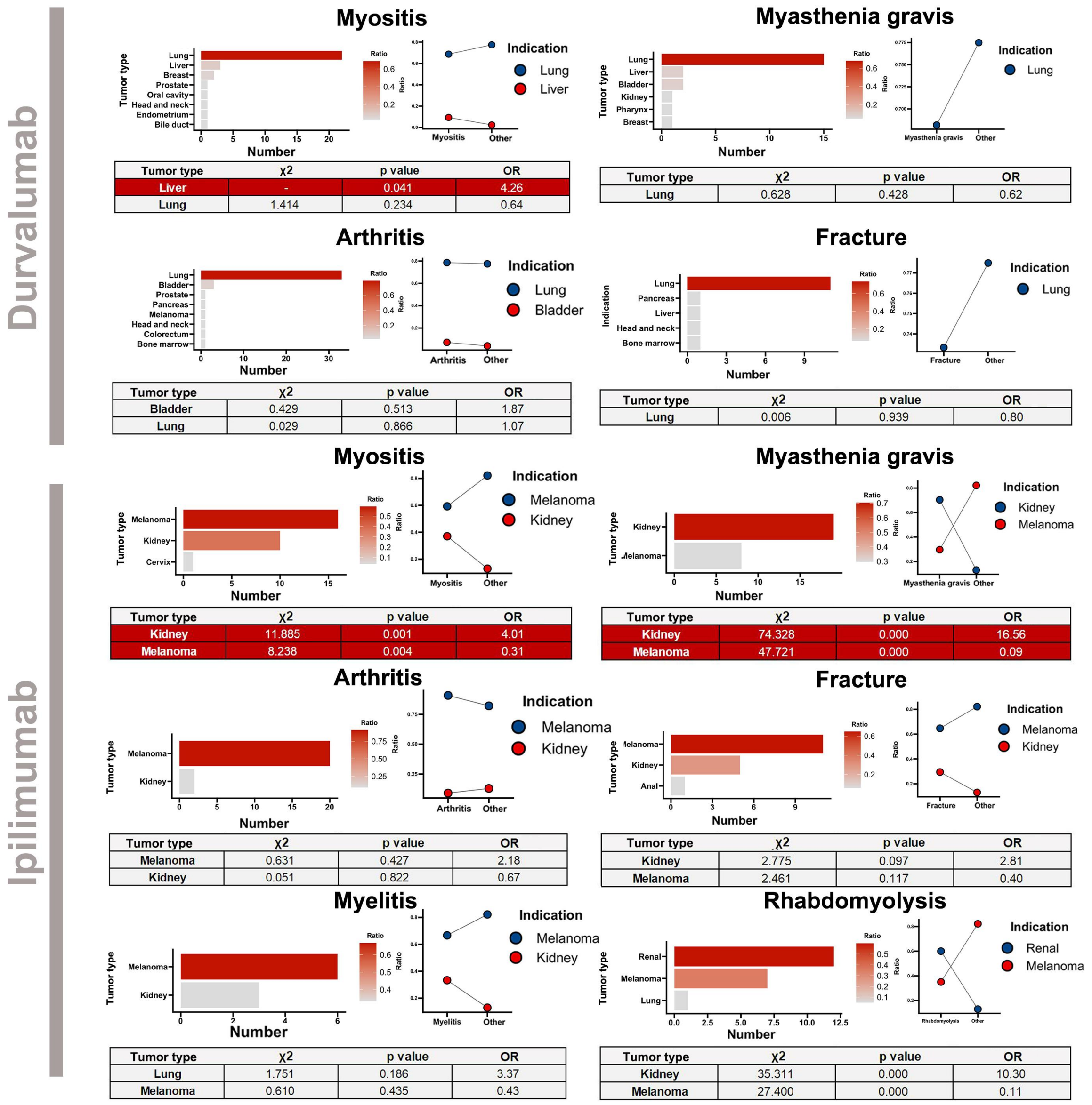

Durvalumab — Myositis

Tumor type	χ2	p value	OR
Liver	-	0.041	4.26
Lung	1.414	0.234	0.64

Durvalumab — Myasthenia gravis

Tumor type	χ2	p value	OR
Lung	0.628	0.428	0.62

Durvalumab — Arthritis

Tumor type	χ2	p value	OR
Bladder	0.429	0.513	1.87
Lung	0.029	0.866	1.07

Durvalumab — Fracture

Tumor type	χ2	p value	OR
Lung	0.006	0.939	0.80

Ipilimumab — Myositis

Tumor type	χ2	p value	OR
Kidney	11.885	0.001	4.01
Melanoma	8.238	0.004	0.31

Ipilimumab — Myasthenia gravis

Tumor type	χ2	p value	OR
Kidney	74.328	0.000	16.56
Melanoma	47.721	0.000	0.09

Ipilimumab — Arthritis

Tumor type	χ2	p value	OR
Melanoma	0.631	0.427	2.18
Kidney	0.051	0.822	0.67

Ipilimumab — Fracture

Tumor type	χ2	p value	OR
Kidney	2.775	0.097	2.81
Melanoma	2.461	0.117	0.40

Ipilimumab — Myelitis

Tumor type	χ2	p value	OR
Lung	1.751	0.186	3.37
Melanoma	0.610	0.435	0.43

Ipilimumab — Rhabdomyolysis

Tumor type	χ2	p value	OR
Kidney	35.311	0.000	10.30
Melanoma	27.400	0.000	0.11

Supplementary Figure S7. Distribution of tumor types in the major musculoskeletal adverse events induced by durvalumab and ipilimumab. This analysis was designed as a case-control analysis. Reports were grouped into two categories based on the presence or absence of the target adverse event (such as myositis), and the odds ratio (OR) were calculated for tumor types.
