# Supplementary figures and images for: Musculoskeletal adverse events induced by immune checkpoint inhibitors: a large-scale pharmacovigilance study
Source: Front Pharmacol. 2023 Oct 10;14:1199031. doi: 10.3389/fphar.2023.1199031 (PMC10595016; doi:10.3389/fphar.2023.1199031)

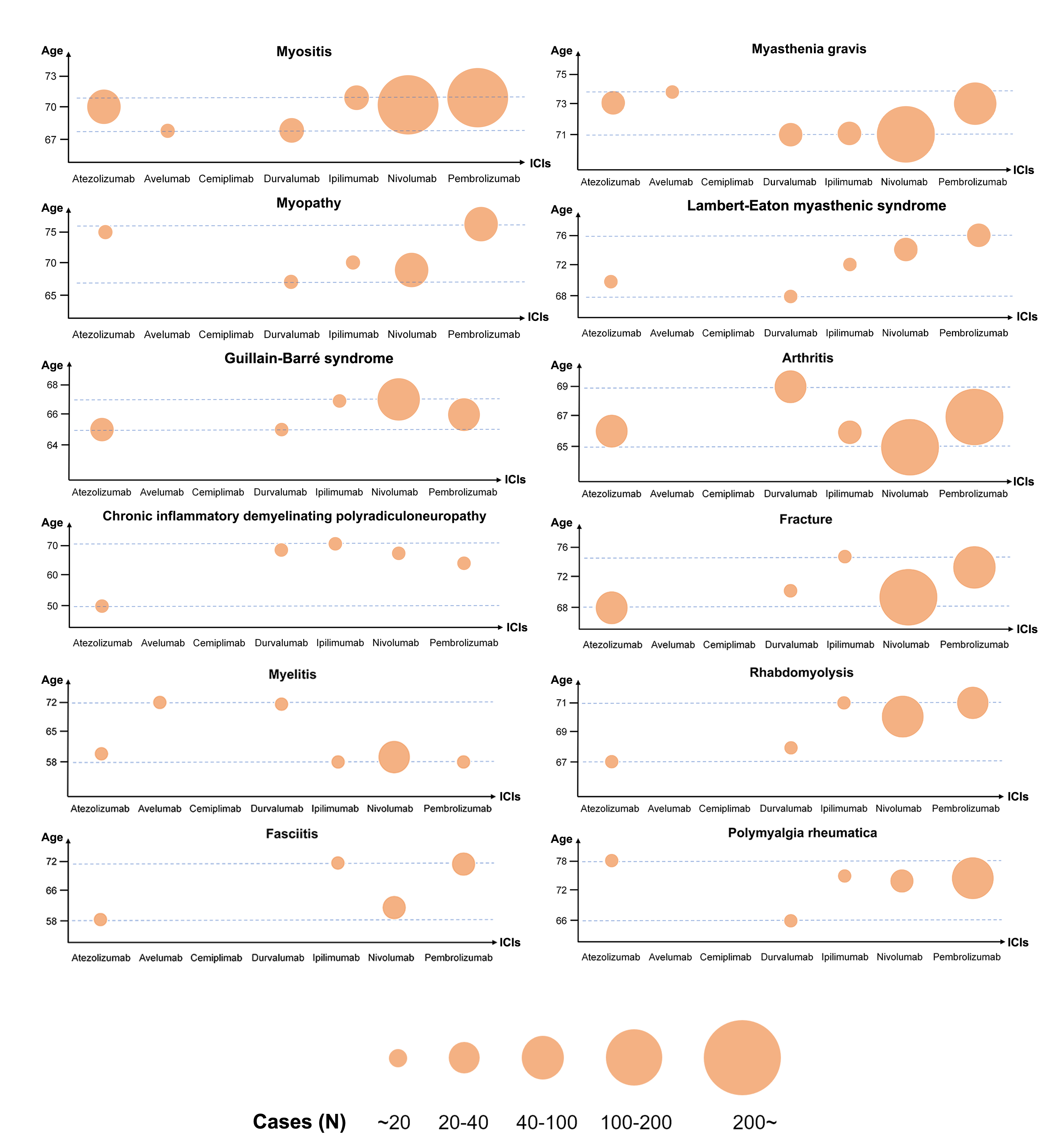

Supplement: Supplementary file 2 [file DataSheet1.zip › Supplementary Figures/Figure S1.tif]

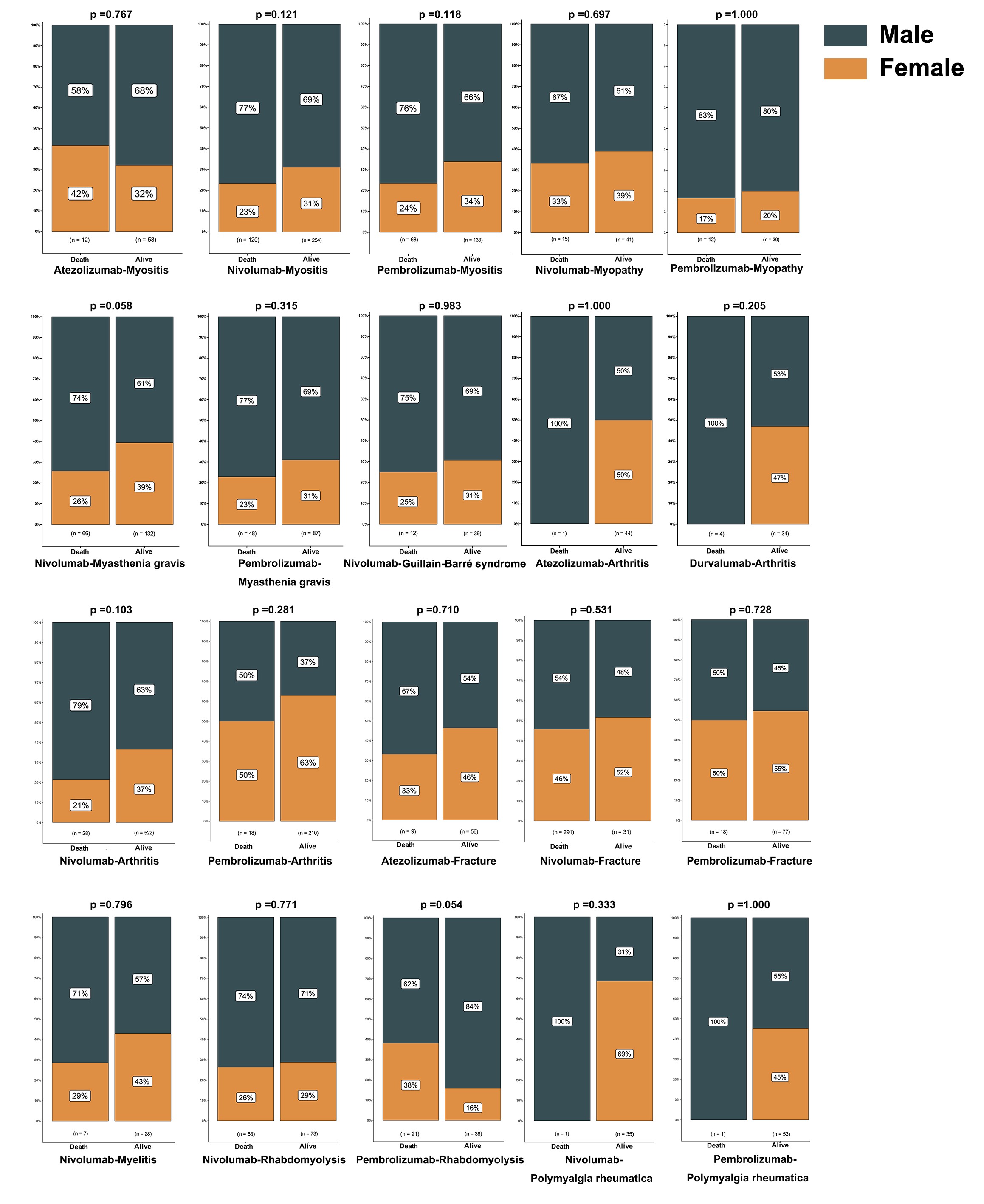

Supplement: Supplementary file 2 [file DataSheet1.zip › Supplementary Figures/Figure S2.tif]

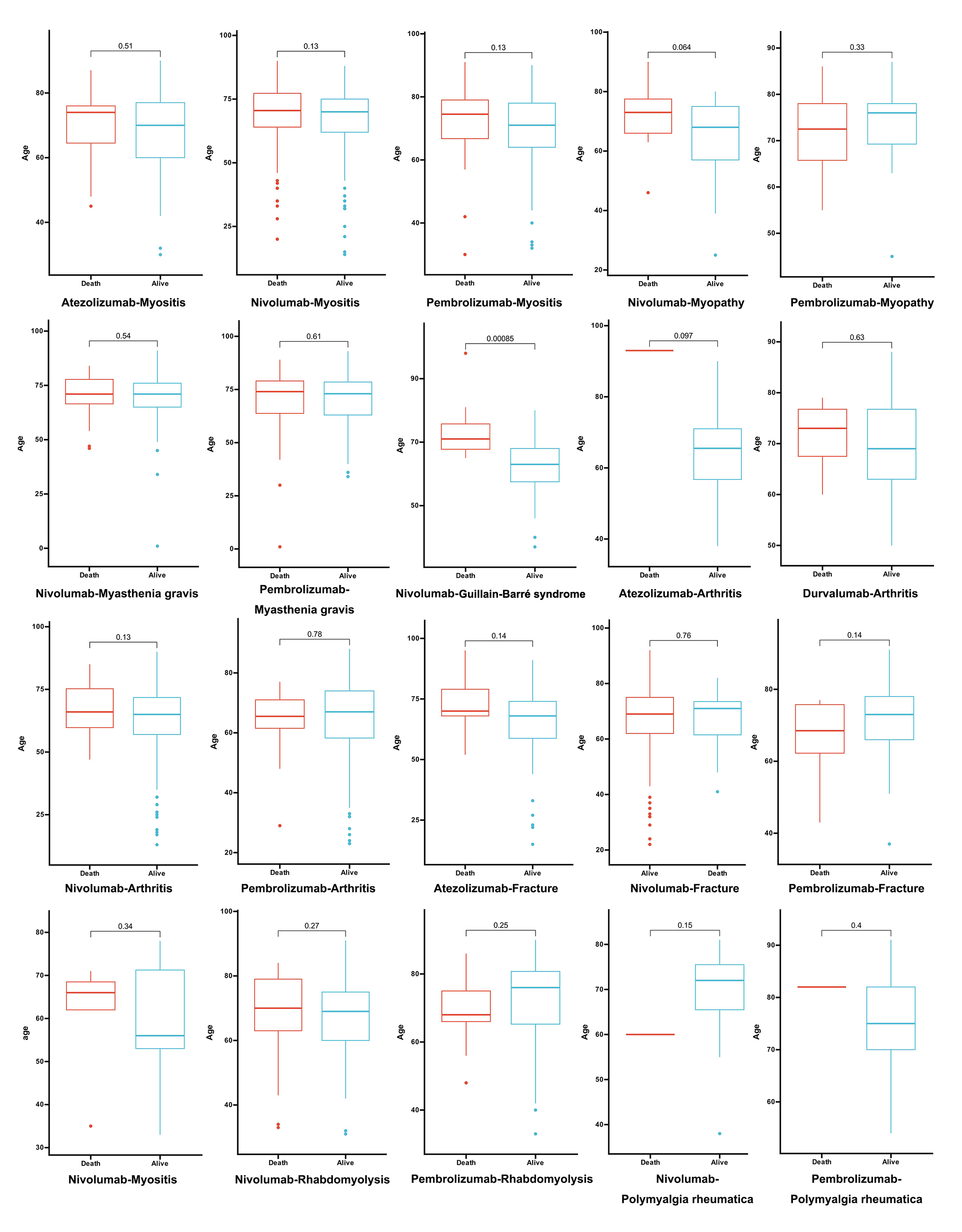

Supplement: Supplementary file 2 [file DataSheet1.zip › Supplementary Figures/Figure S3.tif]

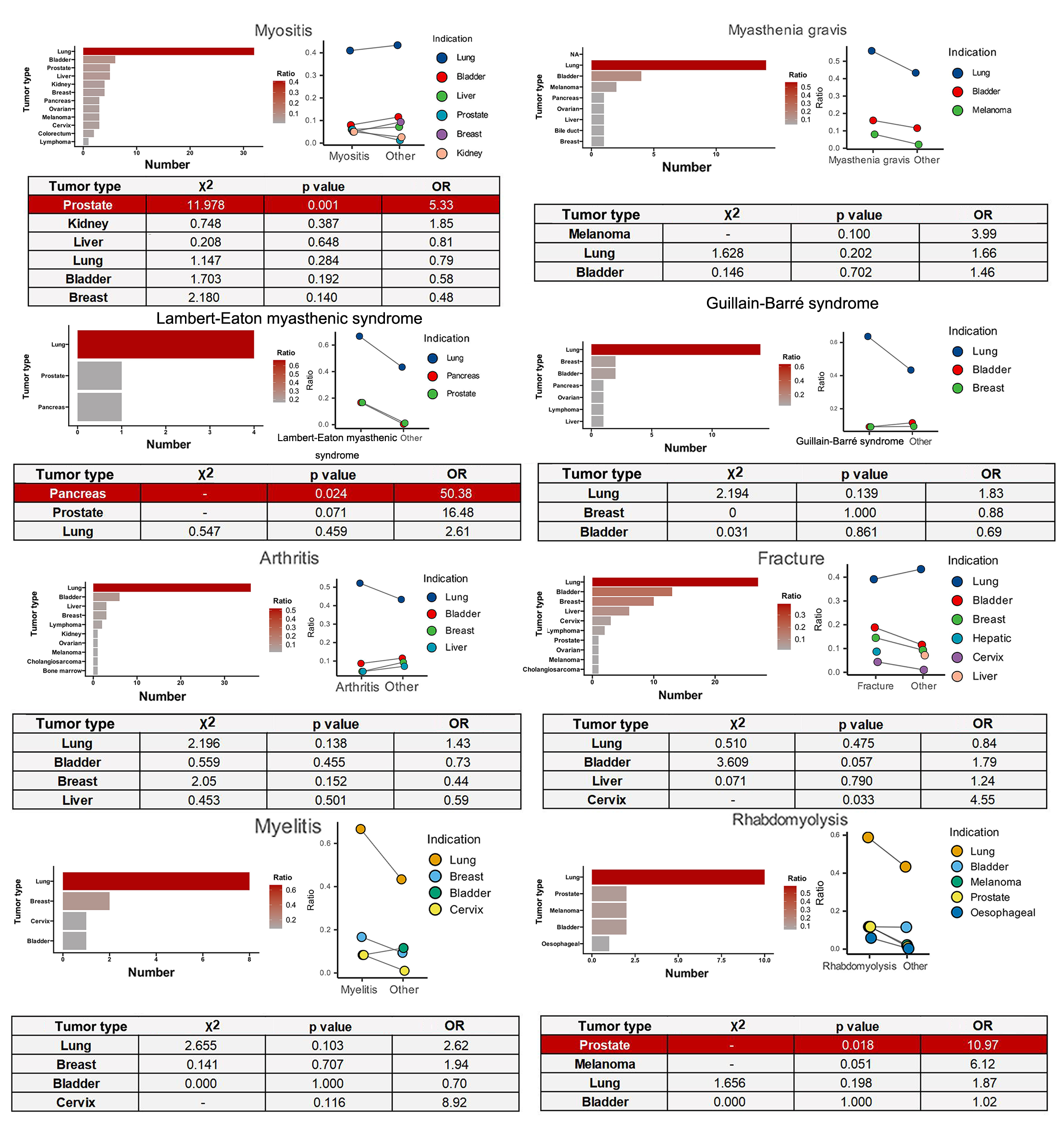

Supplement: Supplementary file 2 [file DataSheet1.zip › Supplementary Figures/Figure S4.tif]

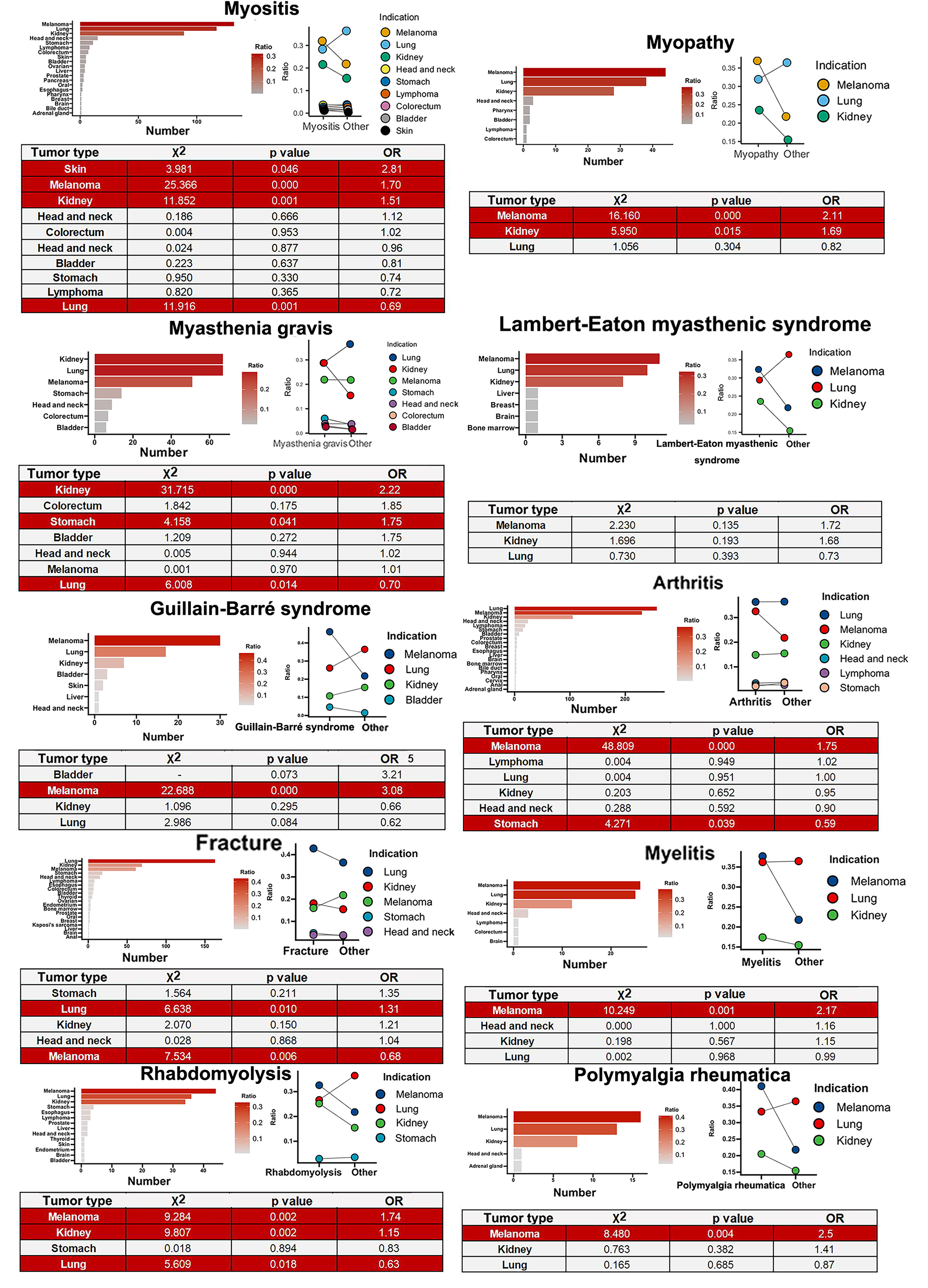

Supplement: Supplementary file 2 [file DataSheet1.zip › Supplementary Figures/Figure S5.tif]

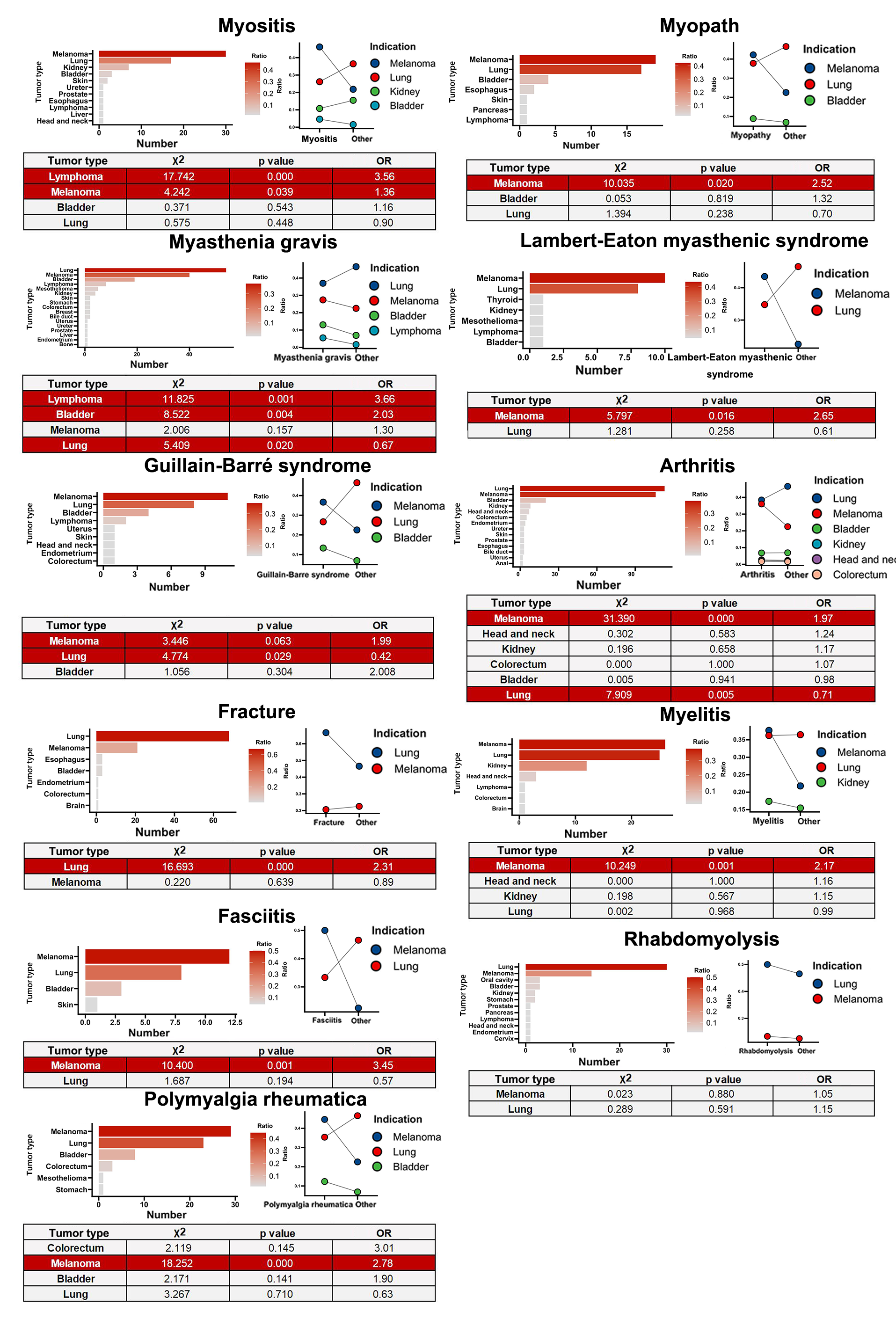

Supplement: Supplementary file 2 [file DataSheet1.zip › Supplementary Figures/Figure S6.tif]

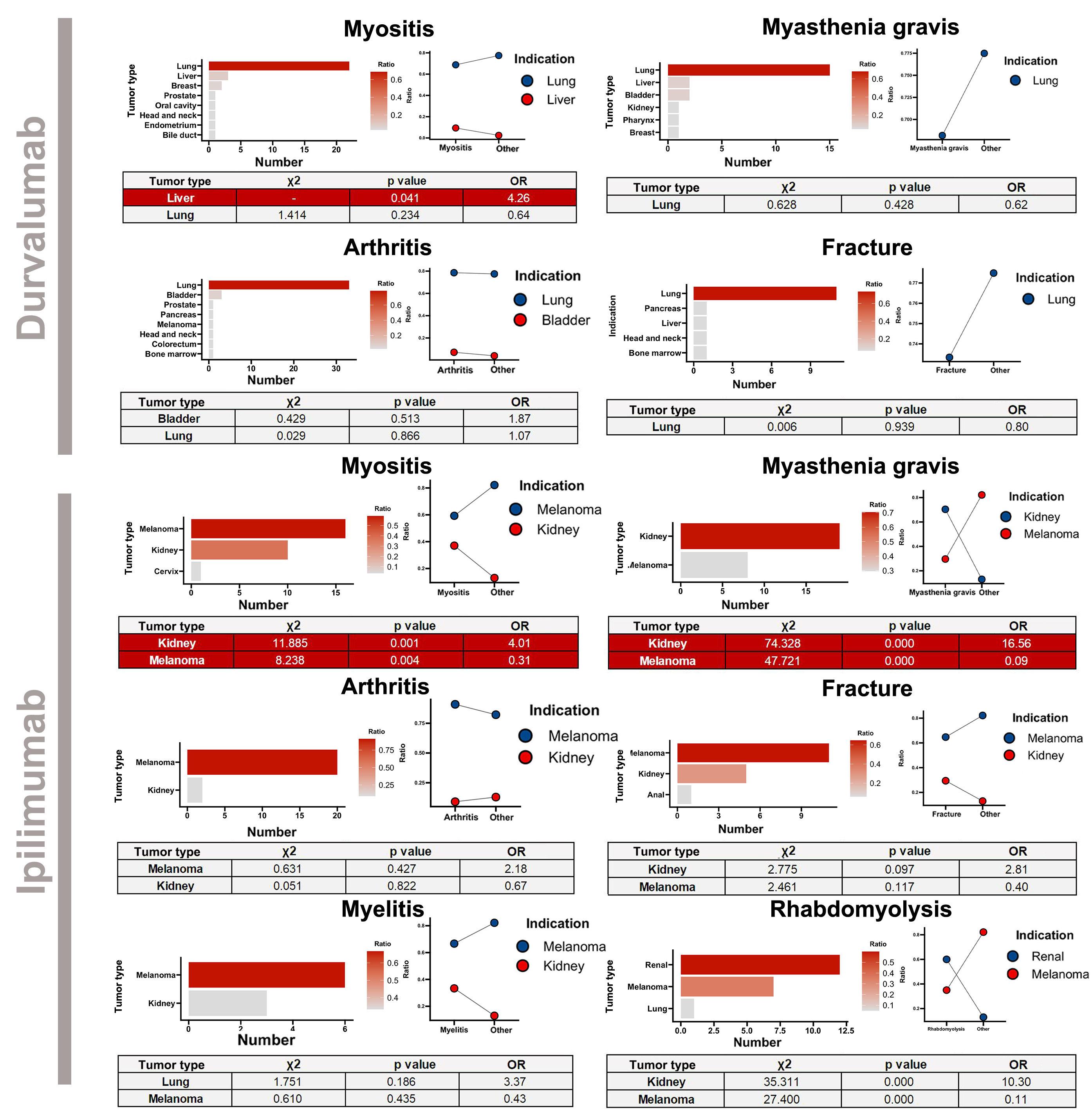

Supplement: Supplementary file 2 [file DataSheet1.zip › Supplementary Figures/Figure S7.tif]
