# Supplementary material for: Musculoskeletal adverse events induced by immune checkpoint inhibitors: a large-scale pharmacovigilance study
Source: Front Pharmacol. 2023 Oct 10;14:1199031. doi: 10.3389/fphar.2023.1199031 (PMC10595016; doi:10.3389/fphar.2023.1199031)
Supplement: Supplementary file 3 [file Table2.DOCX]

Supplementary Table S2. Baseline information of ICIs-induced musculoskeletal AEs.

| **AEs** | **Gender^a^** | | **Age** | | **Outcome^b^** | | **Tumor type^c^** | | |
| --- | --- | --- | --- | --- | --- | --- | --- | --- | --- |
|  | **Male** | **Female** | **Median** | **Range** | **Death** | **Alive** | **Top1** | **Top2** | **Top3** |
| acetabulum fracture | 4 | 0 | 62 | 58-67 | 1 | 3 | Melanoma (2, 50.0%) | Lung (1, 25.0%) | Oral (1, 25.0%) |
| acute disseminated encephalomyelitis | 1 | 1 | - | 50-58 | 0 | 4 | - | - | - |
| ankle fracture | 7 | 5 | 75 | 59-84 | 0 | 13 | Lung (4, 30.7%) | Melanoma (4, 30.7%) | Kidney (1, 7.6%) |
| ankylosing spondylitis | 3 | 2 | 58 | 45-76 | 1 | 7 | Lung (3, 37.5%) | Melanoma (1, 12.5%) | Kidney (1, 12.5%) |
| arthralgia | 544 | 365 | 65 | 7-91 | 85 | 1237 | Lung (483, 36.5%) | Melanoma (371, 28.0%) | Kidney (128, 9.6%) |
| arthritis reactive | 2 | 1 | 73 | 63-77 |  | 5 | Lung (3, 60.0%) | Bladder (1, 20%) | - |
| autoimmune arthritis | 28 | 22 | 63 | 35-86 | 2 | 55 | Melanoma (26, 45.6%) | Lung (9, 15.7%) | Kidney (8, 14.0%) |
| autoimmune myositis | 8 | 6 | 70 | 55-86 | 8 | 8 | Lung (5, 31.2%) | Melanoma (4, 25.0%) | Bladder (1, 6.2%) |
| bone density abnormal | 1 | 0 | - | 89 | 0 | 1 | - | - | - |
| bone density decreased | 1 | 0 | - | 39 | 0 | 4 | Melanoma (2, 50.0%) | Lung (1, 25.0%) | Lung (1, 25.0%) |
| bone lesion | 14 | 5 | 68 | 16-89 | 3 | 20 | Lung (7, 30.4%) | Kidney (5, 21.7%) | Melanoma (3, 13.0%) |
| cervical spinal stenosis | 3 | 0 | 65 | 64-67 | 0 | 3 | - | - | - |
| cervical vertebral fracture | 5 | 3 | 74 | 48-90 | 2 | 8 | Lung (4, 40.0%) | Melanoma (2, 20.0%) | Kidney (2, 20.0%) |
| chondritis | 4 | 0 | 71 | 61-79 | 0 | 5 | Head and neck (3, 60.0%) | Lung (1, 20.0%) | Melanoma (1, 20.0%) |
| chondrocalcinosis | 1 | 0 | - | 76 | 0 | 2 | - | - | - |
| chondrocalcinosis pyrophosphate | 6 | 2 | 72 | 63-83 | 1 | 13 | Lung (5, 35.7%) | Kidney (3, 21.4%) | Melanoma (2, 14.2%) |
| chronic inflammatory demyelinating polyradiculoneuropathy | 14 | 7 | 67 | 27-85 | 1 | 24 | Melanoma (10, 40.0%) | Lung (3, 12.0%) | Lymphoma (3, 12.0%) |
| clavicle fracture | 7 | 5 | 69 | 46-79 | 1 | 14 | Lung (6, 40.0%) | Melanoma (6, 40.0%) | Kidney (1, 6.6%) |
| compression fracture | 12 | 7 | 76 | 35-86 | 20 | 55 | Lung (40, 53.3%) | Kidney (7, 9.3%) | Melanoma (5, 6.6%) |
| dermatomyositis | 44 | 14 | 69 | 15-85 | 10 | 59 | Lung (33, 47.8%) | Melanoma (16, 23.1%) | Kidney (4, 5.7%) |
| encephalomyelitis | 4 | 10 | 67 | 39-73 | 3 | 19 | Kidney (6, 27.2%) | Melanoma (4, 18.1%) | Lung (3, 13.6%) |
| eosinophilic fasciitis | 21 | 16 | 59 | 35-89 | 3 | 38 | Melanoma (18, 43.9%) | Lung (12, 29.2%) | Bladder (3, 7.3%) |
| facial bones fracture | 6 | 3 | 72 | 60-86 | 3 | 7 | Lung (3, 30.0%) | Melanoma (2, 20.0%) | Bladder (1, 10.0%) |
| fall | 318 | 203 | 71 | 24-92 | 152 | 482 | Lung (225, 35.4%) | Melanoma (116, 18.2%) | Kidney (70, 11.0%) |
| fasciitis | 39 | 21 | 63 | 35-89 | 9 | 62 | Melanoma (30, 42.2%) | Lung (17, 23.9%) | Bladder (7, 9.8%) |
| femoral neck fracture | 14 | 13 | 75 | 56-85 | 10 | 23 | Lung (17, 51.5%) | Melanoma (4, 12.1%) | Stoma (3, 9.0%) |
| femur fracture | 27 | 24 | 70 | 29-92 | 10 | 58 | Lung (31, 45.5%) | Melanoma (9, 13.2%) | Kidney (8, 11.7%) |
| fibula fracture | 1 | 1 | - | 39-70 | 0 | 2 | - | - | - |
| foot fracture | 5 | 3 | 66 | 33-85 | 1 | 9 | Lung (3, 30.0%) | Melanoma (2, 20.0%) | Ovarian (2, 20.0%) |
| forearm fracture | 1 | 1 | - | 68-71 | 0 | 2 | - | - | - |
| fractured coccyx | 1 | 0 | - | 80 | 0 | 1 | - | - | - |
| fractured ischium | 0 | 2 | - | 75-83 | 1 | 1 | - | - | - |
| fractured sacrum | 1 | 3 | 63 | 37-64 | 1 | 3 | Lung (3, 75.0%) | Melanoma (1, 25.0%) | - |
| gouty arthritis | 6 | 0 | 78 | 65-82 | 0 | 6 | Melanoma (4, 66.6%) | Lung (2, 33.3%) | - |
| Guillain-Barre syndrome | 74 | 39 | 66 | 25-98 | 32 | 123 | Melanoma (51, 32.9%) | Lung (42, 27.0%) | Bladder (9, 5.8%) |
| hand fracture | 3 | 1 | 72 | 49-80 | 0 | 5 | Melanoma (2, 40.0%) | Lung (1, 20.0%) | Liver (1, 20.0%) |
| hip fracture | 20 | 27 | 70 | 44-91 | 18 | 49 | Lung (21, 31.3%) | Melanoma (12, 17.9%) | Kidney (7, 10.4%) |
| humerus fracture | 6 | 7 | 71 | 52-86 | 2 | 15 | Lung (5, 29.4%) | Melanoma (5, 11.7%) | Kidney (2, 11.7%) |
| hypertrophic osteoarthropathy | 2 | 0 | - | 42-54 | 0 | 2 | - | - | - |
| ilium fracture | 2 | 0 | - | 62-78 | 1 | 2 | - | - | - |
| immune-mediated arthritis | 27 | 16 | 70 | 32-84 | 1 | 48 | Lung (15, 30.6%) | Melanoma (11, 22.4%) | Bladder (8, 16.3%) |
| immune-mediated myositis | 36 | 16 | 71 | 33-89 | 18 | 36 | Lung (16, 29.6%) | Melanoma (16, 29.6%) | Kidney (7, 12.9%) |
| immune-mediated necrotising myopathy/necrotising myositis | 22 | 8 | 76 | 53-86 | 11 | 21 | Lung (13, 40.6%) | Melanoma (9, 28.1%) | Kidney (3, 9.3%) |
| inclusion body myositis | 3 | 0 | 75 | 75-85 | 1 | 2 | - | - | - |
| juvenile idiopathic arthritis | 0 | 1 | - | 68 | 0 | 2 | - | - | - |
| limb fracture | 9 | 16 | 66 | 24-90 | 5 | 26 | Lung (12, 38.7%) | Melanoma (6, 19.3%) | Kidney (3, 9.6%) |
| lower limb fracture | 6 | 6 | 68 | 24-84 | 1 | 14 | Lung (7, 46.6%) | Melanoma (2, 46.6%) | Kidney (2, 13.3%) |
| lumbar spinal stenosis | 4 | 2 | 70 | 28-82 | 0 | 5 | Lung (1, 20.0%) | Melanoma (1, 20.0%) | Kidney (1, 20.0%) |
| lumbar vertebral fracture | 15 | 9 | 71 | 50-89 | 7 | 20 | Lung (11, 40.7%) | Melanoma (4, 14.8%) | Kidney (4, 14.8%) |
| mitochondrial myopathy | 0 | 1 | - | 44 | 0 | 3 | Melanoma (3, 100%) | - | - |
| monarthritis | 1 | 0 | - | 65 | 0 | 1 | - | - | - |
| multiple fractures | 3 | 2 | 67 | 58-73 | 0 | 5 | Lung (3, 60.0%) | Melanoma (2, 40.0%) | - |
| muscle necrosis | 3 | 3 | 64 | 53-76 | 1 | 7 | Lung (81, 37.5%) | Melanoma (67, 37.5%) | Breast (48, 12.5%) |
| muscle spasms | 70 | 66 | 64 | 29-87 | 12 | 206 | Lung (66, 30.2%) | Melanoma (63, 28.8%) | Kidney (22, 10.0%) |
| muscular weakness | 313 | 178 | 70 | 22-95 | 114 | 554 | Lung (216, 32.3%) | Melanoma (170, 25.4%) | Kidney (57, 8.5%) |
| musculoskeletal pain | 135 | 69 | 65 | 14-93 | 35 | 259 | Lung (116, 39.4%) | Melanoma (63, 21.4%) | Kidney (42, 14.2%) |
| musculoskeletal stiffness | 42 | 38 | 67 | 23-85 | 10 | 127 | Lung (44, 32.1%) | Melanoma (42, 30.6%) | Kidney (15, 10.9%) |
| myasthenia gravis | 270 | 137 | 72 | 30-93 | 160 | 336 | Lung (151, 30.4%) | Melanoma (102, 20.5%) | Kidney (93, 18.7%) |
| myasthenia gravis crisis | 12 | 13 | 69 | 34-83 | 5 | 22 | Melanoma (8, 29.6%) | Lung (7, 25.9%) | Lymphoma (5, 18.5%) |
| myasthenic syndrome | 36 | 27 | 72 | 32-90 | 24 | 53 | Lung (26, 33.7%) | Melanoma (24, 31.1%) | Kidney (9, 11.6%) |
| myelitis transverse | 12 | 15 | 56 | 33-82 | 1 | 29 | Lung (13, 43.3%) | Melanoma (12, 40.0%) | Kidney (2, 6.6%) |
| myofascitis | 3 | 2 | 64 | 43-68 | 0 | 5 | Kidney (3, 60.0%) | Melanoma (1, 20.0%) | Bladder (1, 20.0%) |
| myositis ossificans | 0 | 1 | - | 89 | 0 | 1 | - | - | - |
| necrotising fasciitis | 9 | 2 | 71 | 48-83 | 4 | 10 | Melanoma (4, 28.5%) | Kidney (4, 28.5%) | Bladder (3, 21.4%) |
| neuralgic amyotrophy | 1 | 2 | 66 | 50-71 | 0 | 3 | - | - | - |
| neuromyelitis optica spectrum disorder | 4 | 2 | 68 | 30-75 | 0 | 7 | Lung (3,42.8%) | Melanoma (1, 14.2%) | Kidney (1, 14.2%) |
| neuromyopathy | 15 | 1 | 75 | 45-87 | 6 | 13 | Melanoma (7, 36.8%) | Lung (6, 31.5%) | Kidney (3, 15.7%) |
| neuropathic arthropathy | 1 | 1 | - | 59-68 | 0 | 2 | - | - | - |
| noninfectious myelitis | 1 | 0 | - | 68 | 1 | 0 | - | - | - |
| non-specific arthritis | 586 | 365 | 66 | 13-93 | 61 | 1230 | Lung (449, 34.7%) | Melanoma (371, 28.7%) | Kidney (120, 9.2%) |
| non-specific arthropathy | 78 | 24 | 67 | 20-82 | 8 | 133 | Lung (54, 38.2%) | Melanoma (33, 23.4%) | Kidney (22, 15.6%) |
| non-specific fracture | 316 | 261 | 70 | 15-95 | 133 | 566 | Lung (297, 42.4%) | Melanoma (112, 16.0%) | Kidney (79, 11.3%) |
| non-specific myelitis | 44 | 40 | 62 | 25-82 | 18 | 95 | Lung (36, 31.8%) | Melanoma (32, 28.3%) | Kidney (14, 12.3%) |
| non-specific myopathy | 82 | 39 | 72 | 25-90 | 33 | 108 | Melanoma (49, 34.7%) | Lung (47, 33.3%) | Kidney (13, 9.2%) |
| non-specific myositis | 511 | 218 | 70 | 14-91 | 255 | 602 | Lung (278, 32.4%) | Melanoma (220, 25.6%) | Kidney (115, 13.4%) |
| oligoarthritis | 5 | 2 | 68 | 42-73 | 0 | 8 | Lung (1) | Melanoma (1) | Kidney (1) |
| orbital myositis | 3 | 4 | 45 | 14-80 | 1 | 7 | Melanoma (4, 50.0%) | Kidney (2, 25.0%) | Lung (1, 12.5%) |
| osteitis | 3 | 3 | 71 | 47-83 | 1 | 6 | Lung (4, 57.1%) | Melanoma (1, 14.2%) | Kidney (1, 14.2%) |
| osteoarthritis | 30 | 14 | 77 | 28-88 | 7 | 44 | Lung (20, 39.2%) | Melanoma (16, 31.3%) | Bladder (2, 3.9%) |
| osteochondrosis | 2 | 1 | 60 | 53-63 | 0 | 4 | Melanoma (2, 50.0%) | Lung (1, 25.0%) | Bladder (1, 25.0%) |
| osteolysis | 11 | 0 | 63 | 37-77 | 2 | 12 | Lung (6, 42.8%) | Kidney (3, 21.4%) | Melanoma (2, 21.4%) |
| osteomalacia | 1 | 0 | - | 58 | 0 | 1 | - | - | - |
| osteonecrosis | 38 | 17 | 66 | 17-89 | 2 | 69 | Lung (40, 56.3%) | Melanoma (12, 16.9%) | Kidney (8, 11.2%) |
| osteonecrosis of jaw | 19 | 8 | 69 | 22-89 | 2 | 29 | Lung (16, 51.6%) | Kidney (7, 22.5%) | Melanoma (5, 16.1%) |
| osteopenia | 1 | 1 | - | 50-79 | 0 | 3 | - | - | - |
| osteoporosis | 8 | 10 | 67 | 46-82 | 1 | 31 | Lung (14, 43.7%) | Melanoma (6, 18.7%) | Kidney (2, 6.2%) |
| osteoporotic fracture | 6 | 5 | 73 | 58-83 | 0 | 11 | Melanoma (5, 45.4%) | Lung (4, 36.3%) | Kidney (1, 9.0%) |
| osteoradionecrosis | 1 | 0 | - | 65 | 0 | 2 | - | - | - |
| osteosclerosis | 3 | 0 | 64 | 54-67 | 0 | 3 | - | - | - |
| patella fracture | 1 | 2 | 82 | 24-84 | 1 | 2 | - | - | - |
| pathological fracture | 30 | 25 | 66 | 27-87 | 19 | 40 | Lung (21, 35.5%) | Kidney (15, 25.4%) | Bladder (3, 5.0%) |
| pelvic fracture | 8 | 4 | 75 | 52-95 | 3 | 10 | Lung (7, 53.8%) | Melanoma (2, 15.3%) | Bladder (2, 15.3%) |
| periarthritis | 4 | 0 | 67 | 60-74 | 0 | 7 | Prostate (2) | Lung (1) | Melanoma (1) |
| plantar fasciitis | 0 | 1 | - | 71 | 0 | 1 | - | - | - |
| polyarthritis | 91 | 60 | 66 | 17-82 | 5 | 180 | Lung (74, 40%) | Melanoma (61, 32.9%) | Kidney (16, 8.6%) |
| polychondritis | 1 | 0 | - | 79 | 0 | 1 | - | - | - |
| polymyalgia rheumatica | 62 | 41 | 74 | 38-93 | 3 | 122 | Melanoma (47, 37.6%) | Lung (40, 32.0%) | Kidney (10, 8.0%) |
| polymyositis | 46 | 13 | 69 | 45-91 | 15 | 51 | Lung (25, 37.8%) | Melanoma (17, 25.7%) | Kidney (11, 11.6%) |
| psoriatic arthropathy | 47 | 6 | 67 | 20-82 | 4 | 61 | Lung (32, 49.2%) | Melanoma (11, 16.9%) | Kidney (9, 13.8%) |
| pubis fracture | 3 | 2 | 64 | 58-75 | 2 | 3 | Lung (3, 60.0%) | Melanoma (2, 40.0%) | - |
| radius fracture | 2 | 1 | 75 | 70-79 | 1 | 3 | Lung (2, 50.0%) | Head and neck (1, 25.0%) | Oesophageal (1, 25.0%) |
| resorption bone increased | 2 | 0 | - | 52-72 | 2 | 0 | - | - | - |
| rhabdomyolysis | 172 | 58 | 70 | 31-91 | 93 | 168 | Lung (81, 31.0%) | Melanoma (67, 25.6%) | Kidney (48, 18.3%) |
| rheumatoid arthritis | 131 | 86 | 66 | 18-90 | 19 | 311 | Lung (142, 43.0%) | Melanoma (70, 21.2%) | Kidney (16, 4.8%) |
| rib fracture | 24 | 9 | 71 | 51-89 | 3 | 41 | Lung (25, 56.8%) | Melanoma (6, 13.6%) | Head and neck (3, 6.8%) |
| scapula fracture | 2 | 1 | 52 | 48-68 | 0 | 3 | - | - | - |
| seronegative arthritis | 21 | 3 | 71 | 38-83 | 1 | 27 | Lung (10, 35.7%) | Melanoma (8, 28.5%) | Kidney (3, 10.7%) |
| Sjogren's syndrome | 69 | 54 | 67 | 26-84 | 1 | 146 | Lung (56, 38.0%) | Melanoma (40, 27.2%) | Kidney (24, 16.3%) |
| skull fracture | 2 | 1 | 70 | 68-76 | 1 | 2 | - | - | - |
| skull fractured base | 1 | 1 | - | 68-76 | 1 | 1 | - | - | - |
| spinal column stenosis | 5 | 2 | 72 | 50-84 | 0 | 7 | Lung (2, 28.5%) | Melanoma (2, 28.5%) | Kidney (2, 28.5%) |
| spinal compression fracture | 33 | 19 | 72 | 43-90 | 16 | 38 | Lung (32, 59.2%) | Melanoma (4, 7.4%) | Kidney (3, 5.5%) |
| spinal cord compression | 32 | 10 | 61 | 25-90 | 18 | 33 | Lung (14, 27.4%) | Melanoma (14, 27.4%) | Kidney (8, 15.6%) |
| spinal cord injury | 4 | 1 | 70 | 59-85 | 3 | 3 | Lung (3, 50.0%) | Melanoma (1, 16.6%) | Kidney (1, 16.6%) |
| spinal fracture | 16 | 11 | 62 | 22-83 | 7 | 28 | Lung (19, 54.2%) | Melanoma (4, 11.4%) | Kidney (3, 8.5%) |
| spinal ligament ossification | 1 | 0 | - | 77 | 1 | 0 | - | - | - |
| spinal osteoarthritis | 13 | 6 | 67 | 28-83 | 5 | 15 | Lung (7, 35.0%) | Melanoma (4, 20.0%) | Kidney (1, 10.0%) |
| spinal shock | 2 | 0 | - | 64 | 2 | 0 | - | - | - |
| spinal stenosis | 12 | 4 | 70 | 28-82 | 3 | 14 | Melanoma (6, 35.2%) | Lung (5, 29.4%) | Kidney (3, 17.6%) |
| spondyloarthropathy | 0 | 1 | - | 56 | 0 | 2 | - | - | - |
| sternal fracture | 3 | 1 | 68 | 61-78 | 0 | 4 | Lung (2, 50.0%) | Melanoma (1, 25.0%) | Kidney (1, 25.0%) |
| stress fracture | 2 | 2 | 65 | 55-72 | 0 | 5 | Melanoma (2, 50.0%) | Kidney (1, 25.0%) | Oesophageal (1, 25.0%) |
| temporomandibular joint syndrome | 1 | 1 | - | 68-75 | 1 | 1 | - | - | - |
| tenosynovitis | 10 | 9 | 69 | 38-84 | 0 | 22 | Melanoma (11, 50.0%) | Lung (4, 18.1%) | Bladder (1, 4.5%) |
| thoracic vertebral fracture | 8 | 6 | 69 | 51-86 | 3 | 11 | Lung (8, 57.1%) | Melanoma (2, 14.2%) | Bladder (1, 7.1%) |
| tibia fracture | 5 | 0 | 54 | 15-70 | 0 | 5 | Lung (1, 20.0%) | Melanoma (1, 20.0%) | Stoma (1, 20.0%) |
| tooth fracture | 2 | 4 | 69 | 52-75 | 0 | 10 | Lung (5, 50.0%) | Melanoma (2, 20.0%) | Kidney (1, 10.0%) |
| traumatic fracture | 2 | 2 | 67 | 64-77 | 0 | 4 | Kidney (2, 50.0%) | Lung (1, 25.0%) | Oral (1, 25.0%) |
| ulna fracture | 0 | 1 | - | 71 | 0 | 1 | - | - | - |
| upper limb fracture | 3 | 10 | 65 | 37-90 | 4 | 11 | Lung (5, 33.3%) | Melanoma (4, 26.6%) | Kidney (1, 6.6%) |
| wrist fracture | 3 | 2 | 71 | 60-79 | 0 | 6 | Melanoma (3, 50.0%) | Lung (2, 33.3%) | Oesophageal (1, 16.6%) |

^a^Out of all reported cases of adverse events induced by ICIs, 132,351 were in males and 81,690 were in females.

^b^Out of all reported cases of adverse events induced by ICIs, 156,634 resulted in alive, while 58,891 resulted in death.

^C^Among all reported cases of adverse events induced by ICIs, the distribution of tumor types was as follows: lung (34.9%; n=75,406), melanoma (22.3%; n=48,109), kidney (9.7%; n=21,088), bladder (2.7%; n=5,963), head and neck (2.5%; n=5,438), stomach (1.9%; n=4,249), lymphoma (1.9%; n=4,178), liver (1.3%; n=2,840), breast (1.2%; n=2,787), and colorectum (1.2%; n=2,770).
