# Supplementary material for: Musculoskeletal adverse events induced by immune checkpoint inhibitors: a large-scale pharmacovigilance study
Source: Front Pharmacol. 2023 Oct 10;14:1199031. doi: 10.3389/fphar.2023.1199031 (PMC10595016; doi:10.3389/fphar.2023.1199031)
Supplement: Supplementary file 4 [file Table3.DOCX]

Supplementary Table S3. Additional information on ICIs-induced musculoskeletal adver events described as symptoms or disease summary descriptions.

| **ICIs** | **AEs** | **Cases (N)** |
| --- | --- | --- |
| Atezolizumab | joint swelling | 5 |
|  | joint dislocation | 4 |
|  | joint effusion | 4 |
|  | joint range of motion decreased | 3 |
|  | joint irrigation | 2 |
|  | joint stiffness | 2 |
|  | muscle rigidity | 5 |
|  | muscle haemorrhage | 2 |
|  | haematoma muscle | 1 |
|  | muscle swelling | 1 |
|  | musculoskeletal pain | 21 |
|  | musculoskeletal stiffness | 14 |
|  | musculoskeletal discomfort | 2 |
|  | spinal pain | 8 |
|  | spinal disorder | 1 |
|  | bone pain | 23 |
|  | bone disorder | 8 |
| Avelumab | muscle disorder | 1 |
|  | musculoskeletal pain | 1 |
|  | joint swelling | 5 |
|  | joint dislocation | 4 |
|  | joint effusion | 4 |
|  | joint range of motion decreased | 3 |
|  | joint irrigation | 2 |
|  | joint stiffness | 2 |
|  | bone pain | 1 |
|  | spinal disorder | 6 |
| Cemiplimab | joint swelling | 2 |
|  | musculoskeletal pain | 2 |
|  | musculoskeletal stiffness | 2 |
| Durvalumab | joint swelling | 11 |
|  | joint stiffness | 1 |
|  | joint warmth | 1 |
|  | musculoskeletal chest pain | 6 |
|  | musculoskeletal stiffness | 8 |
|  | musculoskeletal pain | 4 |
|  | musculoskeletal disorder | 1 |
|  | muscle disorder | 1 |
|  | muscle haemorrhage | 1 |
|  | bone pain | 7 |
|  | bone disorder | 1 |
|  | spinal pain | 1 |
|  | spinal disorder | 1 |
| Ipilimumab | musculoskeletal pain | 6 |
|  | musculoskeletal stiffness | 5 |
|  | musculoskeletal chest pain | 3 |
|  | muscle twitching | 5 |
|  | extraocular muscle disorder | 3 |
|  | muscle disorder | 3 |
|  | muscle injury | 1 |
|  | joint swelling | 6 |
|  | joint effusion | 2 |
|  | joint injury | 2 |
|  | joint stiffness | 2 |
|  | bone pain | 3 |
| Nivolumab | joint swelling | 178 |
|  | joint stiffness | 33 |
|  | joint effusion | 14 |
|  | joint injury | 9 |
|  | joint dislocation | 8 |
|  | joint abscess | 3 |
|  | joint lock | 2 |
|  | joint range of motion decreased | 2 |
|  | musculoskeletal pain | 297 |
|  | musculoskeletal stiffness | 127 |
|  | musculoskeletal chest pain | 68 |
|  | musculoskeletal discomfort | 14 |
|  | musculoskeletal disorder | 12 |
|  | muscle disorder | 21 |
|  | muscle tightness | 20 |
|  | muscle twitching | 15 |
|  | muscle strain | 10 |
|  | muscle fatigue | 8 |
|  | muscle necrosis | 7 |
|  | muscle rigidity | 6 |
|  | muscle discomfort | 5 |
|  | muscle injury | 5 |
|  | extraocular muscle paresis | 4 |
|  | muscle spasticity | 4 |
|  | extraocular muscle disorder | 3 |
|  | muscle swelling | 3 |
|  | respiratory muscle weakness | 3 |
|  | diaphragm muscle weakness | 2 |
|  | muscle enzyme increased | 2 |
|  | muscle strength abnormal | 2 |
|  | muscle tone disorder | 2 |
|  | muscle abscess | 1 |
|  | muscle contractions involuntary | 1 |
|  | muscle haemorrhage | 1 |
|  | smooth muscle antibody positive | 1 |
|  | bone disorder | 8 |
|  | spinal pain | 49 |
|  | spinal disorder | 22 |
|  | spinal deformity | 6 |
|  | spinal instability | 5 |
| Pembrolizumab | joint swelling | 47 |
|  | joint effusion | 9 |
|  | joint stiffness | 7 |
|  | joint dislocation | 5 |
|  | joint range of motion decreased | 4 |
|  | joint injury | 3 |
|  | joint contracture | 2 |
|  | joint fluid drainage | 2 |
|  | joint instability | 2 |
|  | joint arthroplasty | 1 |
|  | joint hyperextension | 1 |
|  | musculoskeletal pain | 85 |
|  | musculoskeletal stiffness | 29 |
|  | musculoskeletal chest pain | 19 |
|  | musculoskeletal disorder | 16 |
|  | musculoskeletal discomfort | 13 |
|  | muscle disorder | 28 |
|  | muscle twitching | 9 |
|  | muscle strain | 6 |
|  | extraocular muscle paresis | 5 |
|  | muscle injury | 4 |
|  | muscle tightness | 4 |
|  | extraocular muscle disorder | 3 |
|  | muscle haemorrhage | 3 |
|  | muscle rupture | 3 |
|  | diaphragm muscle weakness | 2 |
|  | muscle contractions involuntary | 2 |
|  | muscle fatigue | 2 |
|  | muscle rigidity | 2 |
|  | respiratory muscle weakness | 2 |
|  | muscle hypertrophy | 1 |
|  | muscle strength abnormal | 1 |
|  | smooth muscle antibody positive | 1 |
|  | muscle necrosis | 4 |
|  | spinal pain | 11 |
|  | spinal disorder | 5 |
|  | spinal deformity | 2 |
|  | bone pain | 55 |
|  | bone disorder | 9 |
|  | bone swelling | 2 |
