# Supplementary material for: Musculoskeletal adverse events induced by immune checkpoint inhibitors: a large-scale pharmacovigilance study
Source: Front Pharmacol. 2023 Oct 10;14:1199031. doi: 10.3389/fphar.2023.1199031 (PMC10595016; doi:10.3389/fphar.2023.1199031)
Supplement: Supplementary file 5 [file Table4.DOCX]

Supplementary Table S4. Baseline information of the major ICIs-induced musculoskeletal AEs.

| **Subgroups** | **Clinical characteristics** | **Atezolizumab** | **Avelumab** | **Cemiplimab** | **Durvalumab** | **Ipilimumab** | **Nivolumab** | **Pembrolizumab** |
| --- | --- | --- | --- | --- | --- | --- | --- | --- |
| Myositis | Age (year) |  |  |  |  |  |  |  |
|  | Median | 70 | 68 | - | 68 | 71 | 70 | 71 |
|  | Range | 30 - 90 | 56-81 | - | 58-80 | 34-83 | 14-90 | 30-91 |
|  | Sex |  |  |  |  |  |  |  |
|  | Male | 46 | 4 | 1 | 25 | 20 | 360 | 162 |
|  | Female | 26 | 3 | 0 | 10 | 7 | 124 | 81 |
|  | Outcome |  |  |  |  |  |  |  |
|  | Death | 18 | 2 | 4 | 10 | 4 | 137 | 80 |
|  | Alive | 67 | 5 | 1 | 26 | 25 | 307 | 156 |
| Myopathy | Age (year) |  |  |  |  |  |  |  |
|  | Median | 75 | - | - | 67 | 70 | 69 | 76 |
|  | Range | 65 - 83 | - | - | 58 - 86 | 64 - 90 | 25 - 90 | 45 - 87 |
|  | Sex |  |  |  |  |  |  |  |
|  | Male | 4 | 1 | - | 2 | 2 | 38 | 43 |
|  | Female | 4 | 0 | - | 3 | 3 | 23 | 9 |
|  | Outcome |  |  |  |  |  |  |  |
|  | Death | 2 | 0 | - | 1 | 0 | 15 | 14 |
|  | Alive | 6 | 1 | - | 5 | 4 | 56 | 35 |
| Myasthenia gravis | Age (year) |  |  |  |  |  |  |  |
|  | Median | 73 | 74 | - | 71 | 71 | 71 | 73 |
|  | Range | 54 - 91 | 67 - 75 | - | 51 - 80 | 63 - 84 | 34 - 91 | 30 - 93 |
|  | Sex |  |  |  |  |  |  |  |
|  | Male | 15 | 1 | - | 12 | 20 | 143 | 116 |
|  | Female | 13 | 2 | - | 12 | 4 | 75 | 49 |
|  | Outcome |  |  |  |  |  |  |  |
|  | Death | 15 | 1 | 1 | 11 | 2 | 80 | 50 |
|  | Alive | 19 | 2 | 2 | 13 | 25 | 169 | 104 |
| Lambert-Eaton myasthenic syndrome | Age (year) |  |  |  |  |  |  |  |
|  | Median | 70 | - | - | 68 | 72 | 74 | 76 |
|  | Range | 1. 77 | - | - | 41-75 | 70 -73 | 32-90 | 55-87 |
|  | Sex |  |  |  |  |  |  |  |
|  | Male | 6 | - | - | 4 | 1 | 21 | 14 |
|  | Female | 1 | - | - | 1 | 2 | 14 | 10 |
|  | Outcome |  |  |  |  |  |  |  |
|  | Death | 1 | - | 0 | 1 | 0 | 15 | 7 |
|  | Alive | 6 | - | 1 | 4 | 3 | 23 | 16 |
| Guillain-Barré syndrome | Age (year) |  |  |  |  |  |  |  |
|  | Median | 64.5 | - | - | 64.5 | 66.5 | 67 | 66 |
|  | Range | 51-91 | - | - | 62-76 | 38-83 | 37-98 | 25-85 |
|  | Sex |  |  |  |  |  |  |  |
|  | Male | 9 | 2 | - | 6 | 9 | 45 | 26 |
|  | Female | 13 | 0 | - | 4 | 2 | 20 | 10 |
|  | Outcome |  |  |  |  |  |  |  |
|  | Death | 5 | 1 | - | 0 | 5 | 17 | 4 |
|  | Alive | 19 | 1 | - | 10 | 9 | 56 | 28 |
| Chronic inflammatory demyelinating polyradiculoneuropathy | Age (year) |  |  |  |  |  |  |  |
|  | Median | 50 | - | - | 68 | 71 | 67 | 64 |
|  | Range | - | - | - | 63-73 | 70-72 | 27-85 | 51-69 |
|  | Sex |  |  |  |  |  |  |  |
|  | Male | 0 | - | - | 1 | 2 | 8 | 4 |
|  | Female | 1 | - | - | 1 | 0 | 5 | 1 |
|  | Outcome |  |  |  |  |  |  |  |
|  | Death | 0 | - | - | 0 | 4 | 0 | 0 |
|  | Alive | 1 | - | - | 2 | 0 | 13 | 5 |
| Arthritis | Age (year) |  |  |  |  |  |  |  |
|  | Median | 66 | - | - | 69 | 66 | 65 | 67 |
|  | Range | 38-93 | - | - | 50-88 | 46-74 | 13-90 | 23-90 |
|  | Sex |  |  |  |  |  |  |  |
|  | Male | 27 | 1 | - | 28 | 8 | 455 | 192 |
|  | Female | 31 | 2 | - | 22 | 12 | 275 | 128 |
|  | Outcome |  |  |  |  |  |  |  |
|  | Death | 5 | 0 | 0 | 4 | 1 | 29 | 22 |
|  | Alive | 72 | 3 | 6 | 40 | 27 | 765 | 298 |
| Fracture | Age (year) |  |  |  |  |  |  |  |
|  | Median | 68 | - | - | 70 | 75 | 69 | 73 |
|  | Range | 15-95 | - | - | 51-90 | 46-84 | 22-92 | 37-91 |
|  | Sex |  |  |  |  |  |  |  |
|  | Male | 41 | 0 | - | 17 | 11 | 233 | 68 |
|  | Female | 34 | 1 | - | 6 | 6 | 201 | 70 |
|  | Outcome |  |  |  |  |  |  |  |
|  | Death | 11 | 0 | 0 | 8 | 5 | 85 | 8 |
|  | Alive | 65 | 1 | 1 | 12 | 14 | 366 | 116 |
| Myelitis | Age (year) |  |  |  |  |  |  |  |
|  | Median | 61 | 72 | - | 70.5 | 58 | 60 | 58 |
|  | Range | 32-80 | 50-73 | - | 62-82 | 25-72 | 33-78 | 37-79 |
|  | Sex |  |  |  |  |  |  |  |
|  | Male | 8 | 0 | 0 | 2 | 3 | 31 | 9 |
|  | Female | 9 | 4 | 0 | 3 | 5 | 19 | 9 |
|  | Outcome |  |  |  |  |  |  |  |
|  | Death | 2 | 0 | 1 | 0 | 2 | 9 | 3 |
|  | Alive | 13 | 4 | 0 | 7 | 8 | 48 | 17 |
| Fasciitis | Age (year) |  |  |  |  |  |  |  |
|  | Median | 58 | - | - | - | 71 | 60.5 | 70 |
|  | Range | 48-68 | - | - | - | 71-73 | 35-89 | 43-94 |
|  | Sex |  |  |  |  |  |  |  |
|  | Male | 1 | 1 | 0 | 0 | 1 | 22 | 12 |
|  | Female | 1 | 0 | 0 | 0 | 0 | 14 | 3 |
|  | Outcome |  |  |  |  |  |  |  |
|  | Death | 0 | 1 | - | - | 0 | 5 | 1 |
|  | Alive | 3 | 0 | - | - | 3 | 35 | 23 |
| Rhabdomyolysis | Age (year) |  |  |  |  |  |  |  |
|  | Median | 67 | - | - | 68 | 70.5 | 70 | 71 |
|  | Range | 54-86 | - | - | 63-78 | 32-83 | 31-91 | 33-90 |
|  | Sex |  |  |  |  |  |  |  |
|  | Male | 14 | 0 | 0 | 5 | 18 | 105 | 50 |
|  | Female | 5 | 1 | 0 | 2 | 2 | 37 | 15 |
|  | Outcome |  |  |  |  |  |  |  |
|  | Death | 4 | 0 | - | 2 | 5 | 61 | 21 |
|  | Alive | 15 | 1 | - | 5 | 16 | 90 | 41 |
| Polymyalgia rheumatica | Age (year) |  |  |  |  |  |  |  |
|  | Median | 78 | - | - | 66 | 76 | 72 | 75 |
|  | Range | 73-78 | - | - | 61-77 | 66-83 | 38-81 | 54-91 |
|  | Sex |  |  |  |  |  |  |  |
|  | Male | 1 | 1 | 0 | 4 | 2 | 24 | 34 |
|  | Female | 2 | 1 | 0 | 2 | 1 | 15 | 28 |
|  | Outcome |  |  |  |  |  |  |  |
|  | Death | 0 | 0 | - | 0 | 0 | 1 | 2 |
|  | Alive | 6 | 2 | - | 6 | 3 | 40 | 65 |
